# Molecular Mechanisms of the Impaired Heparin Pentasaccharide Interactions in 10 Antithrombin Heparin Binding Site Mutants Revealed by Enhanced Sampling Molecular Dynamics

**DOI:** 10.3390/biom14060657

**Published:** 2024-06-04

**Authors:** Gábor Balogh, Zsuzsanna Bereczky

**Affiliations:** Division of Clinical Laboratory Science, Department of Laboratory Medicine, Faculty of Medicine, University of Debrecen, 4032 Debrecen, Hungary

**Keywords:** antithrombin, mutations, molecular dynamics, enhanced sampling simulation methods

## Abstract

Antithrombin (AT) is a critical regulator of the coagulation cascade by inhibiting multiple coagulation factors including thrombin and FXa. Binding of heparinoids to this serpin enhances the inhibition considerably. Mutations located in the heparin binding site of AT result in thrombophilia in affected individuals. Our aim was to study 10 antithrombin mutations known to affect their heparin binding in a heparin pentasaccharide bound state using two molecular dynamics (MD) based methods providing enhanced sampling, GaMD and LiGaMD2. The latter provides an additional boost to the ligand and the most important binding site residues. From our GaMD simulations we were able to identify four variants (three affecting amino acid Arg47 and one affecting Lys114) that have a particularly large effect on binding. The additional acceleration provided by LiGaMD2 allowed us to study the consequences of several other mutants including those affecting Arg13 and Arg129. We were able to identify several conformational types by cluster analysis. Analysis of the simulation trajectories revealed the causes of the impaired pentasaccharide binding including pentasaccharide subunit conformational changes and altered allosteric pathways in the AT protein. Our results provide insights into the effects of AT mutations interfering with heparin binding at an atomic level and can facilitate the design or interpretation of in vitro experiments.

## 1. Introduction

Antithrombin (AT) is a plasma glycoprotein that serves as one of the most important regulators of the coagulation cascade [1,2]. Like many members of the serpin superfamily, it can inhibit its targets using an irreversible mechanism. Its main targets are thrombin, factor X and IX, but to a lesser extent it is also capable of inhibiting factors XI, XII, and VII. AT has a highly specific binding site for a pentasaccharide sequence found in sulfated glycosaminoglycans such as heparin [3,4]. Heparin binding triggers a complex series of conformational changes that result in enhanced activity against FXa and FIXa [4,5,6]. Heparin chains can also increase the activity of AT against its targets by forming ternary complexes. This “bridge formation” mechanism is especially important for enhancing the thrombin inhibition of AT [4,7].

Based on the results of kinetic experiments and X-ray diffraction studies, the conformational activation mechanism of AT can be best described using a three-step model. In the first step, binding of the ligand triggers conformational changes near the heparin binding site, stabilizing the “initial” interaction. The interaction becomes tighter in the second step due to rearrangements both in the binding site and the hydrophobic core of the protein. In the third step, the elongation of helix D and expulsion of the N-terminal part of the reactive center loop (the “hinge” region) from its “locked position” in the “A” beta sheet results in full activation against FXa and FIXa.

Inherited AT deficiency is one of the most severe forms of thrombophilia. AT deficiency is classified into type I (quantitative) and type II (qualitative). In type II deficiency, the AT antigen levels are normal, but there is a reduced activity in functional tests, while type I is characterized by both reduced AT antigen and activity [8,9]. Type II AT deficiency is divided further into II RS (reactive site), II HBS (heparin binding site), and II PE (pleiotropic effect) subtypes based on the consequences of different mutations. In the II RS subtype the interaction with the target factors is impaired. The II HBS subtype affects the binding of heparinoids, while type II PE variants have pleiotropic effects. AT has two main glycoforms, alpha and beta. Alpha-AT is glycosylated on four Asn residues—Asn96, 135, 155, and 192—while beta-AT has no glycan on Asn135 [10]. (The same residues are numbered as Asn128, Asn167, Asn187, and Asn224 according to the currently accepted nomenclature). In the circulation, approximately 90% of the protein is in alpha and 10% is in beta form. However, beta-AT has considerably higher affinity for a pentasaccharide ref. [11], suggesting an important physiological role. The beta glycoform has been found to retain significant functionality in some variants with mutations in the heparin binding site, and this might partially explain the relatively milder phenotype in patients carrying these mutations in heterozygous form [12].

The currently available knowledge for the AT–pentasaccharide interaction was derived from X-ray diffraction studies, kinetic experiments, and more recently, molecular dynamics simulations. However, such structures can only provide indirect evidence for biochemical processes like allostery that are the consequences of the highly dynamic nature of protein conformation. In contrast, computational methods, especially molecular dynamics (MD) and analysis of MD results, can potentially reveal details that cannot be easily studied using these experimental techniques. MD based methods that provide advanced conformational sampling compared to “conventional” simulations, but do not require pre-defined collective reactions are particularly well-suited for such use. An example of such a technique is Gaussian Accelerated Molecular Dynamics (GaMD) [13]. However, the acceleration may not be sufficient to all systems of interest. Recently, new GaMD-based methods such as PepGaMD [14], LiGaMD [15], and LiGaMD2 [16] were published that provide an even better conformational sampling possible for protein–ligand or protein–peptide complexes. In such simulations a “region of interest” can be specified for which additional boost is applied. In a LiGaMD2 simulation, this region includes both the ligand and selected residues in the binding site of the protein. Antithrombin and its interaction with heparinoids has been the subject of several MD-based studies. Several aspects of it have been investigated, including the consequences of heparin binding to the conformation of AT [17,18], the role of Asn135 glycosylation [19], and the interaction with a ligand containing a different scaffold [20]. The choice of molecular mechanics force fields for the heparin pentasaccharide is on-trivial. Therefore, studies where the conformations of sulfated oligosaccharides in solution from MD simulations are compared to NMR data are of particular importance when modeling interactions of these molecules with proteins [21,22].

In a previous work [23], we studied the consequences of three AT mutations in detail: Antithrombin Budapest III (L99F) [24], Basel (P41L) [25], and Padua I (R47H) [26]. (The numbering for amino acids used in the current article starts at the first residue of the mature, secreted protein. To convert the numbering into the nomenclature recommended by the American College of Medical Genetics for variant classification [27], 32 should be added to the amino acid positions used in this article.) We performed simulation enhanced sampling MD technique, GaMD, and compared the results with in vitro analysis and laboratory findings in patients carrying the mutations. We found different molecular mechanisms in the background of altered AT–heparin interaction. It seemed that AT Padua had the strongest effect on AT in this aspect as it showed the slowest AT–heparin complex formation and the weakest interaction while molecular modelling supported these findings. AT Basel showed slower AT–heparin complex formation, however the stability of the complex was only slightly affected. ATBp3 showed the strongest AT–heparin interaction, however this variant also had a destabilizing effect on AT, indicating a quantitative component in the pathogenicity of this mutation. Using surface plasmon resonance, the K_D_ value of the WT protein was measured to be 6.4 × 10^−10^ M. The R47H mutant was the most affected among the investigated ones with a K_D_ of 1.08 × 10^−6^ M while L99F was the least affected (K_D_ = 2.15 × 10^−8^ M). The affinity of P41L to heparin was between the values for L99F and R47H (K_D_ = 7.64 ×10^−7^ M), however, it also affected the binding kinetics. To our knowledge, our study was the first publication where long MD simulations were performed for AT mutants located in the heparin binding site. Although laboratory parameters and results of biochemical experiments correlate well with in silico findings, clinical phenotype of patients is often more complex and thrombotic diseases are influenced by many genetic and environmental factors for us to be able to draw a clear conclusion on the association of clinical phenotype with in vitro and in silico experimental results. It was observed, however, that there were differences among type of thrombosis, patient age at first thrombotic episode, and the presence or absence of obstetrical complications among the different II HBS mutants.

**Figure 1:**
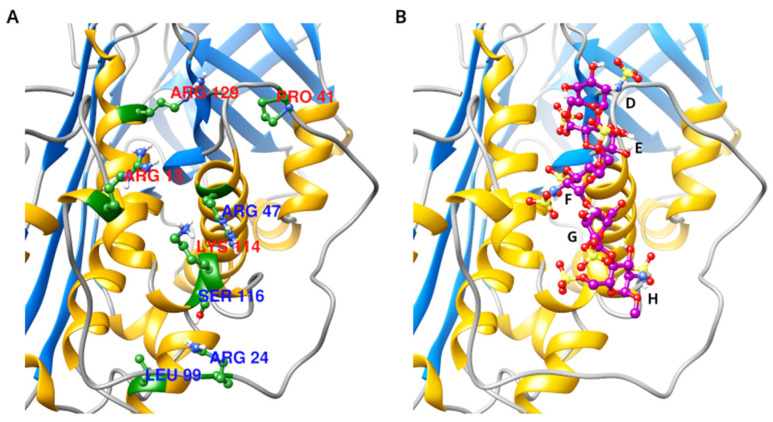
(**A**). The position of the amino acids in AT affected by the mutations simulated in the present study. (**B**). The position of the pentasaccharide ligand in the 3EVJ X-ray diffraction structure, with the five subunits of the pentasaccharide labelled (D–H).

The aim of the current study was to build in silico models for the selected 10 AT heparin binding site variants—R13W [28], R24C [29], P41L [25], R47C [30,31], R47H [26], R47S [32], L99F [24], S116P [33], K114E [34], and R129Q [35]—to study their consequences at an atomic level (Figure 1). All the mutations were classified as II HBS in the literature based on clinical laboratory findings or in vitro experiments. To achieve this, we applied a recently published enhanced sampling MD technique named LiGaMD2 which can boost the conformational sampling of both the ligand and the most important residues at the binding site of the protein. Although we had performed simulations for three mutations (L99F, P41L, and R47H) already, we showed that the LiGaMD2-based protocol provides new insights even for these variants. Being recurrent mutations, the clinical phenotypes of L99F, P41L, and R47H were described in more detail in clinical studies [36,37]. R13W mutation was rather associated with mild clinical phenotype according to literature data [28]. Mutations affecting positions 24, 47, 114, and 116 [28,31,33,38] were detected mainly in the background of arterial thrombotic events, and R47C mutation (AT Toyama) was detected in the homozygous state [30]—like L99F, AT Budapest 3 [36]—causing recurrent thrombosis. Crossed immunoelectrophoresis experiments were published for mutations R13W, R24C, P41L, R47C, R47H, L99F, K114E, and S116P, which indicated abnormal heparin binding of mutant AT. Experimental techniques, such as surface plasmon resonance, can provide quantitative data on the binding affinity and kinetics; our group has published such data for L99F, P41L, and R47H [23]. As most AT heparin binding site mutations are known exclusively in heterozygous patients (L99F is a notable exception), such measurements could not be executed in plasma samples of patients, and this can be investigated only in recombinant systems. Such experiments are often not performed. Moreover, the available in vitro assays are not able to investigate the AT–heparin interaction in more detail.

## 2. Materials and Methods

### 2.1. Preparation of Model Systems

We prepared AT-fondaparinux complex model systems for studying the following mutations affecting heparin binding: R13W, R24C, P41L, R47C, R47H, R47S, L99F, S116P, K114E, and R129Q. All model systems were based on the X-ray diffraction structure 3EVJ [39]. We used MODELLER 9.12 to generate initial conformations for missing loops. We selected fondaparinux as the pentasaccharide ligand, as this molecule is present in the X-ray structure [39]. For each mutant, as well as the wild-type protein, we built two models, one corresponding to the alpha and one to the beta glycoform.

As the LiGaMD2 method is not currently compatible with non-AMBER force fields, we selected the AMBER 14SB [40] force field for the protein and the GAFF 1.8 [41] force field for the ligand. We performed electrostatic potential calculation using Gaussian 09 [42] at the HF/6-31G* level for the pentasaccharide and the partial charges were then calculated using the RESP method [43,44]. GAFF was chosen because the LiGaMD2 technique was originally developed for this force field. The glycans present on either three (beta) or four (alpha form) asparagine residues were truncated at two N-acetylglucosamine subunits. We modelled the glycosylations as modified Asn amino acids, and we obtained force field parameters for these modifications using the R.E.D. webserver [45] (q4md-forcefieldtools.org, accessed on 7 December 2023.). The truncated oligosaccharides allowed us to simulate the system in relatively small water boxes, which was critical for reducing the computational effort due to the large number of production simulations. We followed a similar approach in our previous AT simulations [18,23].

The systems were simulated in a truncated octahedral box filled with TIP3P [46] water molecules. The size of box was calculated so that there was always 12 Å between the protein and the edges of the box. We added Na^+^ and Cl^−^ ions to all models to neutralize the systems and to set the ionic strength to approximately 0.15 M. The topology files for Amber simulations were created using the LEaP program of AmberTools (AmberTools 2022, University of California, San Francisco, CA, USA).

### 2.2. Molecular Dynamics Simulations

We performed energy minimization on all model systems in two phases. The first phase consisted of 500 steepest descent and 1500 conjugate gradient steps. Restraints of 5 kcal/mol/Å restraints were applied to all protein and ligand atoms to relax the water box. The second phase was a 2000-step conjugate gradient minimization with the restraints removed.

The PMEMD.CUDA (Amber 2022, University of California, San Francisco, CA, USA) [47] program of the Amber [48,49] simulation package versions 2020 and 2022 were used for the MD simulations. The systems were heated to 310 K in a 2 ns simulation under constant volume and temperature (NVT) with restraints applied 2 kcal/mol/Å on the non-solvent atoms. This was followed by 2 ns pressure equilibration with the Berendsen barostat [50] with identical restraints. Before GaMD [13] and LiGaMD2 [16], all systems were simulated for 150 ns under constant pressure and temperature (NpT) for further equilibration. Temperature coupling was carried out using the Langevin thermostat, with a time constant (collision frequency) of 2 ps^−1^. The timestep for numeric integration was 2 fs. The simulations were performed under periodic boundary conditions. The Coulomb and Lennard-Jones cut-off was set to 10 Å and long-range electrostatic interactions were calculated using the Particle Mesh Ewald (PME) method [51].

Both GaMD and LiGaMD2 require an “equilibration” simulation to be performed before the production runs to determine the parameters used for the enhanced conformational sampling. These runs were 100 ns long for all simulated systems and both sampling techniques and, according to the usual GaMD simulation protocol, consisted of four steps. In the first 5 ns, no acceleration was applied on the system, and the 5–10 ns part of the simulations was used for collecting energy data. In the remaining part, the system was equilibrated using a GaMD boost potential calculated from the available data, the parameters of which were constantly updated in the remaining part of the simulation (except for the first 5 ns).

The production GaMD simulations were performed under NVT conditions, and they were 800 ns long. The sigma0P and the sigma0D parameters of GaMD were set to their default values of 6.0. In the 500 ns LiGaMD2 simulations, we applied dual-boost (total potential energy and dihedral energy) on the entire systems, as well as additional boost on the pentasaccharide and the most important AT residues (based on article [52]): 11–13, 47, 114, 125, 129. We performed two independent GaMD simulations for the beta but only one for the alpha glycoforms of all mutants. Only beta-AT was simulated using the LiGaMD2 method, due to the difficulty of conformational sampling of a glycan on Asn135. Also, a full oligosaccharide would be located very close to the heparin binding site and can potentially interact with the pentasaccharide itself or with amino acids close to the binding site.

In total, five production simulations were performed on all mutants plus the WT systems, two GaMD and two LiGaMD2 simulations for beta and one GaMD simulation for alpha-AT.

### 2.3. Trajectory Analysis

Trajectory analysis such as distance, root mean square deviation (RMSD), and root mean square fluctuation (RMSF) calculations and clustering were performed using CPPTRAJ [53] (AmberTools 2022, University of California, San Francisco, CA, USA, 2022.). The K-means algorithm was used for cluster analysis. The cluster analysis was performed separately for the GaMD and LiGaMD2 simulations of beta-AT. The software created four clusters which were numbered by the number of analyzed frames in the cluster. We used the “generalized correlation” method proposed by Lange and Grubmüller to detect correlated motions between alpha-carbon atoms in the protein [54].

## 3. Results

### 3.1. Position of the Pentasaccharide Ligand Relative to the Heparin Binding Site of AT

To evaluate the consequences of the mutations, we analyzed the binding mode of the pentasaccharide by calculating its RMSD compared to its position in the X-ray structure of WT AT. As there is in vitro and in silico evidence that the DEF subunits may bind earlier than the remaining G and H [3,4,18], we also analyzed the RMSD of the five subunits separately, which can provide insight into the binding mechanism of pentasaccharide to WT AT in more detail. In a previous work, we found the RMSD parameters suitable for identifying states where the ligand is most tightly bound [18]. In the calculation, only the ring atoms as well as the oxygen atoms linking the rings were taken into account.

First, we analyzed the RMSD of the AT compared to the first frame of each GaMD and LiGaMD2 simulations. The conformation of the protein was stable according to the results (Supplementary Figures S1–S3).

**Figure 2:**
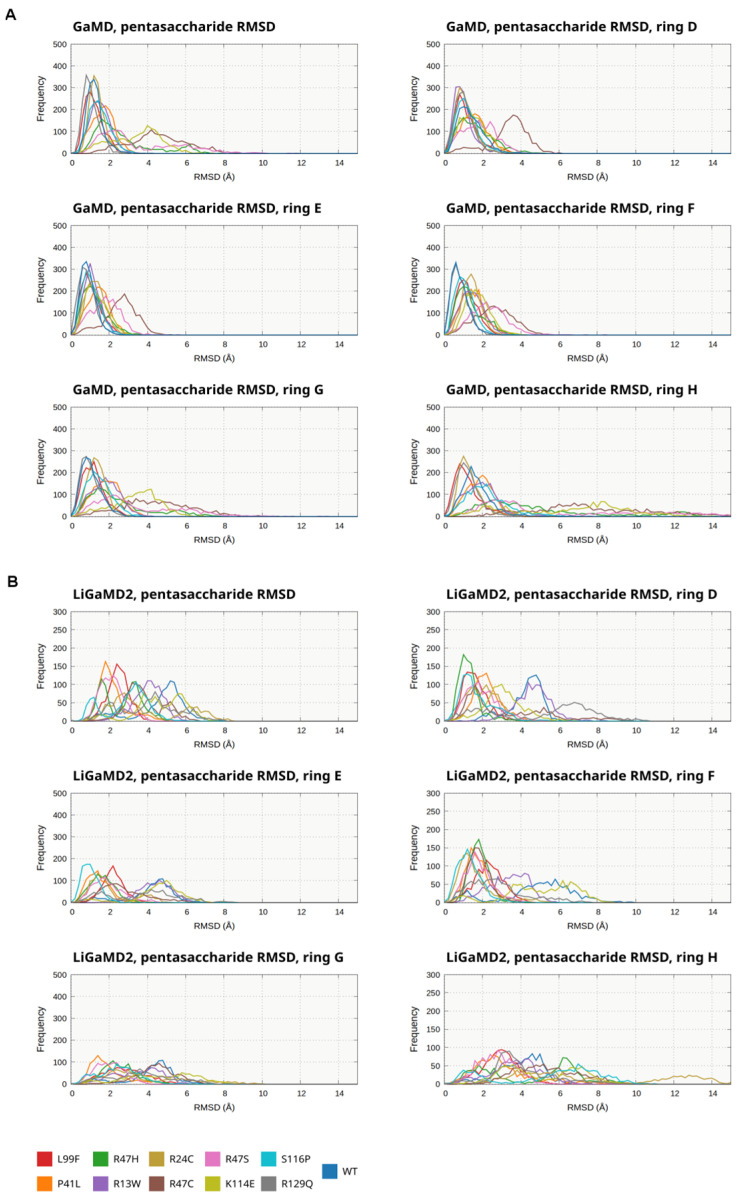
RMSD of the pentasaccharide compared to its position in the simulations of beta AT (WT or mutants), in the GaMD (**A**) and LiGaMD2 (**B**) simulations. The distribution of the various conformations is depicted as a histogram, the data from the two independent simulations for each mutant or the WT were combined. The results are shown for the whole pentasaccharide molecule as well as the D, F, and H subunits separately.

In the GaMD simulations of WT and nearly all mutated beta-AT variants (Figure 2A, Supplementary Figures S4–S7), the D and F subunits of the pentasaccharide and the RMSD values compared to the X-ray structures were low in most of the trajectories (below 3 Å). Slightly higher values were observed in the R47C and R47S simulations. In the H ring very large RMSD values were observed compared to the WT simulations in four mutants: R47C, R47H, R47S, and K114E. The RMSD for the whole ligand molecule tended to correlate with the values for the H subunit (as the largest values were mainly observed here). The tendencies were similar in the GaMD simulations of the alpha-AT systems (Supplementary Figures S8–S11); the same four mutations had a considerable effect on the binding of the H ring, while the D and F rings were less affected by the mutations.

In the LiGaMD2 simulations (Figure 2B, Supplementary Figures S12–S16), we could observe much higher RMSD values in each ring as well as in the full pentasaccharide compared to GaMD. Higher RMSD values for ring D were mainly present in the R13W, K114E, and R129Q simulations, and for the F ring in the simulations of R13W, R24C, and R129Q mutants. The conformation of ring H was the most affected by LiGaMD2. In contrast to the GaMD simulations, the maximum value was not between 1 and ~2.5 Å in any of the simulations on the RMSD histograms. The analysis revealed considerable differences in the conformational behavior for the mutants.

The simulations provide further support for our previous analysis that the GH end of the molecule can unbind more easily than the rest of the pentasaccharide and demonstrate that this mechanism also applies in the case of several AT variants with a mutated binding site. Using the LiGaMD2 technique, we were able to identify mutations severely affecting the heparin binding (R13W, R129Q), whose consequences were not evident from the same type of analysis of GaMD results. However, important limitations of the LiGaMD2 methodology are also evident from the data. The very wide distribution and the observation of high RMSD values also in the WT system make the comparison between the mutants more difficult despite the ability of the method of sampling states corresponding to partial dissociation.

The RMSD values between the two independent simulations for the same mutant can be compared using the time series data shown in Supplementary Figures S4–S7. The two replicas usually show similar tendencies for assuming conformations with higher RMSDs; however there are differences in some cases in the ratios of such frames between the two replicas.

To facilitate the quantitative comparison of the RMSD data, the fractions of frames from each simulation where the distance is above 3 Å are summarized in Supplementary Table S1 for the full pentasaccharide molecule and the subunits separately, from both the GaMD and LiGaMD2 simulations. We did not analyze the effect of the N-glycans of AT on the position of the pentasaccharide as such analysis would not be informative due to the truncated nature of the glycans.

### 3.2. Ring Conformations and Interglycosidic Dihedral Angles in the Pentasaccharide Ligand

The subunits of AT-binding pentassacharides are known to exhibit considerable conformational flexibility. Of particular importance is the conformation of ring G, which is a substituted iduronic acid. The ring conformations have been studied based on the available X-ray diffraction structures and more recently, by NMR measurements [21,22]. According to these results, the most favorable conformation of the G subunit is a “skewed boat” like form, but a chair conformation is also accessible. We analyzed the ring conformation of each subunit in the pentasaccharide ligand in our simulations using the Cremer–Pople theta parameter [55].

**Figure 3:**
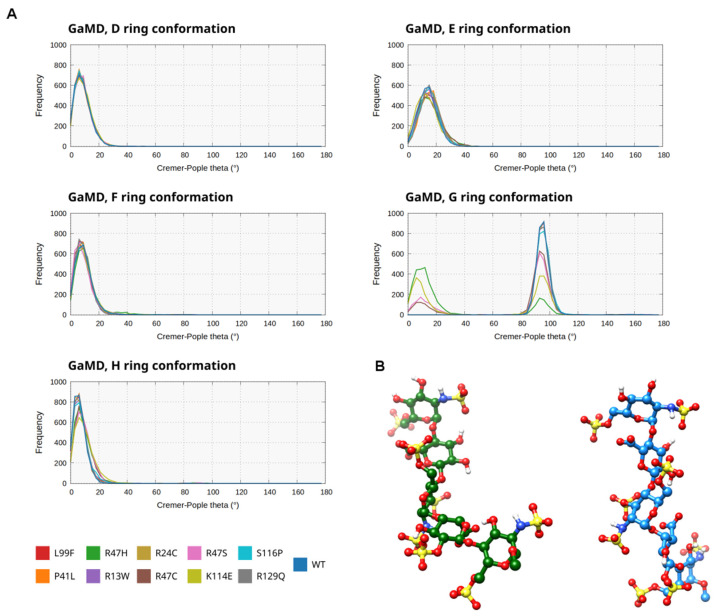
(**A**). The Cremer–Pople theta parameter for each subunit of the pentasaccharide ligand, which describes the conformation of each ring (~0°—4C1, ~180°—1C4, ~90°—boat like conformation). A histogram is shown for the simulations of each mutant as well as the wild-type protein in the GaMD simulations of beta AT. (**B**). Representative conformations for the pentasaccharide from the WT simulation (boat-like form) and from the R47H mutant affecting the conformation of ring G.

Among the GaMD simulations of beta-AT (Figure 3A, Supplementary Figures S16–S20), stable ring conformations were observed in all simulations for all rings except for the G subunit. The D-F and H subunits were in the 4C1 chair conformation in almost the entire trajectory according to the analysis. As for the G subunit, a boat-like state was predominantly detected in the WT as well as in the R13W, R47C, P41L, L99F, S116P, and R129Q trajectories. This finding is in agreement with our observations in our previous studies [18,21]. In contrast, in the simulations for the E114K mutant as well as the three mutants affecting the Arg47 residue, two main conformation types were observed, one similar to the state observed in the rest of the simulations, while also chair conformations were detected which were not observed elsewhere (Figure 3B). The ratio of the two conformations differed significantly from simulation to simulation. These findings possibly indicate a less favorable interaction with the “native” conformation of the pentasaccharide in case of mutations affecting positions 47 and 114, allowing more flexibility for the pentasaccharide molecule. In the GaMD simulations of alpha-AT (Supplementary Figures S21–S25), we could observe highly similar tendencies with mainly 4C1 chair conformation in rings D–F and H. In the the E114K and R47 mutant simulations, the G subunit was involved in conformational changes comparable to those observed in the beta AT simulations.

**Figure 4:**
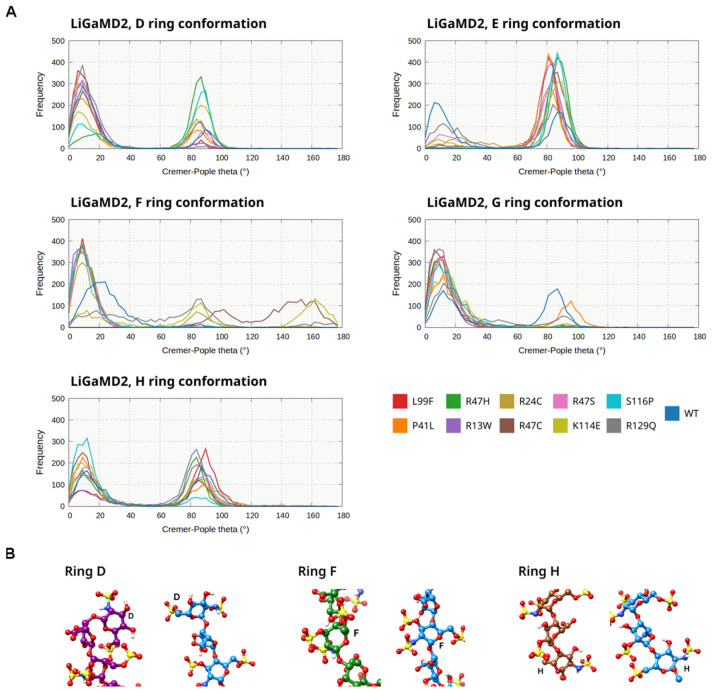
(**A**). The Cremer–Pople theta parameter for each subunit of the pentasaccharide ligand, which describes the conformation of each ring. A histogram is shown for the simulations of each mutant as well as the wild-type protein in the LiGaMD2 simulations of beta AT. (**B**). Representative conformations depicting “alternative” ring conformations that are different from the state observed in the GaMD simulations (4C1 boat), for rings D, F, and H (colored purple, green, and brown, respectively). The conformations are compared to a representative frame from the GaMD simulation (blue).

In the LiGaMD2 simulations (Figure 4A, Supplementary Figures S26–S30), where additional acceleration was applied to the pentasaccharide as well as selected residues of AT, the conformational behavior of the pentasaccharide subunits was markedly different. In the WT simulation and in mutants R13W, R24C, R47S, L99F, and R129Q the conformation of ring D was still predominantly as 4C1 chair, but with significantly increased flexibility. In the rest of the trajectories, a boat-like conformation was detected at a high frequency, but it was also present in the simulations with mainly a 4C1 form, similar to the GaMD simulation (Figure 4B). The behavior of ring F was somewhat similar: predominantly 4C1 chair form in WT, R13W, P41L, R47H, R47S, L99F, and S116P simulations. However, at least two alternative conformations were found, a boat-like in one of the R24C simulations, and a 1C4 chair in addition to the boat-like form in R47C and K114E. The conformation of the H ring fluctuated between the 4C1 chair and a boat-like form, but the latter was more prevalent in most simulations.

However, the analyses of the ring conformations from the advanced sampling simulations also have significant limitations, especially in LiGaMD2. This technique resulted in highly increased flexibility of the pentasaccharide, and in multiple mutants and conformations of the pentasaccharide subunits were observed, which were different from what was expected from the X-ray structures and solution NMR measurements. By using LiGaMD2 we found states which could correspond to the conformation of the ligand after partial dissociation from the binding site. To the best of our knowledge no experimental data are available for intermediates of the unbinding process, making the validation of the results difficult. However, the results may also represent limitations of the LiGaMD2 bias or the ligand force field. It is known that none of the available force fields for heparin pentasaccharides is able to perfectly describe the conformational ensemble of the rings [21,22].

To obtain an even more complete picture of the pentasaccharide conformation, we calculated the phi and psi dihedral angles for all four glycosidic bonds. (The definition of the dihedral angles can be found in an article by Sankaranarayanan et al. [56].) The results are shown as histograms in Supplementary Figure S31 for both the GaMD and LiGaMD2 simulations of beta-AT. In the GaMD simulations, the conformation of the D-E and E-F linkages were stable for all mutants as well as the WT and the histograms showed a narrow distribution of dihedral angles. Regarding the F-G and G-H interglycosidic dihedral angles, the behavior of four mutants was different from the WT and the remaining AT variants. The affected mutations were K114E and the three in amino acid position 47—the same mutants that showed conformational changes in ring G. The behavior of the systems was different in the LiGaMD2 simulations. The distribution of the dihedral angles was much wider and conformational types not observed in GaMD were visible for almost all mutant and interglycosidic linkages. In general, the WT simulations were the least affected but conformations with similar dihedral values to GaMD, although with a wider distribution, were observed for all linkages.

### 3.3. Analysis of Contacts between Antithrombin and the Pentasaccharide

From the output of the GaMD and LiGaMD2 simulations, we analyzed the involvement of individual positively charged amino acids in the binding. We calculated the distances between these residues (Lys11, Arg13, Arg46, Arg47, Lys114, Lys125, Arg129) and the negatively charged groups (O-sulfate, N-sulfate, carboxylate) groups of the pentasaccharide they can interact with. A cutoff of 5.0 Å was used to distinguish between snapshots in the trajectory, based on the presence or absence of the interaction. We chose a cutoff which is larger than the distance optimal for a hydrogen bond as we expected that the electrostatic component of the interaction was still significant for distances below this value. In Supplementary Table S2, the fractions of frames within the cutoff value are shown for selected interacting pairs, covering all five subunits of the pentasaccharide and the most important AT residues (K11, R47, K114, K125). The residues that show the largest differences compared to the WT systems were chosen. The percentages are for frames corresponding to the data from the two replicas combined, from both the GaMD and LiGaMD2 simulations (except for the simulation of alpha-AT where there were no replicas).

In the GaMD simulation of WT beta-antithrombin (Supplementary Figure S32A,B) the most stable contacts were between the O2 sulfate of the F subunit and Lys114, the carboxylate group of G subunit and Lys114, as well as the carboxylate of subunit E and Lys11. The contact involving the carboxylate group of the G subunit and Lys114 was present only in a fraction of sampled conformations in the R47C and R47S simulations and occurred with an even lower frequency for mutants R47H. (The K114E mutant could not be involved in such an interaction as residue 114 was mutated to an amino acid with the opposite charge). The interaction between the O2 sulfate of ring F and Lys114 was present in all simulations in the majority of analyzed frames except for K114E that affects the amino acid under analysis. The fraction of frames with no interaction was somewhat larger in the R47C and R47S trajectories as compared to the WT system. This indicates that mutations of amino acid 47 have a significant effect on the binding of the GH end of the pentasaccharide to AT.

The interaction between the O3 sulfate of the F subunit and Arg47 was also stable in all cases except for the four mutants that also highly affect other interaction (R47C, R47H, R47S, and K114E). Interestingly, this sulfate group can interact with residues not involved in binding in the WT simulation when this contact is absent. These include Arg46 and Lys11 in the R47C and R47 mutants and Lys125 in the K114E variant. The most important interaction of the ligand H subunit was between its S2 sulfate group and Arg47. This contact was present in almost the entire simulation for all systems except for K114E and the mutants affecting Arg47. The contact between the S6 sulfate and Lys125 was present in all simulations except for K114E, which mutation affects the same amino acid.

In the GaMD simulations of alpha AT (Supplementary Figure S33), the WT system as well as the R13W, R24C, P41L, L99F, and R129Q displayed similar conformational behavior to the corresponding beta AT simulations. The four mutations that affected the binding significantly in the beta AT simulations (R47C, R47H, R47S, and S116P) had an even larger effect in the simulations of alpha AT. The GH end of the pentasaccharide molecule interacted only in a fraction of the simulated conformations.

In the LiGaMD2 simulations (Supplementary Figure S34) additional boost was applied to both the ligand and binding site residues. As a result, the systems could also adopt conformations not sampled in GaMD, providing additional insights into the consequences of mutations. In general, the most stable interaction was formed by the Lys125 residue of AT and S3 sulfate of G subunit of the ligand; however, in a few simulations (notably K114E) even this interaction was significantly affected. Another interaction present in the large majority of mutants involved the carboxylate group of G and Lys114. Some mutants showed distinct conformational behavior compared to WT. For this reason, the mutants having the largest effect on the binding are discussed separately.

As a significant difference from the GaMD simulations, LiGaMD2 showed a loss of several interactions compared to the WT system in the trajectory for the R13W mutant. In these simulations the D ring interacted only with R129 in the majority of frames, and ring E was involved in only very weak contacts with all the analyzed AT residues. The interactions of the F ring were also mostly absent except for O3 sulfate of the pentasaccharide and K125 in AT.

The pentasaccharide interactions of R47C were also highly affected, almost all contacts formed by ring E and the most important contacts of ring F—with K114 and R13—were almost absent. The R47S mutation also severely affected the binding of the pentasaccharide in the LiGaMD2 simulation. Here, the F ring had significant contacts with ring F and the interactions involving K11 or R13 in AT were detected only in a small fraction of frames or not at all. The K114E amino acid substitution also had major effects on the pentasaccharide binding. Although no interaction except those involving the K114 residue was completely absent, contacts within a distance of 5 Å were detected much less frequently for the rings G and H of the pentasaccharide.

The altered contacts between the heparin-binding residues of AT and the subunits of the pentasaccharide are summarized in Table 1 for each mutation, for both the GaMD and LiGaMD2 simulations. In summary, due to the additional boost potential, we were able to detect losses of multiple interactions using the LiGaMD2 simulations in several mutants where GaMD did not show significant difference compared to the WT. However, due to the limitations of the LiGaMD2 simulations discussed in Section 3.2, we cannot rule out that the interaction losses may be caused by the unexpectedly high flexibility of the pentasaccharide in these simulations.

### 3.4. Conformation Changes in the Heparin Binding Site of Antithrombin

The activation of AT involves conformational changes near the binding site. Mutations at the surrounding regions of AT can have an effect on the conformation, resulting in impaired heparin binding. The helix P contains the Lys114 amino acid, one of the critical residues for binding the pentasaccharide. This helix is formed in one of the earlier steps of the activation mechanism. This change is required for the correct positioning of Lys114. In contrast, the elongation of helix D is one of the last events of the conformational activation process as suggested by the X-ray diffraction studies. We analyzed the secondary structure in region 111–139, which includes helices P (113–118) and D (120–134) using the DSSP method [57].

**Figure 5:**
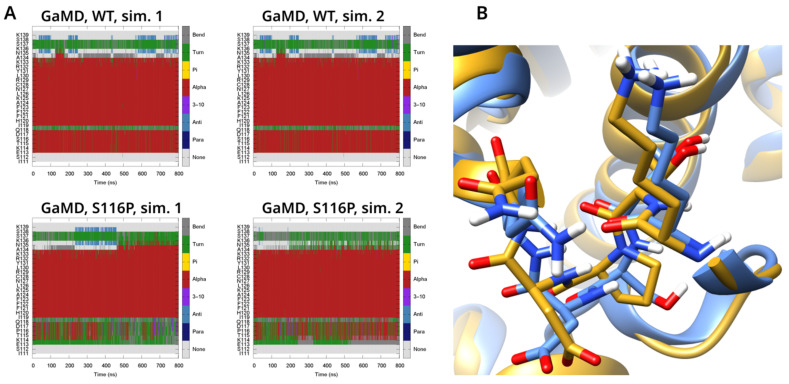
(**A**). Result of the DSSP analysis of the WT protein and the S116P mutant for residues 111–139. In the S116P mutant a conformational change in the O helix is visible (**B**). Comparison of the conformation of helix P between representative frames for the S116P mutant (yellow) and the WT protein (blue).

In the GaMD simulations (Supplementary Figure S35), the differences between the WT and most mutant systems were relatively minor. The S116P mutation, which affects one residue of helix P, was an exception however. The corresponding region was detected as helical only in less than half of the analyzed frames. (Figure 5A,B). Increased conformational flexibility on a significantly smaller scale near residue 117 was observed in three further simulations, R47C, R47S, and K114E. In most of the simulations, no significant elongation of helix D was visible; in most cases the end of the helix was at residue 134 or 135, with temporary conformational changes towards a shorter helix in R47H, L99F, and K114E. The observations were similar in the GaMD simulations of beta-antithrombin (Supplementary Figure S36).

In multiple GaMD simulations of AT mutants, the conformation of the C-terminal end of the helix was significantly altered, and this was most apparent in the R24C, P41L, and R47S, while in other cases the difference was smaller as compared to the WT system and the GaMD simulations. Interestingly, none of these mutations is located in helix D and therefore our results suggest an indirect mechanism similar to allosteric processes between the affected amino acids and the D helix.

In the LiGaMD2 simulations for AT (Supplementary Figure S37) the region corresponding to helix P underwent significant conformational changes. In nearly all simulations, this region was no longer detected as helical in the majority of the analyzed states. However, helical conformations were still present in the WT, R13W, P41L, and R129Q simulations.

### 3.5. Cluster Analysis

To identify conformational types among the various binding conformations observed for the pentasaccharide, we performed cluster analysis. The root mean square deviations (RMSD) between the positions of the pentasaccharide in the analyzed frames were used as distance metric for the clustering. (Before the distances were calculated, we performed alignment on the alpha-carbon atoms of the pentasaccharide.). Similar to the pentasaccharide RMSD analysis, only the coordinates of the ring and linker atoms were taken into account. We performed this analysis for the GaMD and the LiGaMD2 simulations separately. The WT AT and all mutants for one method were investigated in a single analysis. The clusters were numbered by the fraction of frames in them; therefore the same cluster number for GaMD and LiGaMD2 does not imply a similar conformation.

**Figure 6:**
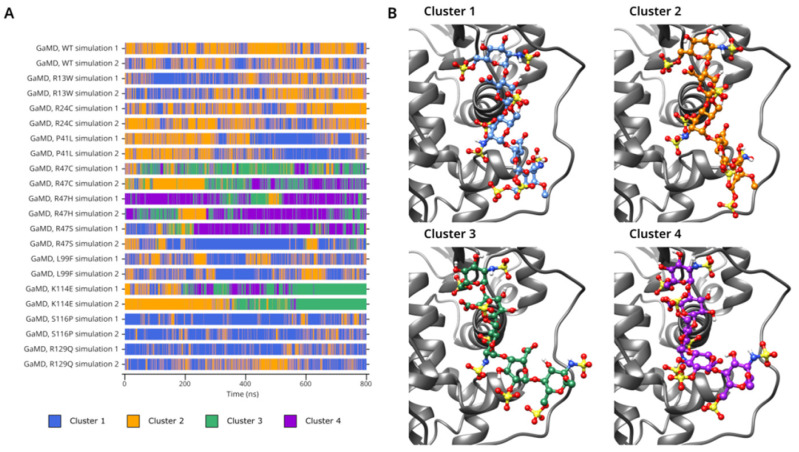
(**A**). Cluster number, as a function of time in the GaMD simulations of the WT protein and all mutants. (**B**). Representative frames for each conformational cluster.

In the cluster analysis of beta AT simulations (Figure 6A,B), Cluster 3 and 4 were almost exclusively present in the R47C, R47H, R47S, and K114E trajectories, corresponding to the same simulations where the most significant partial dissociation of the pentasaccharide was observed. The representative structures of these clusters suggest that they contain the states where the GH end of the pentasaccharide has undergone partial dissociation from the protein. In the rest of the simulations, nearly all frames were classified either as Cluster 1 or 2. The ratio of the frames among these two clusters varied widely among the simulations, with higher numbers of Cluster 2 simulations in WT and lower in L99F and S116P. The analysis demonstrates rapid conformational transitions between these two clusters which suggests a low energy barrier between these states. Generally, Cluster 2 was more prevalent in those simulations in which a relatively smaller effect on the binding of the pentasaccharide was observed in the GaMD simulations (R24C, P41L, L99F).

**Figure 7:**
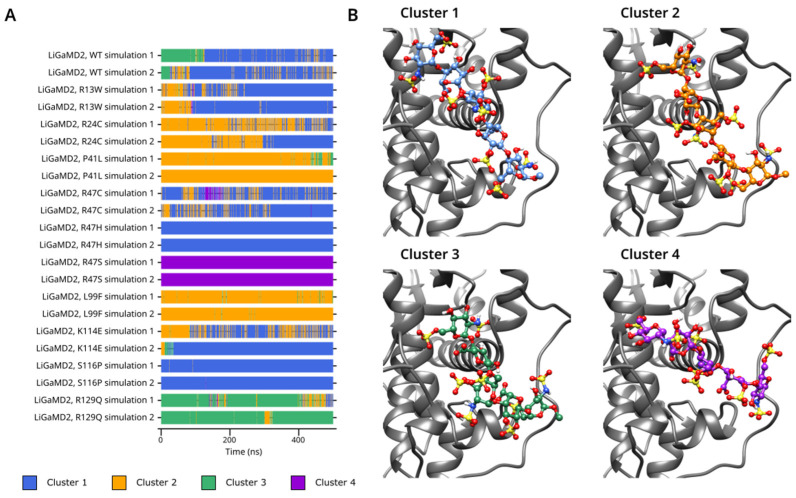
(**A**). Cluster number, as a function of time in the LiGaMD2 simulations of the WT protein and all mutants. (**B**). Representative frames for each conformational cluster.

Cluster analysis from the output of LiGaMD2 (Figure 7A,B) simulations allowed us to classify the analyzed mutants into multiple groups. In the L99F, P41L, and R24C simulations, Cluster 2 was predominant, but there was also a significant fraction of Cluster 1 conformations in the latter. In simulations R13W, R47C, R47H, K114E, and S116P the majority of the conformations belonged to Cluster 1. The conformations detected in the LiGaMD2 simulation of the R129Q and R47S systems were quite different from those observed in all other simulations; almost the entire trajectory of these simulations was placed into separate clusters, 3 and 4, respectively. These two clusters were almost entirely absent from all other simulations. The cluster analysis from the GaMD and especially LiGaMD2 simulations revealed the high conformational variability of the pentasaccharide in states with weak interaction between the protein and the ligand. Such conformations could serve as theoretical models for an early phase of the ligand binding and how mutations in AT can potentially affect this process. The frequent conformational changes between the clusters seen in the GaMD simulations were not observed in LiGaMD2; this is probably due to the much higher differences in the position of the pentasaccharide and the much larger conformational space within one cluster.

### 3.6. Root Mean Square Fluctuations

**Figure 8:**
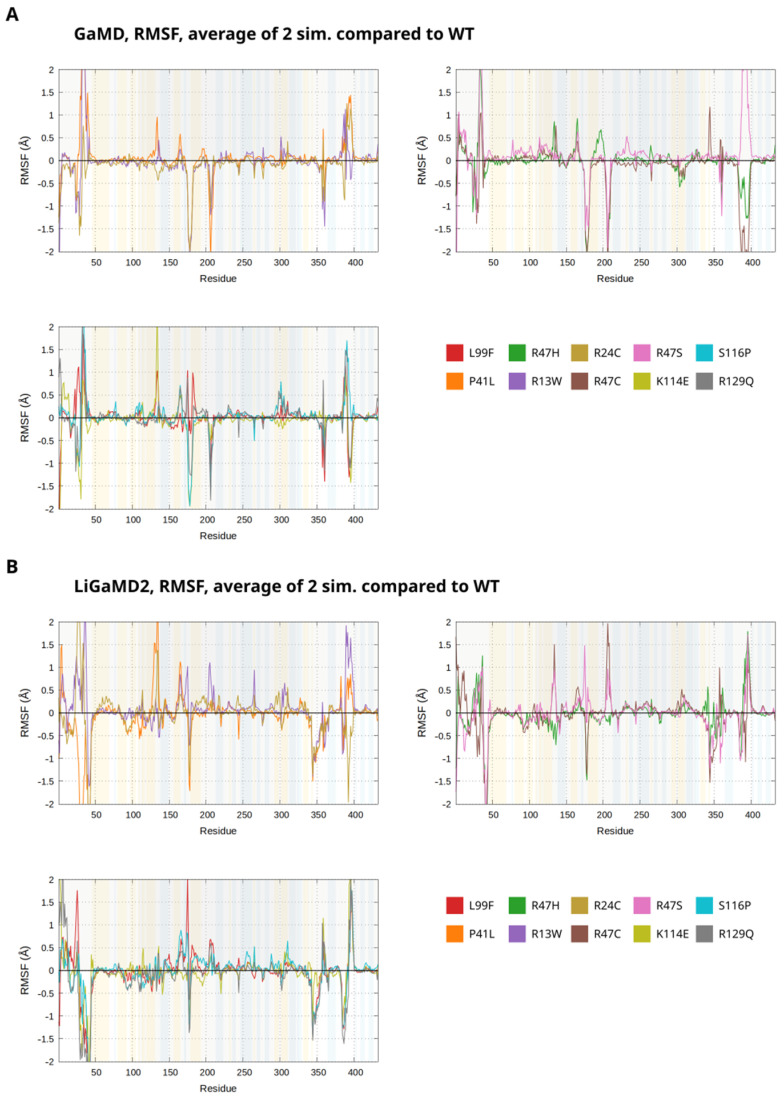
Root mean square fluctuations of the alpha-carbon atoms calculated from the GaMD and LiGaMD2 simulation of each mutant, compared to the simulations of the WT system. The RMSF values calculated from the two independent simulations of each system were averaged and the difference from the average calculated for WT was plotted as a function of residue number.

Root mean square fluctuations (RMSF) calculated for the alpha-carbon atoms in proteins are commonly used as a measure of flexibility of the protein backbone at each residue. Previously, we found that this parameter is suitable for detecting parts of the protein involved in conformational activation and also for detecting destabilization effects of mutants. We calculated the average RMSF values for each alpha-carbon atom for each mutant and plotted the difference compared to the WT simulations. We performed this analysis for both the GaMD and the LiGaMD2 simulations (Figure 8).

The N-terminal part of AT contains a highly flexible loop between residues 22 and 45. For multiple mutants, the RMSF calculations from the GaMD simulations suggested significantly different patterns as compared to the WT system in the conformational behavior (Figure 8A). The R13W, P41L, R47S, S116P, and to a lesser extent R47H and R129Q simulations showed increased fluctuations in this region, suggesting an effect of these mutations on the conformational behavior. In the 110–140 region, which contains several residues involved in heparin binding, moderate increases compared to the WT system were observed in the case of the R47S and R47C mutants. This suggests that these mutants have a destabilizing effect for the conformation of the protein at this site. The K114E mutant showed a very large peak near the end of this region (amino acids~130–140) where the D helix elongation takes place. In the 180–200 region, the R47H simulation showed large fluctuations but increases in the RMSF were also visible in the P41L, R47S, K114E, S116P, and R129Q systems. In the 230–260 region of the protein, several residues are part of an “exosite” involved in the interaction of AT with FXa and FIXa [6,58]. Thus, increased or decreased fluctuations at these amino acid positions could suggest that allosteric processes between the heparin binding site and the “exosite” may be affected by a mutation. In case of the R47S mutant, significant increases in RMSF are visible at many of the amino acids between 220 and 240. This is probably the result of the destabilizing effect of this mutant on the protein also seen in other parts of the molecule.

The averaged RMSF values calculated from the LiGaMD2 simulations revealed differences in conformational behavior when compared to the GaMD simulations of the same mutants (Figure 8B, Supplementary Figure S38). This is an expected behavior of the systems as the binding of the pentasaccharide became weaker in all simulations, including the WT, and this change probably has allosteric effects on other regions. The P41L mutant, located in the 22–45 loop exhibited a large effect on the conformational behavior of that loop. Surprisingly, the LiGaMD2 simulations showed a significantly increased instead of decreased RMSF in this region. This can be explained by the formation of a helical structure at amino acids 29–34. The Glu34 residue is positioned close to the Arg46 side chain that is normally involved in the pentasaccharide binding and can form a salt bridge with it. Therefore, this loop conformation can potentially impair the ligand binding. Somewhat similar effects on the conformation of this loop were also visible in the LiGaMD2 simulations of other mutants, K113E and R129Q and to a lesser extent, L99F and S116P.

### 3.7. Analysis of Regions Involved in Allosteric Precesses

The conformational activation of AT is essentially an allosteric mechanism in which the binding of the high affinity pentasaccharide to the heparin binding site leads to altered interaction with its target factors at a distant interaction site. Previously, we found the analysis of correlated motions between alpha-carbon atoms of the protein to be an efficient method for detecting amino acids involved in such processes [18,23]. Here, we used a method proposed by Lange and Grubmüller to detect correlated motions between the alpha-carbon atoms in the protein. This technique has advantages over dynamic cross-correlation matrices which can detect only the linear component of such movements. We performed this analysis on the alpha-carbon atoms in both the GaMD and LiGaMD2 simulations.

Correlated motions between the heparin binding site, and the N-terminal part of the reactive center loop are of particular importance. This fragment of the molecule is inserted into the A helix in the inactive state of the protein and pentasaccharide binding leads to the expulsion of the hinge region from its closed position through allosteric mechanisms. Also, the region around amino acids 230–240 contains several important residues that play a role in the enhanced interaction of AT against FXa and FIXa in the activated state.

The matrices obtained for the GaMD simulation of the WT systems show correlated motions between the regions that form the heparin binding site: amino acids 45–50 (including Arg46 and Arg47) and 100–130 (Lys114, Lys125, Arg129) (Supplementary Figure S39A,B). We could detect a correlation between both of these sites and the reactive center loop of the protein (amino acids 375–399). This suggests a potentially important allosteric pathway that connects the ligand binding site and the reactive site. In multiple mutants, including R47S, K114E, S116P, and R129Q, the analysis revealed regions with increased correlation values, especially at amino acids 250–320 as compared to the WT simulations. These differences may suggest conformational changes towards a non-activated conformation of AT.

In the LiGaMD2 simulations of the WT system the generalized correlation matrices showed similarities with those obtained from the GaMD simulations of these systems, however, with somewhat increased correlations in the 250–320 region (Supplementary Figure S40A,B). As the LiGaMD2 simulations showed significant partial unbinding of the pentasaccharide even in the WT protein, this further supports the conclusion that this may indicate a transition from the active to the inactive state. In general, several mutants show considerably different patterns from the WT simulations; these are R13W, L99F, K114E, S116P, R129Q, and especially R24C. This suggests that the mutations affecting these residues have significant effects on the allosteric mechanisms and the additional boost from LiGaMD2 can help to detect such consequences of the mutations.

The methodology we used for the analysis of allostery also has limitations. It detects only correlated motions between residue pairs, and it is not suitable for reconstruction of pathways involving multiple residues or regions. More advanced techniques are available for the analysis of allosteric pathways, but in the present paper these methodologies were not used due to technical issues in comparing these data between multiple mutants and possible incompatibility with the advanced sampling technique.

## 4. Discussion

In this study, we performed enhanced sampling MD simulations for studying the consequences of 10 AT mutations known to affect interaction with heparinoids, R13W, R24C, P41L, R47C, R47H, R47S, L99F, S116P, K114E, and R129Q. Our aim was to create in silico models, which can describe the effects of these mutations at an atomic level. Simulation of mutants can also provide insights into the mechanism of conformational activation and the function of important amino acids involved in the biochemical process.

Due to the complex structure–function relationship in this protein, we selected two advanced sampling techniques, GaMD and LiGaMD2 to study the consequences of these mutations. Three of them, P41L, R47H, and L99F were already simulated by our group using a different GaMD-based protocol in a previous work. A significant limitation of that study was that we could only observe very limited dissociations of the pentasaccharide from its binding site when using our earlier protocol [23].

Our results in this study demonstrate the applicability of both techniques for gaining insights into the mechanisms at an atomic level. For the three mutants we simulated previously, we were able to confirm our previous results by using a different force field. Specifically, R47H has the most severe consequences among the three mutants, and L99F mainly affected the allosteric mechanisms while having a limited effect directly on the binding side residues. The GaMD simulations revealed that among the newly analyzed mutants, R47C, R47S, and K114E had the most significant consequences directly on the binding of the pentasaccharide to AT.

However, in the case of several mutants, especially for R13W and R129Q, the additional boost potential of LiGaMD2 was needed to reveal the mechanisms resulting in impaired interaction. Using the LiGaMD2, we were able to observe at least partial or even nearly total unbinding of the pentasaccharide. Although LiGaMD2 was able to suggest the mechanism behind the impaired binding for some mutants, this methodology also had significant limitations. The pentasaccharide RMSD values were highly increased for the WT systems and not just the mutants, making such comparisons not possible. The LiGaMD2 based protocol resulted in highly increased flexibility of the pentasaccharide ring conformations, and interglycosidic angles were observed that were different from both the bound state of the pentasaccharide in the X-ray structures and also the solution NMR measurements. These results may be partially explained by the fact that they were found in intermediate states of ligand binding but may also reflect shortcomings of the enhanced sampling method or the ligand force field.

The R13W mutation affects an amino acid close to the N-terminal of the mature AT protein. X-ray diffraction and mutagenesis studies confirmed the direct role of this arginine to the pentasaccharide binding. In the GaMD simulations, R13W was one of the mutants that had comparatively smaller effects on the ligand binding. The additional acceleration provided by the LiGaMD2 simulations, however, revealed the mechanism of the deleterious effects.

In the R24C mutant, an arginine close to the N-terminal of the 22–45 loop is substituted with cysteine. The positively charged amino acid in WT protein is capable of forming salt bridges with two negatively charged residues in or close to the P helix, Glu113, and Asp117. In GaMD, the behavior did not differ from WT significantly. However, the DSSP analysis showed a limited effect on helix P. In contrast, the same analysis from the LiGaMD2 results showed alterations at the C-terminal end of D helix, suggesting an indirect effect on the conformation of the binding site. The generalized correlation calculations showed effects on the allosteric pathways. However, we cannot rule out other potential mechanisms such as interference with the formation of the nearby 21–95 disulfide bond.

P41L is located close to the C terminal end of the same loop that contains the R24 amino acid. We previously suggested that the substitution of P41 in this loop can increase its flexibility, resulting in potentially unfavorable conformations for the pentasaccharide binding. In the GaMD simulations, it is one of the less affected variants as compared to the WT simulation. In the GaMD simulation, increased flexibility of the same loop could be observed. Surprisingly, the LiGaMD2 simulations showed a significant increase of RMSF instead of a decrease in this region. In the LiGaMD2 simulation, the conformation of helix D was also affected.

We investigated three mutants affecting amino acid R47 in the present work. One of them, R47H was simulated by our group previously with a different molecular dynamics protocol. Among the three mutations studied in this previous publication, R47H was the one that had the most severe consequences. Loss of several important AT–pentasaccharide interactions was evident in both the GaMD and LiGaMD2 results. In this study, we could confirm the severe consequences of R47H that we had found in our previous work.

R47C affects the same amino acid as R47H, and there are several similarities in the conformational changes of these two mutants. In GaMD, the RMSD values for the ligand positions were even more significantly affected than in the R47H mutant and it had one of the largest effects on the ring D conformation. In the LiGaMD2 simulations, interactions formed by the F and G subunits were detected only in a smaller number of states. The generalized correlations analysis revealed different patterns as compared to the WT system, suggesting an effect on the allosteric pathways.

R47S is the third mutation studied in this position. In the LiGaMD2 simulations, many interactions, especially those affecting the F ring, were observed at very low frequencies. The mutation had a significant effect on the conformation of the F subunit in the pentasaccharide in LiGaMD2, explaining the loss of contact as compared to the WT simulation. It also affected the C terminal end of helix D in LiGaMD2, despite this mutation not being located in this helix.

L99F is located in helix C and does not form immediate contacts with the heparin pentasaccharide. In the GaMD simulations, it had only small effects on the pentasaccharide binding comparing it to the WT system. Generalized correlation calculations from both the GaMD and LiGaMD2 simulations revealed that this mutant has significant effects on the allosteric pathways; the observed patterns differed significantly from the WT simulations. In the LiGaMD2 simulations, the interaction of the pentasaccharide with multiple AT residues was also altered.

K114E affects one of the most important pentasaccharide binding residues of AT that is located in helix P. This mutant seems to have one of the largest effects on pentasaccharide binding. The GH end of the pentasaccharide has a high tendency for partial unbinding even in the GaMD simulations. In the simulated states of the system showing this change, alternative conformations occurred with a high frequency for ring G in the GaMD and also for ring F in the LiGaMD2 simulations. Our in silico findings are in line with a previous in vitro study reporting an almost complete loss of heparin pentasaccharide binding.

S116P affects an amino acid located in the P helix and the Ser to Pro replacement can be expected to have a disruptive effect on the conformation of this structural element. This was confirmed by the DSSP analysis from the GaMD simulations showing no helix in this region. Despite the altered structure in this region, the effects of this mutant were less significant in the GaMD simulation than those affecting the R47 and K114 amino acids. The RMSF and generalized correlation analysis showed that this mutant also had an effect on the conformational behavior on distant parts of the protein.

The R129Q mutation affects an amino acid located in helix D, which mainly interacts with the D subunit of the pentasaccharide. In the GaMD simulations, this mutant did not show significant alteration of the pentasaccharide binding as compared to the WT system. The LiGaMD2 simulations revealed that several AT-ligand contacts, especially those formed by subunits F and H of the pentasaccharide, were weakened.

To validate the results experimentally, quantitative data on the AT–heparinoid interaction would be needed. Previously, our group found that K_D_ values determined using surface plasmon resonance to be a promising technique to obtain quantitative data on the strength of interaction between mutated AT and the ligand. Currently such data are available only for the P41L, R47H, and L99F mutations.

In summary, the consequences of the K114E and the three mutations affecting the R47 amino acids were the most severe. In contrast, R13W, R24C, P41L, and R129Q had effects that could be detected only by the more advanced LiGaMD2 method.

The results of in silico studies provided novel insights into the structure–function relationships in this protein. Such information can facilitate the design of in vitro experiments or explain the result of such studies and can be useful for drug discovery as well. The findings may potentially help to explain the differences between the laboratory findings and the clinical consequences for the various type II heparin binding site variants.

## Figures and Tables

**Table 1:** Altered contacts between the pentasaccharide and binding site residues in the AT in the simulations of the mutants compared to the WT.

Mutation	GaMD Simulations	LiGaMD2 Simulations
R13W	Similar to WT	Loss of most ring D, E, and F interactions, weak interactions with GH
R24C	Similar to WT	F subunit interaction with R47, K114 affected
P41L	Similar to WT	F subunit interaction with R46, K114 affected
R47C	Significantly weaker interaction with ring G Weaker interaction between ring F and K114 Almost no interaction between ring H and Arg47	Interactions of ring F almost completely lost with R46, R47, and K114E Weaker interaction with GH end of molecule
R47H	Significantly weaker interaction with ring G, Weaker interaction between ring F and K114 Almost no interaction between ring H and Arg47	Interactions of ring F almost completely lost with R46, R47, and K114E Interaction between ring G and K114 significantly affected
R47S	Significantly weaker interaction with ring G Weaker interaction between ring F and K114 Almost no interaction between ring H and Arg47	Interactions of ring F almost completely lost with R46, R47, and K114E Weaker interaction with GH end of molecule
L99F	Similar to WT	F subunit interaction with R47, K114 affected (weaker interactions of ring H)
K114E	Interactions with ring G became weaker Contacts with H subunits also significantly affected	Interaction with GH end of molecule weakened Interaction of Lys125 and Arg47 with rings E and F affected
S116P	Similar to WT	Interaction between ring F and K114 lost, interaction of ring D with K125 affected
R129Q	Similar to WT	No interaction lost completely, interactions of F and H subunit affected

## Data Availability

The datasets generated for this study are available upon request to the corresponding author.

## Supplementary Materials

The following supporting information can be downloaded at: https://www.mdpi.com/article/10.3390/biom14060657/s1, Figure S1. RMSD of the alpha-carbon atoms in the antithrombin protein, compared to the first frame of the simulations in the GaMD simulations of beta-antithrombin. Figure S2. RMSD of the alpha-carbon atoms in the antithrombin protein, compared to the first frame of the simulations in the GaMD simulations of alpha-antithrombin. Figure S3. RMSD of the alpha-carbon atoms in the antithrombin protein, compared to the first frame of the simulations in the LiGaMD2 simulations of beta-antithrombin. Figure S4. The RMSD of the pentasaccharide ligand compared to its position in the energy minimized structure, as a function of time, in the GaMD simulations of beta AT. Figure S5. The RMSD of ring D in the pentasaccharide ligand compared to its position in the energy minimized structure, as a function of time, in the GaMD simulations of beta AT. Figure S6. The RMSD of ring F in the pentasaccharide ligand compared to its position in the energy minimized structure, as a function of time, in the GaMD simulations of beta AT. Figure S7. The RMSD of ring H in the pentasaccharide ligand compared to its position in the energy minimized structure, as a function of time, in the GaMD simulations of beta AT. Figure S8. The RMSD of the pentasaccharide ligand compared to its position in the energy minimized structure, as a function of time, in the GaMD simulations of alpha AT. Figure S9. The RMSD of ring D in the pentasaccharide ligand compared to its position in the energy minimized structure, as a function of time, in the GaMD simulations of alpha AT. Figure S10. The RMSD of ring F in the pentasaccharide ligand compared to its position in the energy minimized structure, as a function of time, in the GaMD simulations of alpha AT. Figure S11 The RMSD of ring H in the pentasaccharide ligand compared to its position in the energy minimized structure, as a function of time, in the GaMD simulations of alpha AT. Figure S12. The RMSD of the pentasaccharide ligand compared to its position in the energy minimized structure, as a function of time, in the LiGaMD2 simulations of beta AT. Figure S13. The RMSD of ring D in the pentasaccharide ligand compared to its position in the energy minimized structure, as a function of time in the LiGaMD2 simulations of beta AT. Figure S14. The RMSD of ring F in the pentasaccharide ligand compared to its position in the energy minimized structure, as a function of time, in the LiGaMD2 simulations of beta AT. Figure S15. The RMSD of ring H in the pentasaccharide ligand compared to its position in the energy minimized structure, as a function of time, in the LiGaMD2 simulations of beta AT. Figure S16. The Cremer–Pople theta parameter for ring D in each GaMD simulation of beta AT, as a function of time. Figure S17. The Cremer–Pople theta parameter for ring E in each GaMD simulation of beta AT, as a function of time. Figure S18. The Cremer–Pople theta parameter for ring F in each GaMD simulation of beta AT, as a function of time. Figure S19. The Cremer–Pople theta parameter for ring G in each GaMD simulation of beta AT, as a function of time. Figure S20. The Cremer–Pople theta parameter for ring H in each GaMD simulation of beta AT, as a function of time. Figure S21. The Cremer–Pople theta parameter for ring D in each GaMD simulation of alpha AT, as a function of time. Figure S22. The Cremer–Pople theta parameter for ring E in each GaMD simulation of alpha AT, as a function of time. Figure S23. The Cremer–Pople theta parameter for ring F in each GaMD simulation of alpha AT, as a function of time. Figure S24. The Cremer–Pople theta parameter for ring G in each GaMD simulation of alpha AT, as a function of time. Figure S25. The Cremer–Pople theta parameter for ring H in each GaMD simulation of alpha AT, as a function of time. Figure S26. The Cremer–Pople theta parameter for ring D in each LiGaMD2 simulation of beta AT, as a function of time. Figure S27. The Cremer–Pople theta parameter for ring E in each GaMD simulation of beta AT, as a function of time. Figure S28. The Cremer–Pople theta parameter for ring F in each LiGaMD2 simulation of beta AT, as a function of time. Figure S29. The Cremer–Pople theta parameter for ring G in each LiGaMD2 simulation of beta AT, as a function of time. Figure S30. The Cremer–Pople theta parameter for ring H in each LiGaMD2 simulation of beta AT, as a function of time. Figure S31. Interglycosidic dihedral angles in the pentasaccharide for all four glycosidic bonds from the simulations of beta AT. Figure S32. The amino acids of AT interacting with negatively charged groups in the pentasaccharide (distances below 5 Å) as a function of time, in the GaMD simulations of beta-antithrombin. Figure S33. The amino acids of AT interacting with negatively charged groups in the pentasaccharide (distances below 5 Å) as a function of time, in the GaMD simulations of alpha-antithrombin. Figure S34. The amino acids of AT interacting with negatively charged groups in the pentasaccharide (distances below 5 Å) as a function of time, in the LiGaMD2 simulations of beta-antithrombin. Figure S35. Result of the DSSP analysis for residues 111–139, close to the heparin binding site, from the GaMD simulations of beta AT. Figure S36. Result of the DSSP analysis for residues 111–139, close to the heparin binding site, from the GaMD simulations of alpha AT. Figure S37. Result of the DSSP analysis for residues 111–139, close to the heparin binding site, from the LiGaMD2 simulations of beta AT. Figure S38. Root mean fluctuations of the alpha-carbon atoms calculated from the simulations of each mutant, compared to the simulations of the WT system. The RMSF values for each CA atom were averaged from the two simulations for each mutant as well as the WT. The data for the mutants and the WT system are shown in red and blue, respectively. Figure S39. Correlated motions of the alpha-carbon atoms in the GaMD simulations of the WT AT protein and the mutants. A method proposed by Lange and Grubmüller was used for the analysis. Figure S40. Correlated motions of the alpha-carbon atoms in the LiGaMD2 simulations of the WT AT protein and the mutants. A method proposed by Lange and Grubmüller was used for the analysis. Supplementary Table S1. RMSD of the pentasaccharide position in the energy minimized structure in the GaMD and LiGaMD2 simulations. The percentages of frames where the RMSD value was above 3 Å are shown. Supplementary Table S2. Selected interactions between amino acids of AT and negatively charged groups in the pentasaccharide (distance cutoff was 5 Å). The pairs where the mutants show the largest differences from the WT are listed in the table.
