# Supplementary material for: Molecular Mechanisms of the Impaired Heparin Pentasaccharide Interactions in 10 Antithrombin Heparin Binding Site Mutants Revealed by Enhanced Sampling Molecular Dynamics
Source: Biomolecules. 2024 Jun 4;14(6):657. doi: 10.3390/biom14060657 (PMC11201378; doi:10.3390/biom14060657)
Supplement: Supplementary file 1 [file biomolecules-14-00657-s001.zip › biomolecules-2993289-supplementary.pdf]

Supporting Information

Molecular mechanisms of the impaired heparin pentasaccharide interactions in 10 antithrombin heparin binding site mutants revealed by enhanced sampling molecular dynamics

Gabor Balogh [1] and Zsuzsanna Bereczky [1]

[1] Division of Clinical Laboratory Science, Department of Laboratory Medicine, Faculty of Medicine, University of Debrecen; Debrecen, Hungary

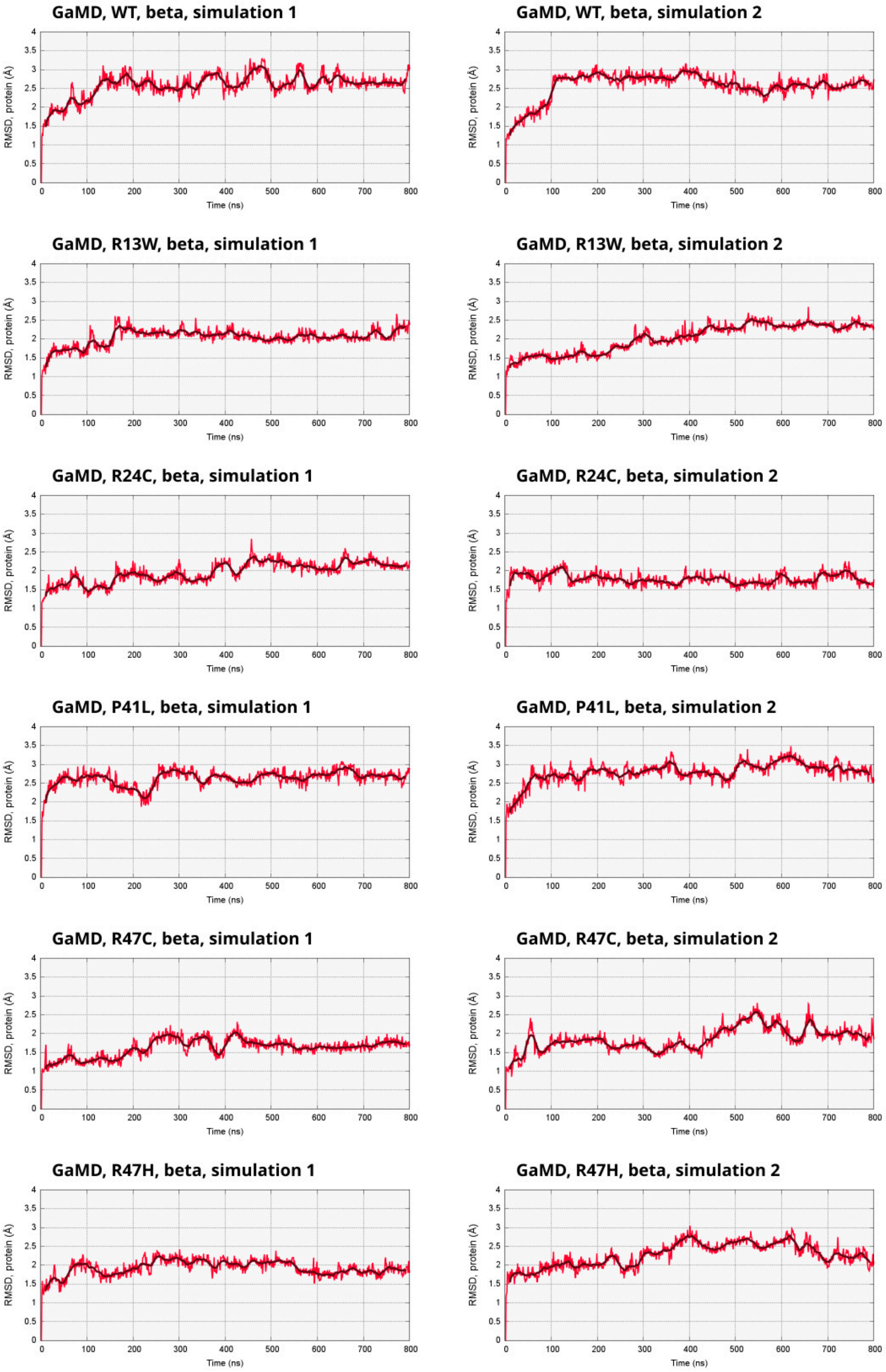

Supplementary Figure S1/A. RMSD of the alpha carbon atoms in the antithrombin protein, compared to the first frame of the simulations, in the GaMD simulations of beta-antithrombin. In this figure, results from simulations of the WT protein as well as the R13W, R24C, P41L, R47C and R47H mutants are shown.

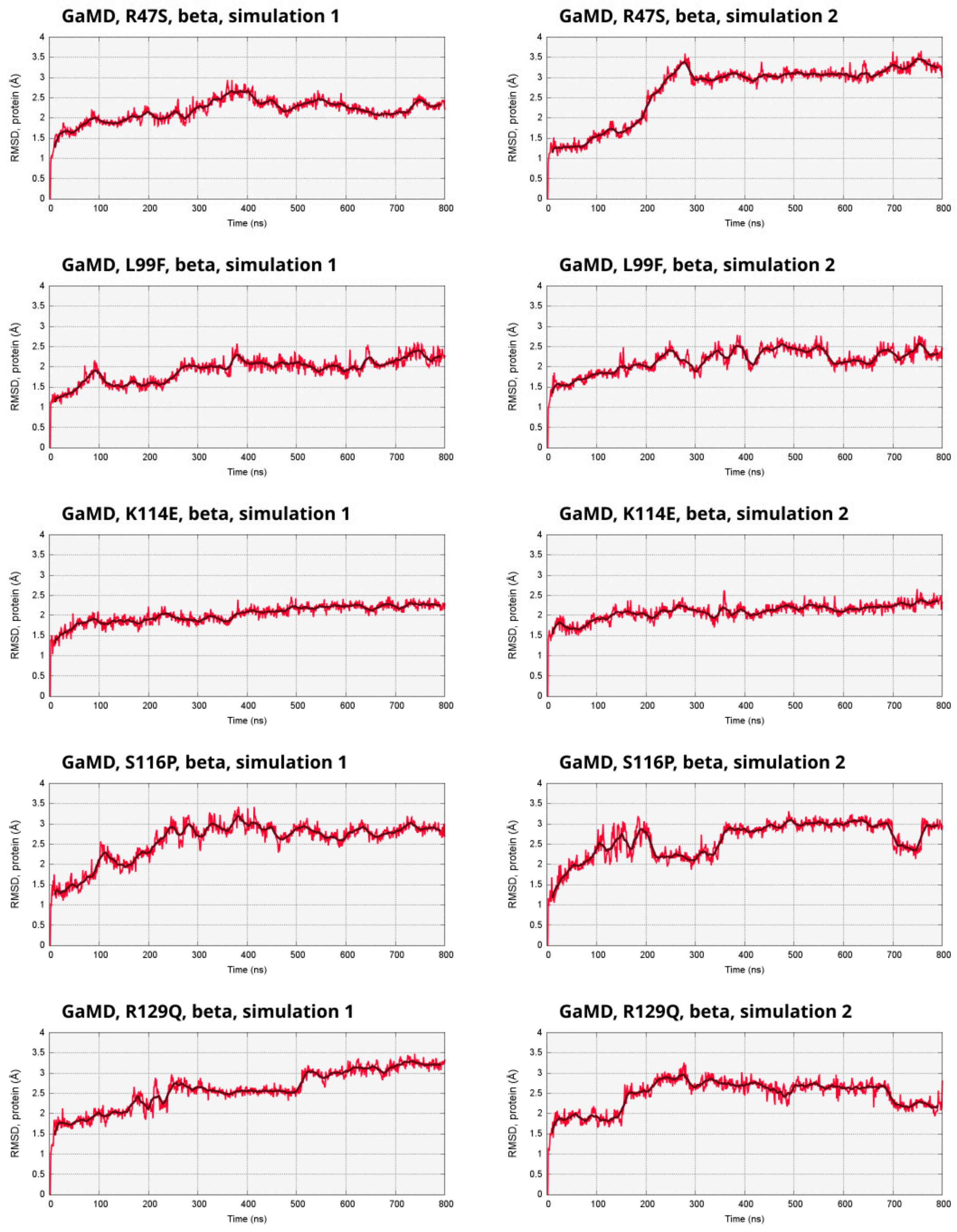

Supplementary Figure S1/B. RMSD of the alpha carbon atoms in the antithrombin protein, compared to the first frame of the simulations, in the GaMD simulations of beta-antithrombin. In this figure, results from simulations of the R47S, L99F, K114E, S116P and R129Q mutants are shown.

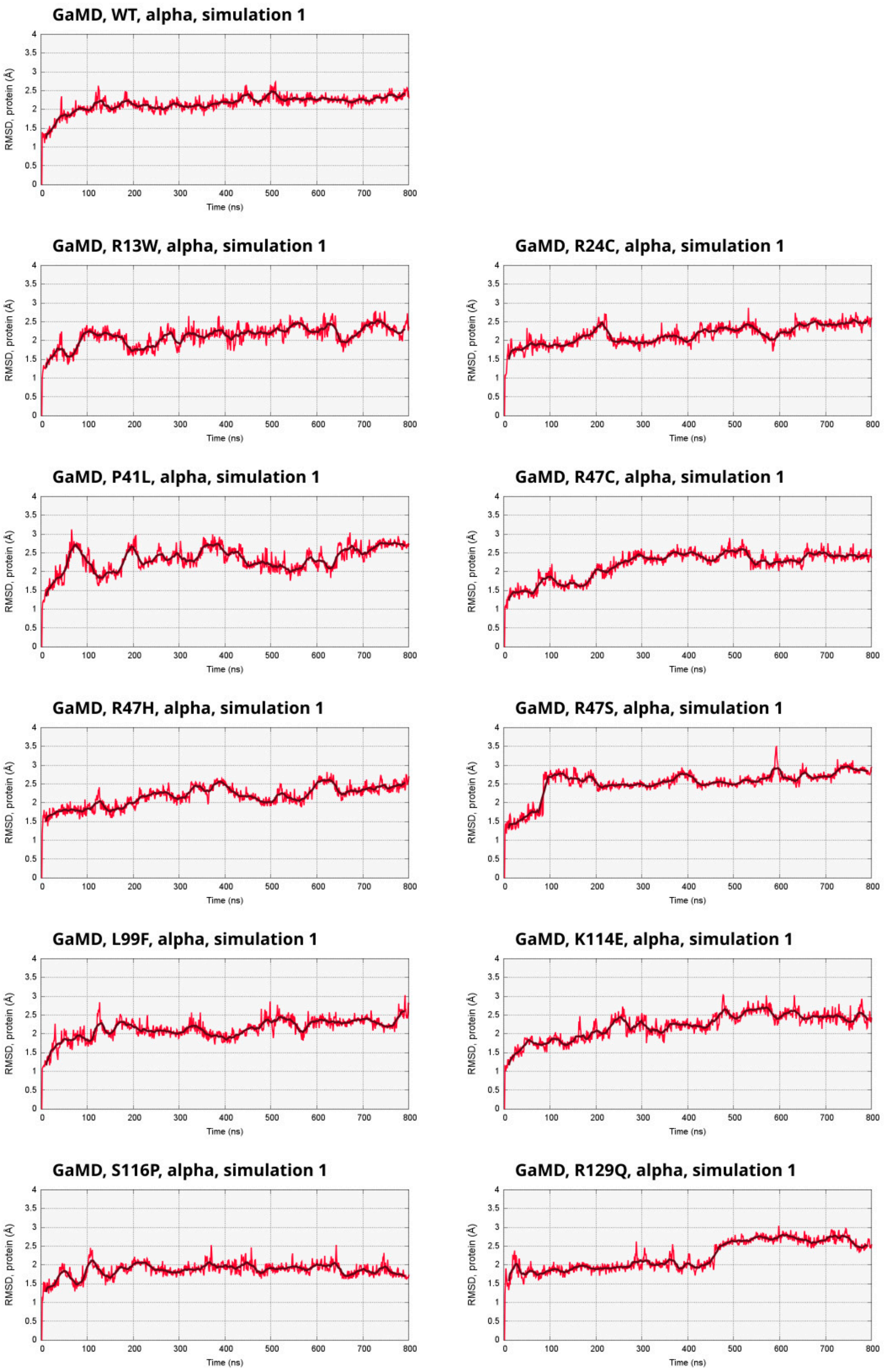

Supplementary Figure S2. RMSD of the alpha carbon atoms in the antithrombin protein, compared to the first frame of the simulations, in the GaMD simulations of alpha-antithrombin.

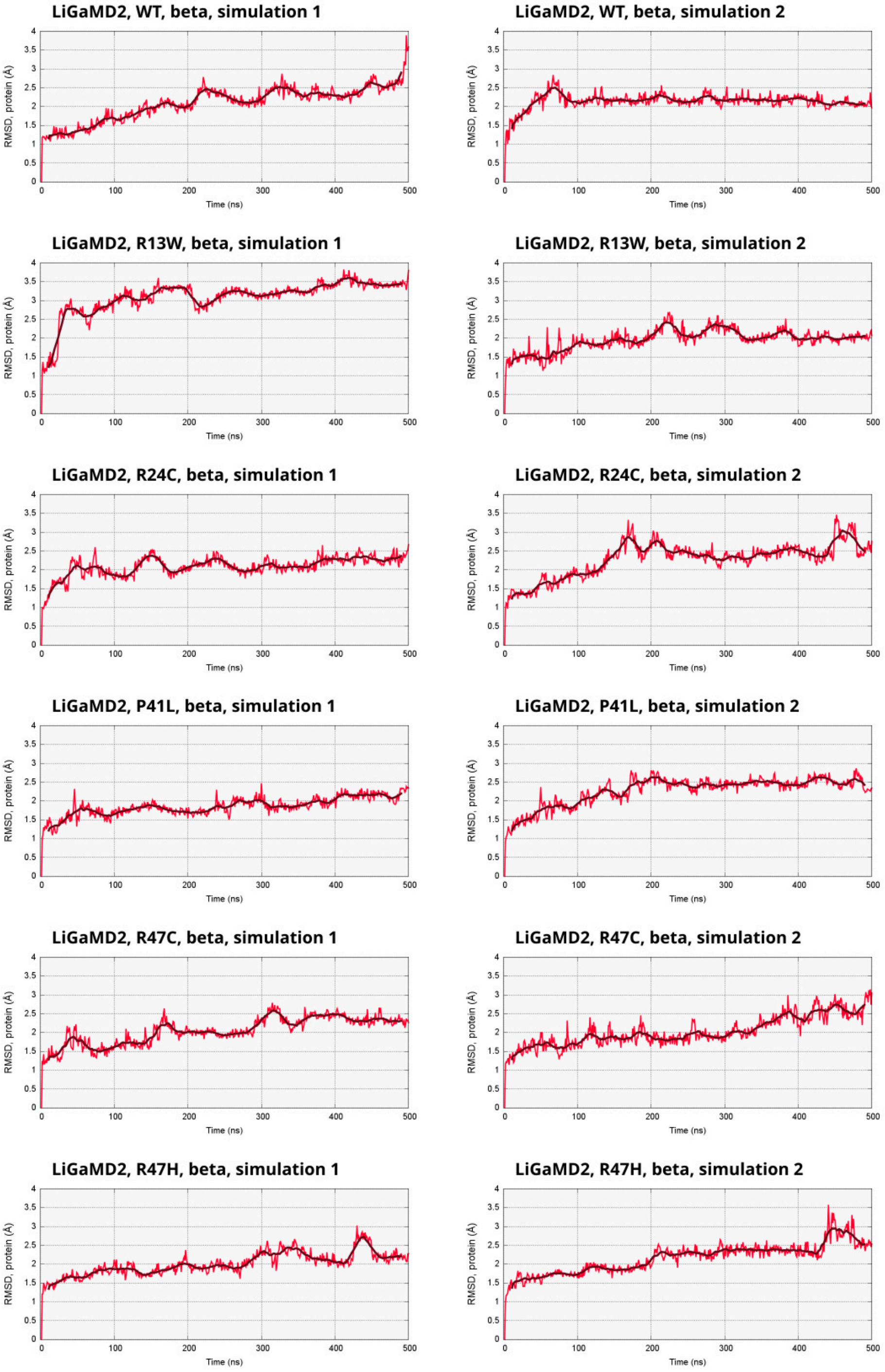

Supplementary Figure S3/A. RMSD of the alpha carbon atoms in the antithrombin protein, compared to the first frame of the simulations, in the LiGaMD2 simulations of beta-antithrombin. In this figure, results from simulations of the WT protein as well as the R13W, R24C, P41L, R47C and R47H mutants are shown.

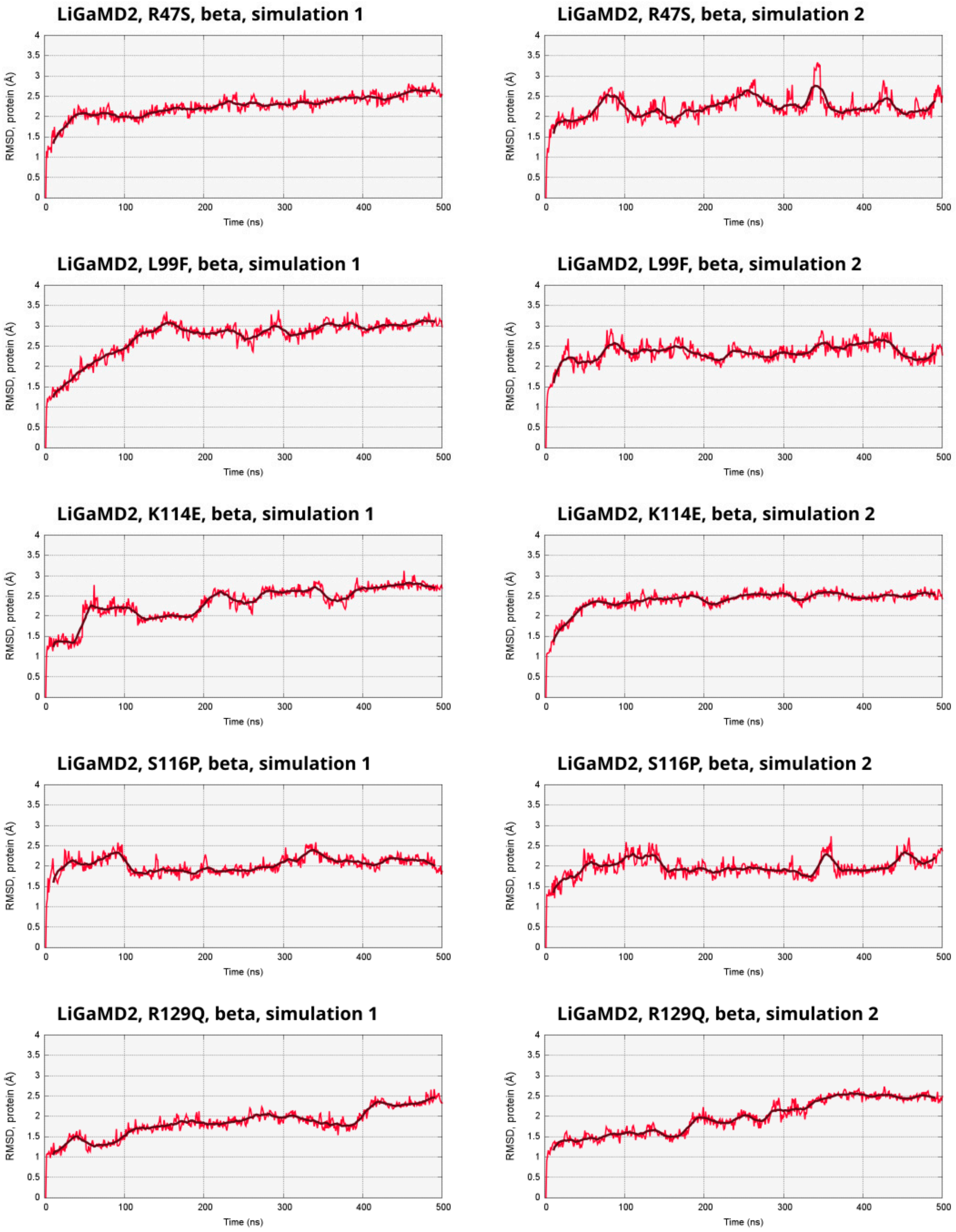

Supplementary Figure S3/B. RMSD of the alpha carbon atoms in the antithrombin protein, compared to the first frame of the simulations, in the LiGaMD2 simulations of beta-antithrombin. In this figure, results from simulations of the R47S, L99F, K114E, S116P and R129Q mutants are shown.

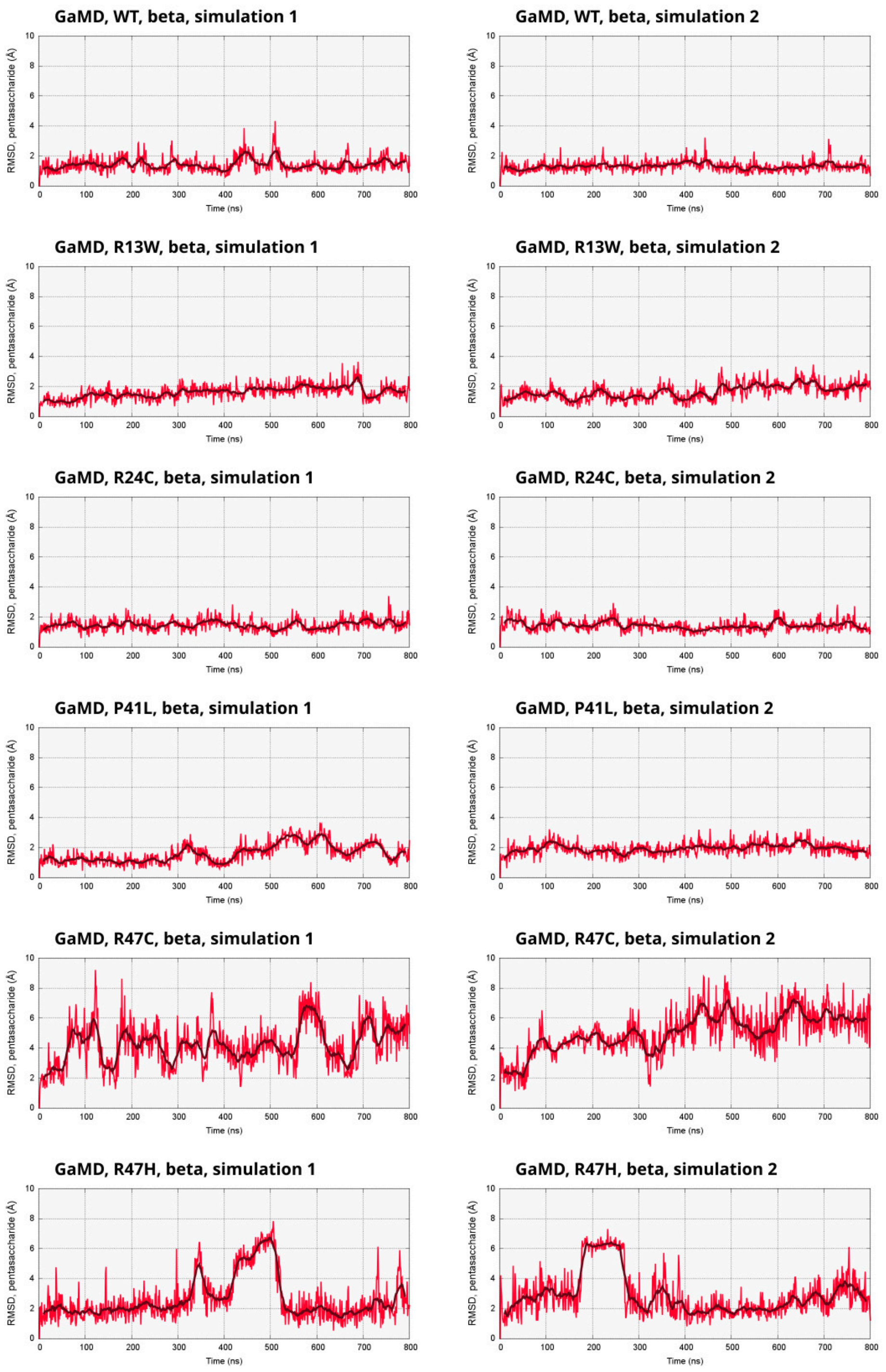

Supplementary Figure S4/A. The RMSD of the pentasaccharide ligand compared to its position in the energy minimized structure, as a function of time, in the GaMD simulations of beta AT. In this figure, results from simulations of the WT protein as well as the R13W, R24C, P41L, R47C and R47H mutants are shown.

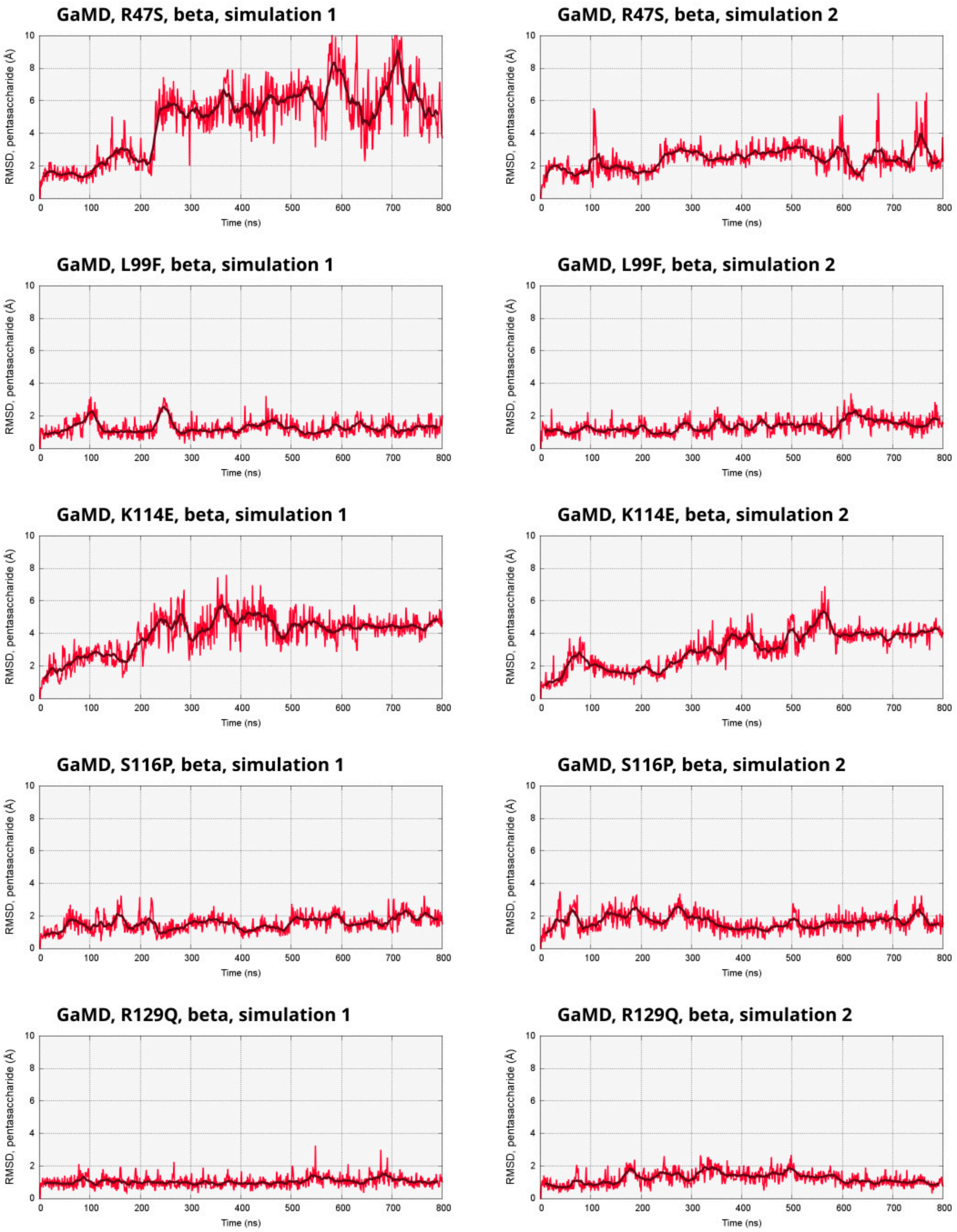

Supplementary Figure S4/B. The RMSD of the pentasaccharide ligand compared to its position in the energy minimized structure, as a function of time, in the GaMD simulations of beta AT. In this figure, results from simulations of the R47S, L99F, K114E, S116P and R129Q mutants are shown.

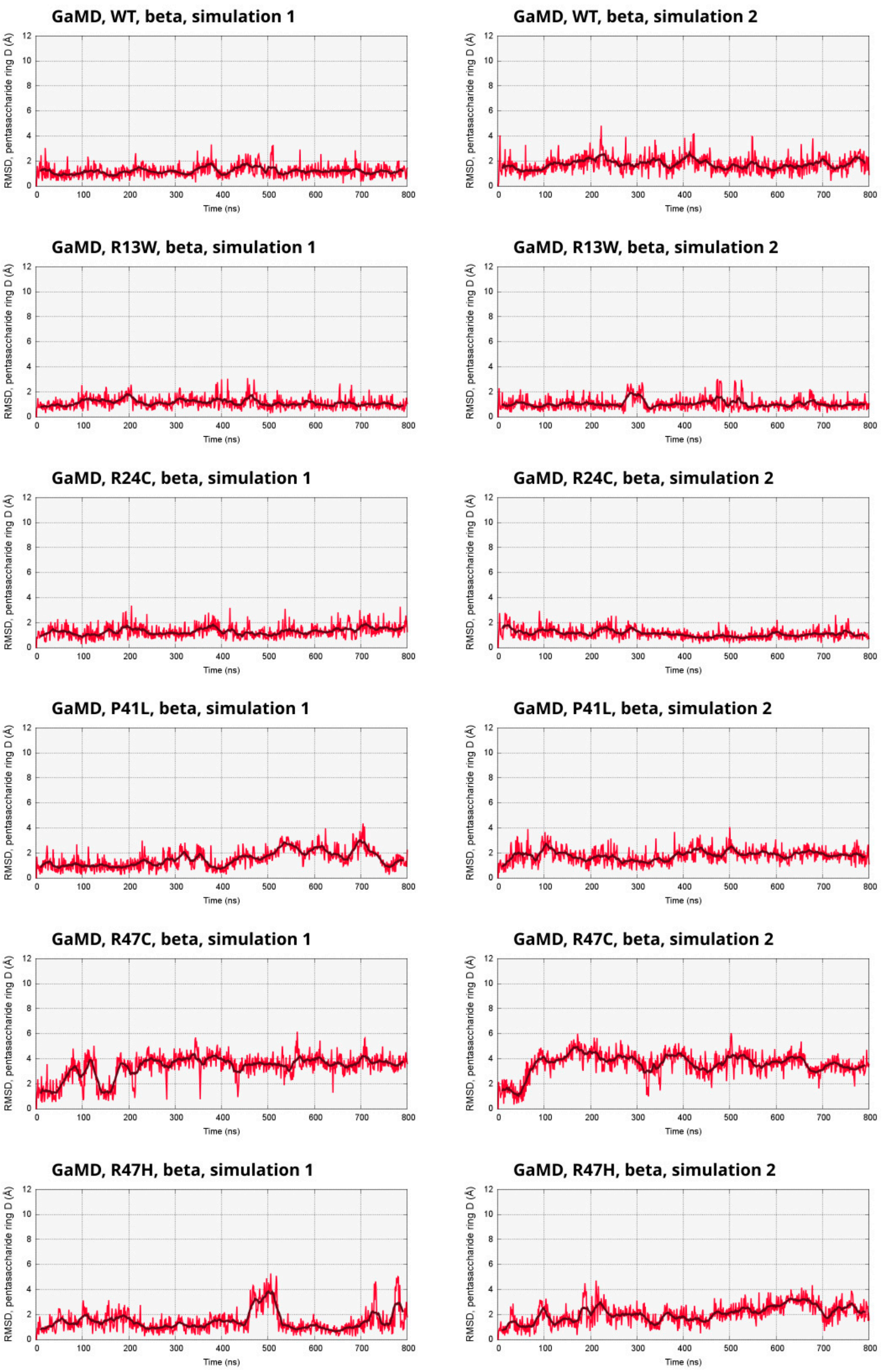

Supplementary Figure S5/A. The RMSD of ring D in the pentasaccharide ligand compared to its position in the energy minimized structure, as a function of time, in the GaMD simulations of beta AT. In this figure, results from simulations of the WT protein as well as the R13W, R24C, P41L, R47C and R47H mutants are shown.

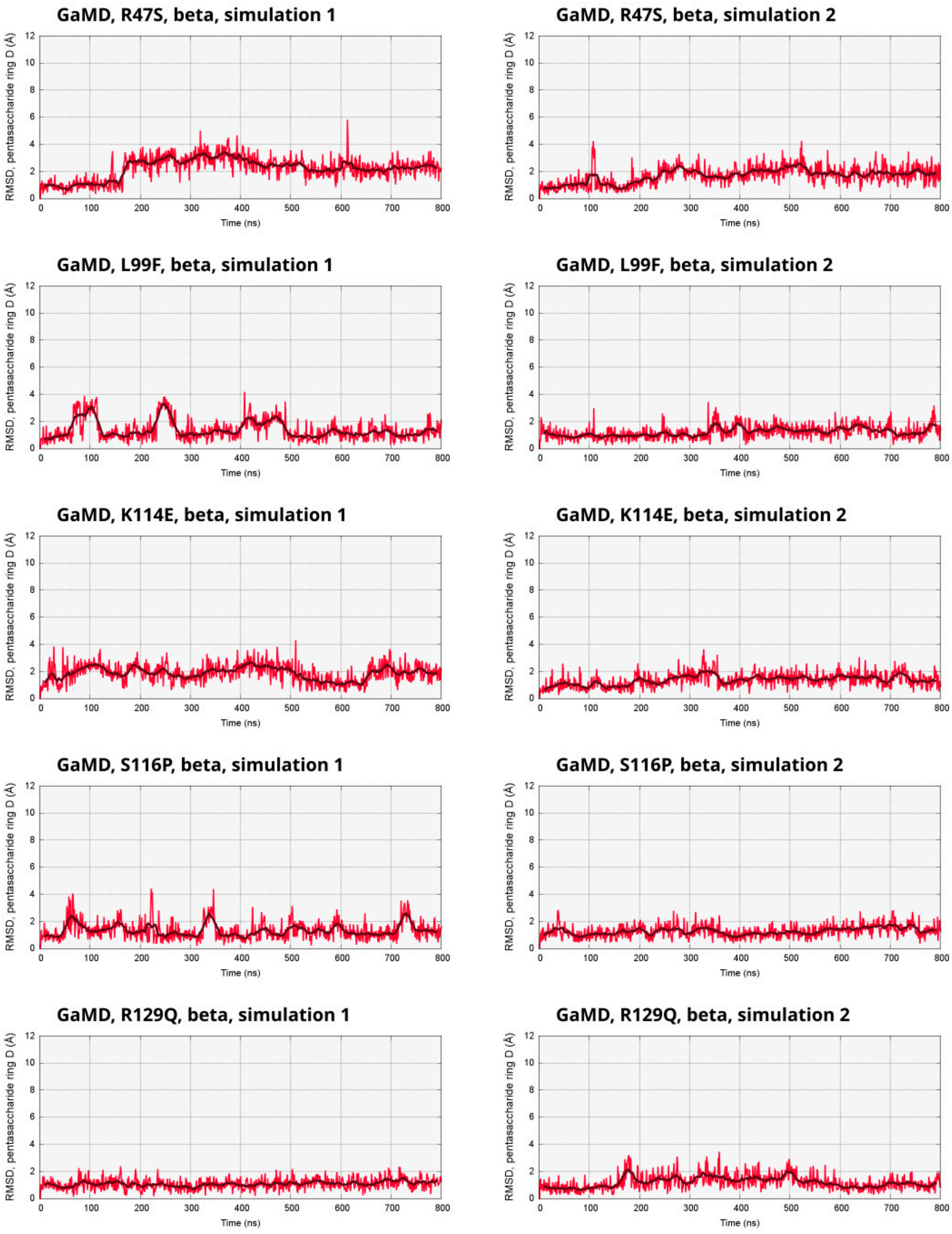

Supplementary Figure S5/B. The RMSD of ring D the pentasaccharide ligand compared to its position in the energy minimized structure, as a function of time, in the GaMD simulations of beta AT. In this figure, results from simulations of the R47S, L99F, K114E, S116P and R129Q mutants are shown.

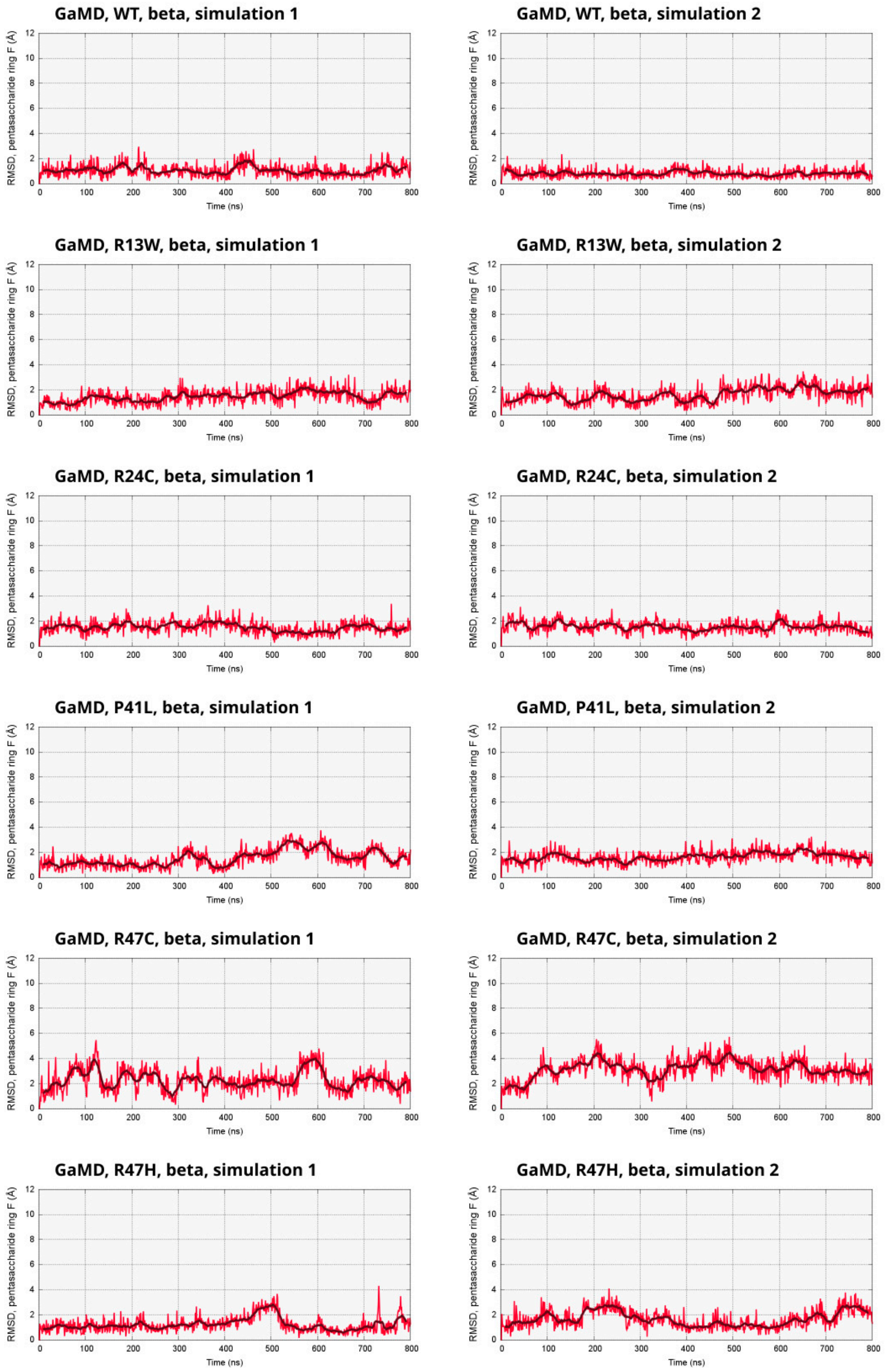

Supplementary Figure S6/A. The RMSD of ring F in the pentasaccharide ligand compared to its position in the energy minimized structure, as a function of time, in the GaMD simulations of beta AT. In this figure, results from simulations of the WT protein as well as the R13W, R24C, P41L, R47C and R47H mutants are shown.

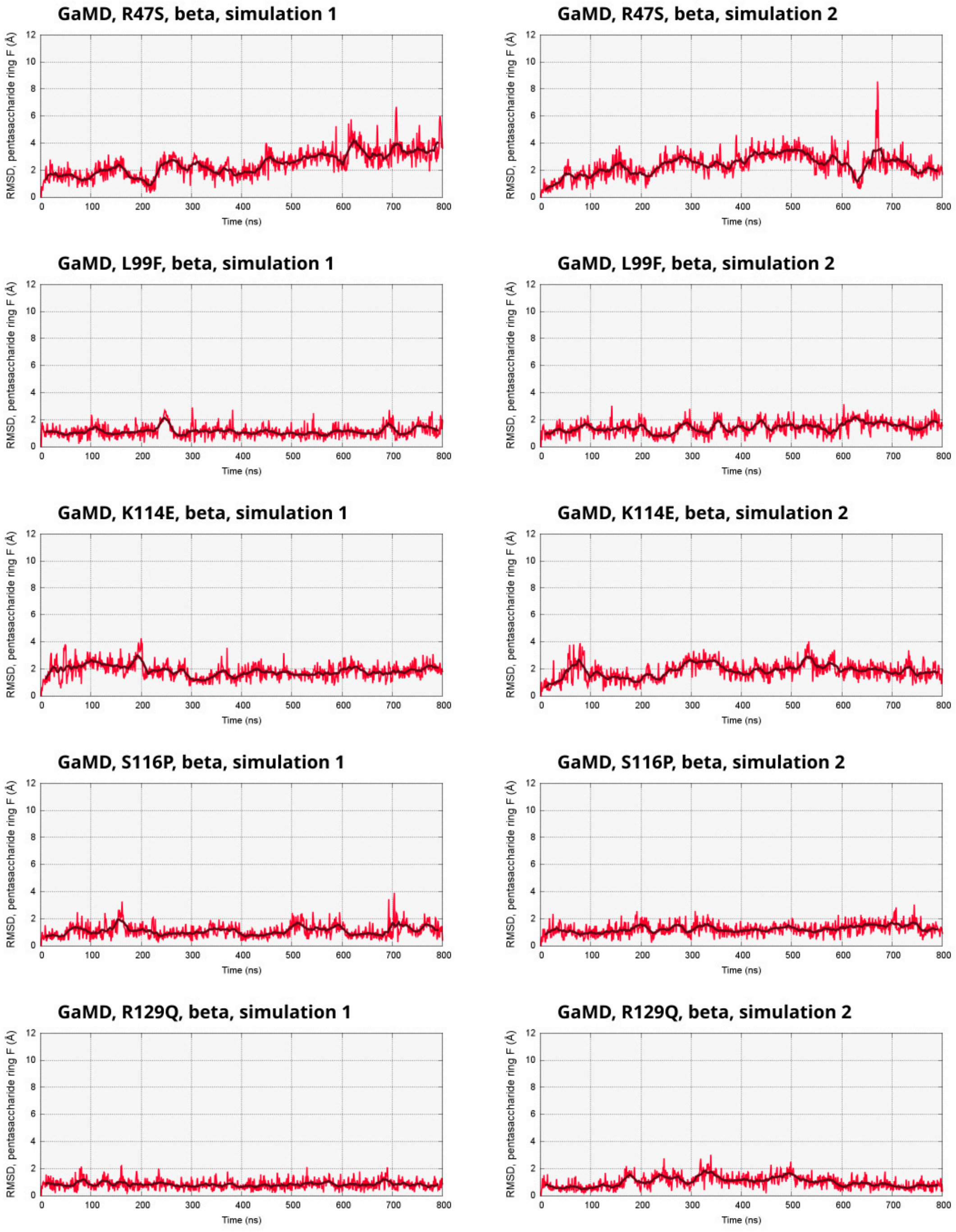

Supplementary Figure S6/B. The RMSD of ring F the pentasaccharide ligand compared to its position in the energy minimized structure, as a function of time, in the GaMD simulations of beta AT. In this figure, results from simulations of the R47S, L99F, K114E, S116P and R129Q mutants are shown.

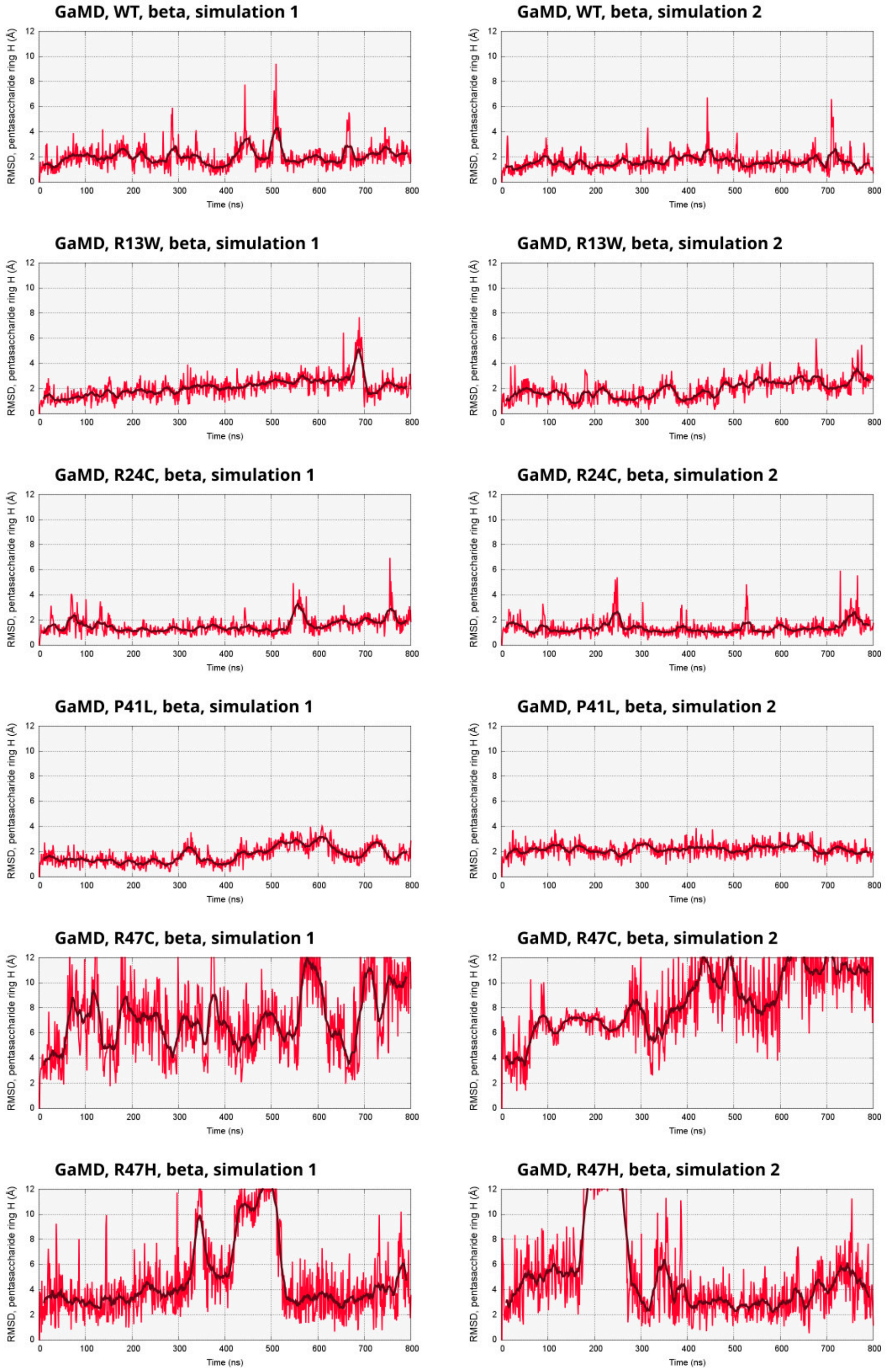

Supplementary Figure S7/A. The RMSD of ring H in the pentasaccharide ligand compared to its position in the energy minimized structure, as a function of time, in the GaMD simulations of beta AT. In this figure, results from simulations of the WT protein as well as the R13W, R24C, P41L, R47C and R47H mutants are shown.

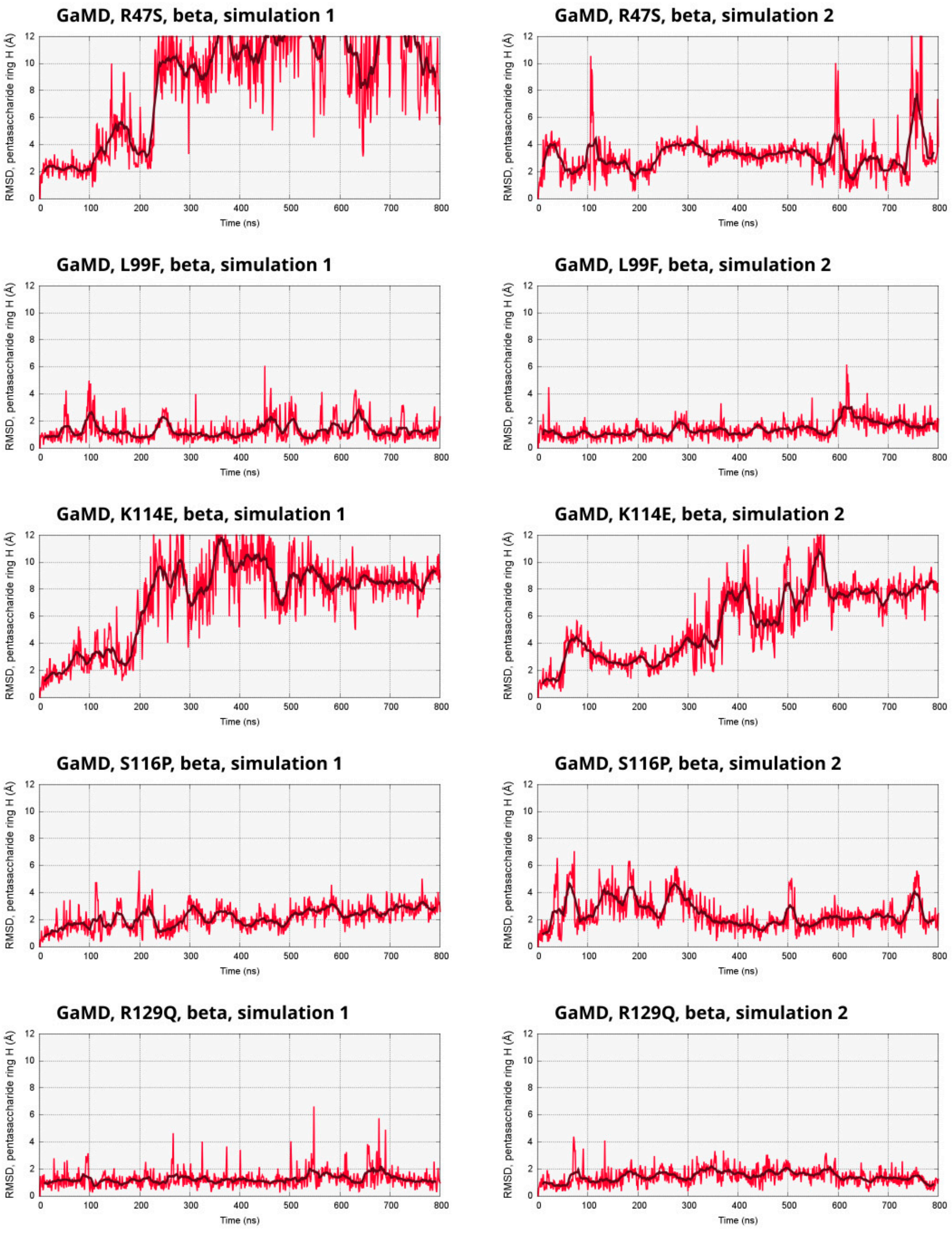

Supplementary Figure S7/B. The RMSD of ring H the pentasaccharide ligand compared to its position in the energy minimized structure, as a function of time, in the GaMD simulations of beta AT. In this figure, results from simulations of the R47S, L99F, K114E, S116P and R129Q mutants are shown.

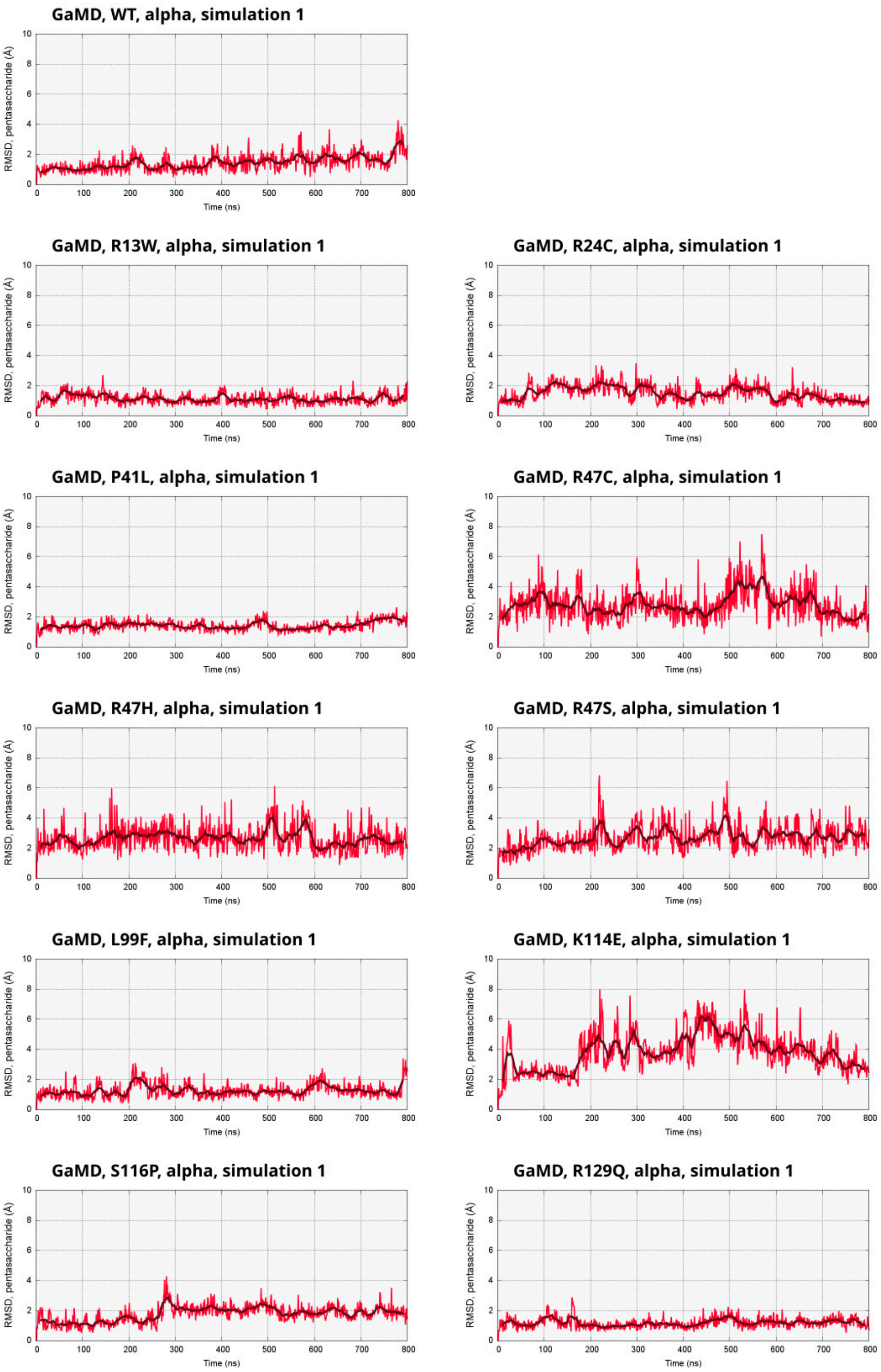

Supplementary Figure S8. The RMSD of the pentasaccharide ligand compared to its position in the energy minimized structure, as a function of time, in the GaMD simulations of alpha AT.

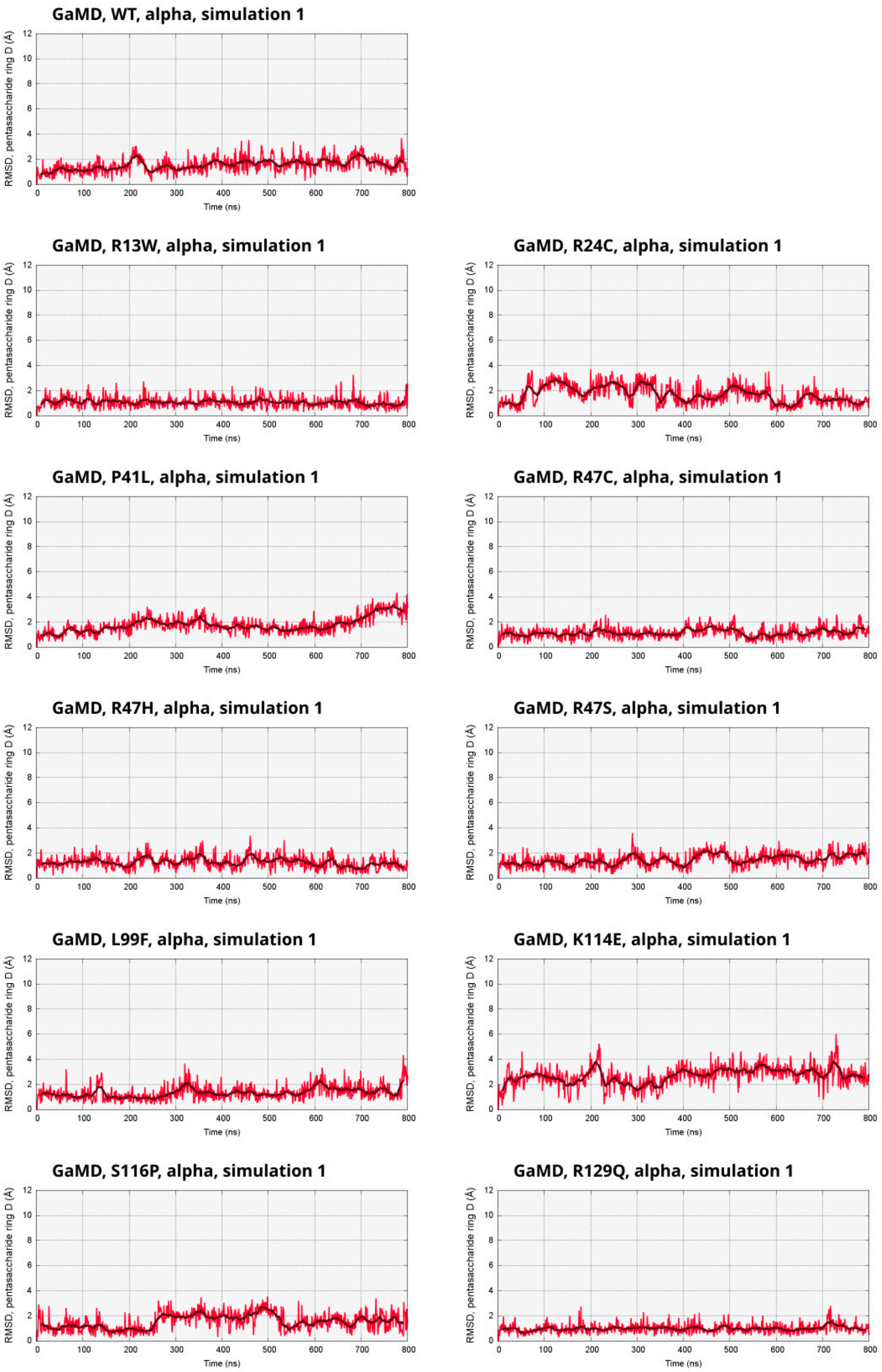

Supplementary Figure S9. The RMSD of ring D in the pentasaccharide ligand compared to its position in the energy minimized structure, as a function of time, in the GaMD simulations of alpha AT.

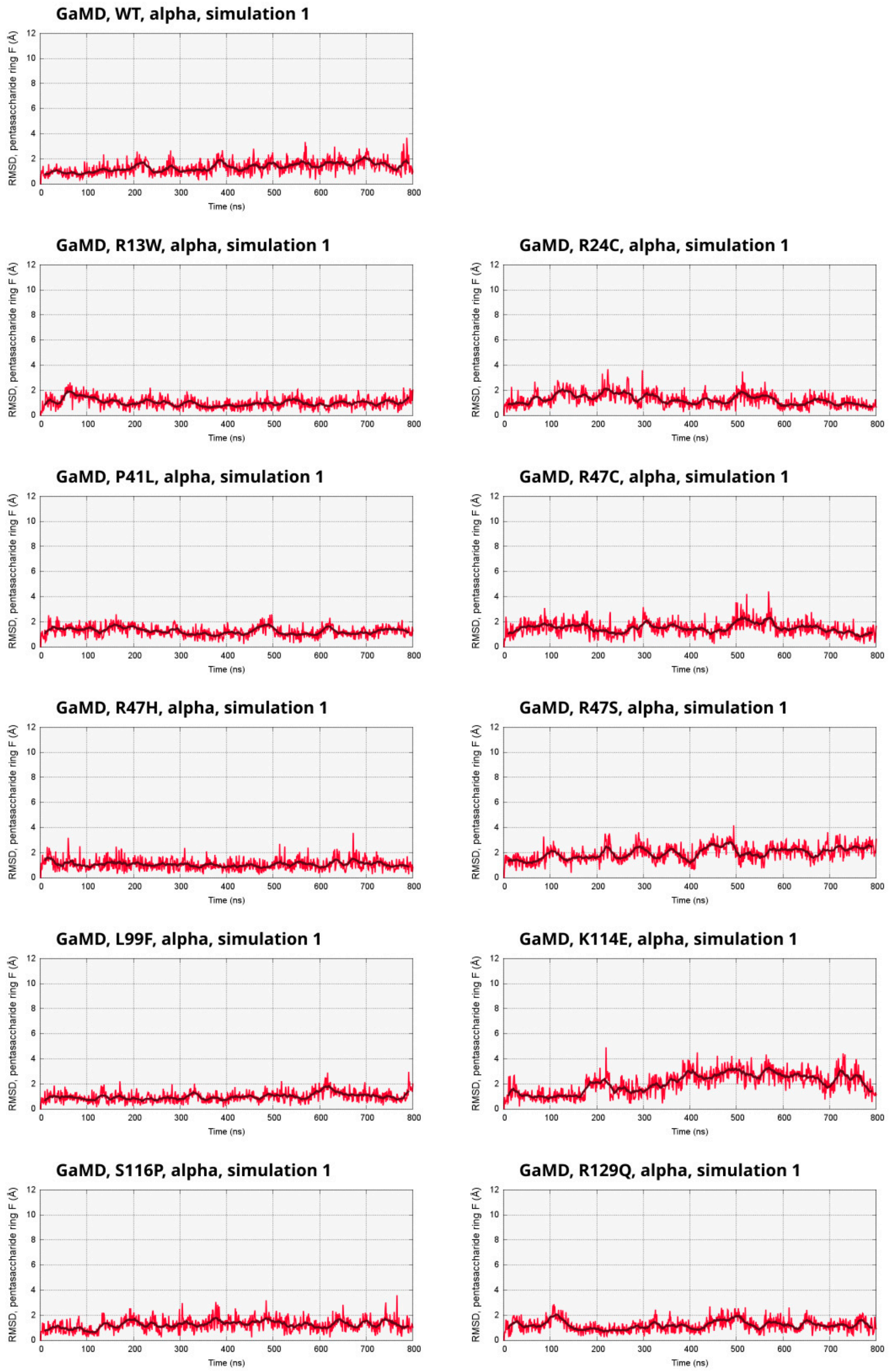

Supplementary Figure S10. The RMSD of ring F in the pentasaccharide ligand compared to its position in the energy minimized structure, as a function of time, in the GaMD simulations of alpha AT.

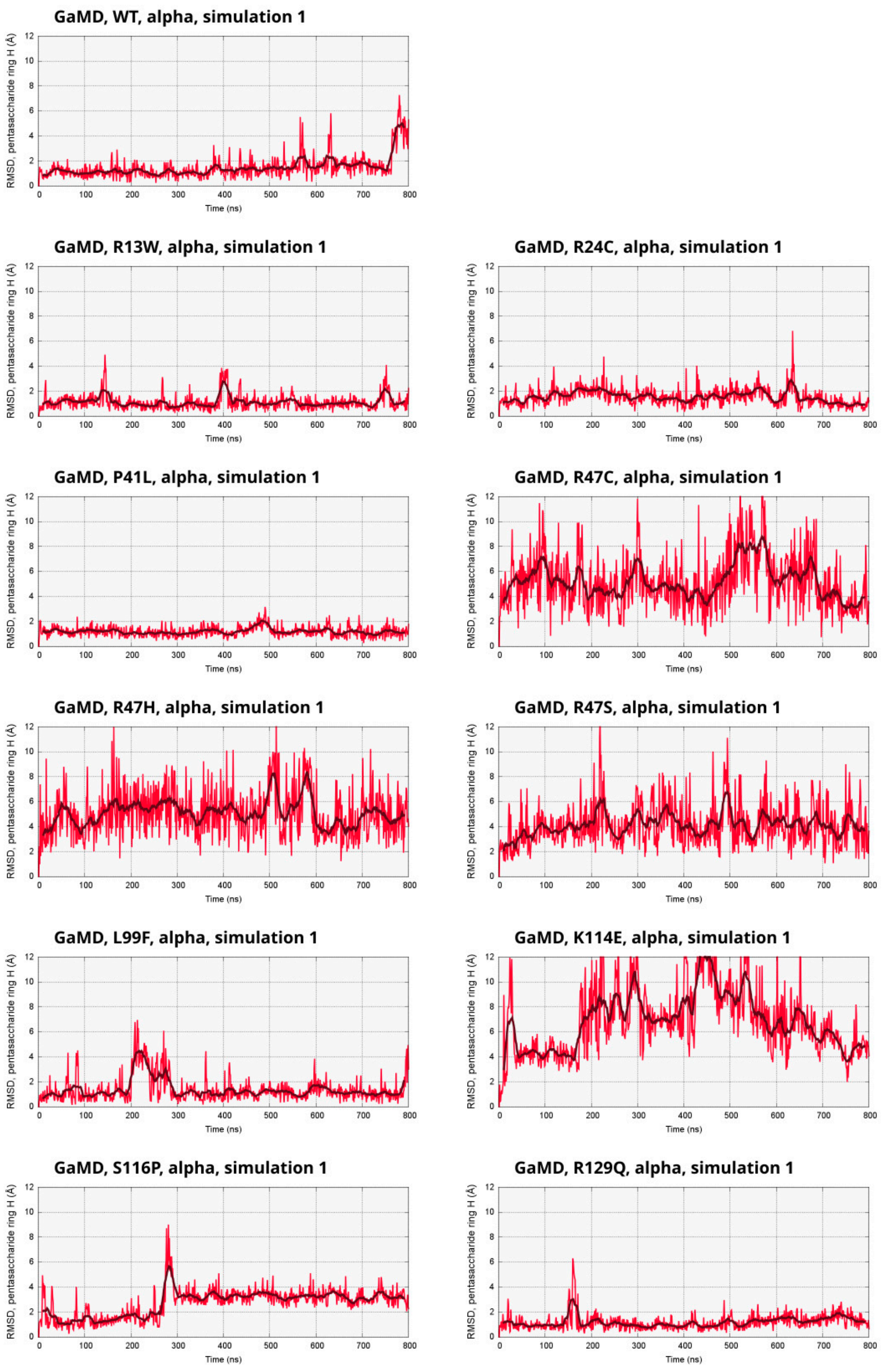

Supplementary Figure S11 The RMSD of ring H in the pentasaccharide ligand compared to its position in the energy minimized structure, as a function of time, in the GaMD simulations of alpha AT.

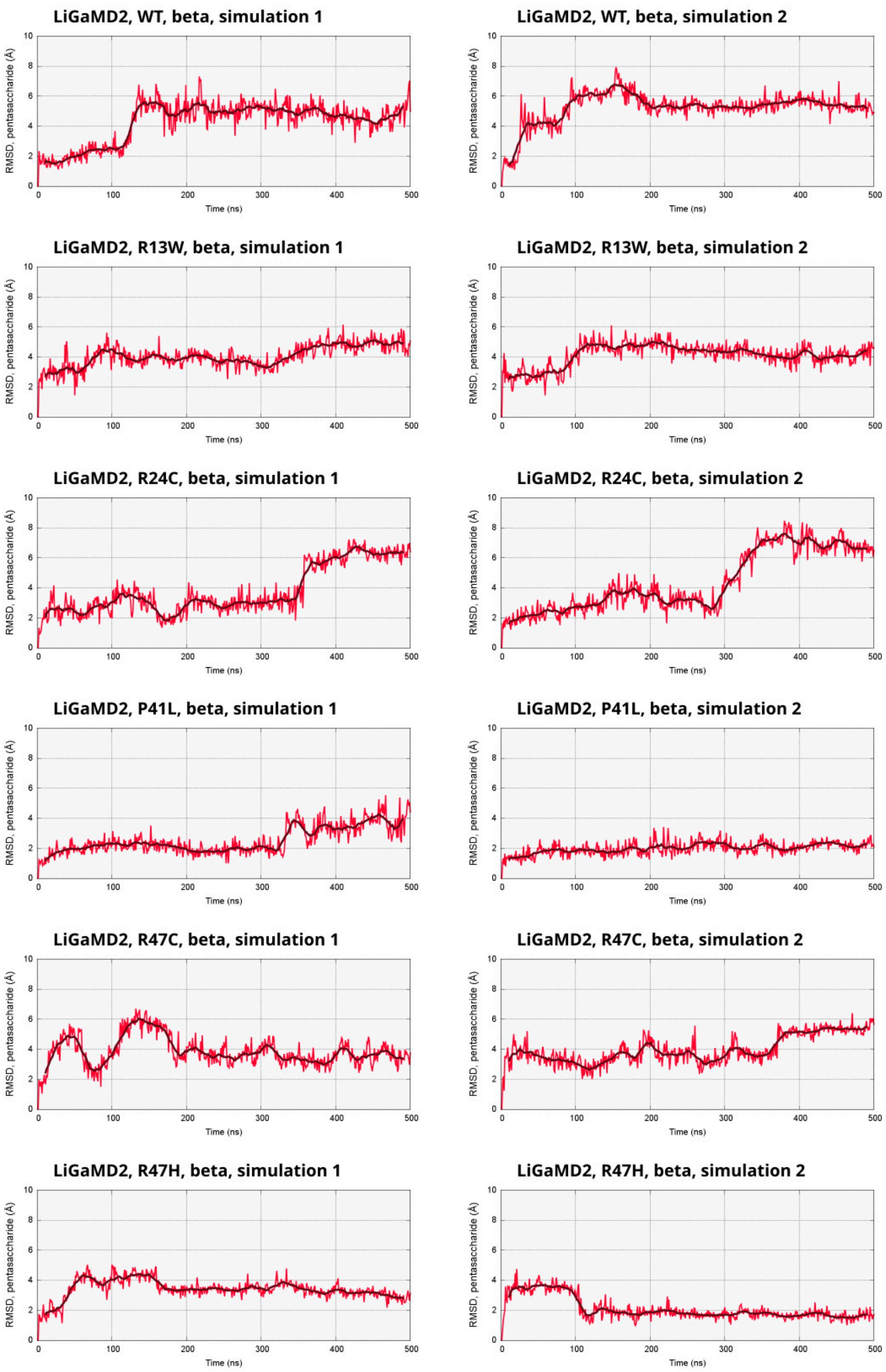

Supplementary Figure S12/A. The RMSD of the pentasaccharide ligand compared to its position in the energy minimized structure, as a function of time, in the LiGaMD2 simulations of beta AT. In this figure, results from simulations of the WT protein as well as the R13W, R24C, P41L, R47C and R47H mutants are shown.

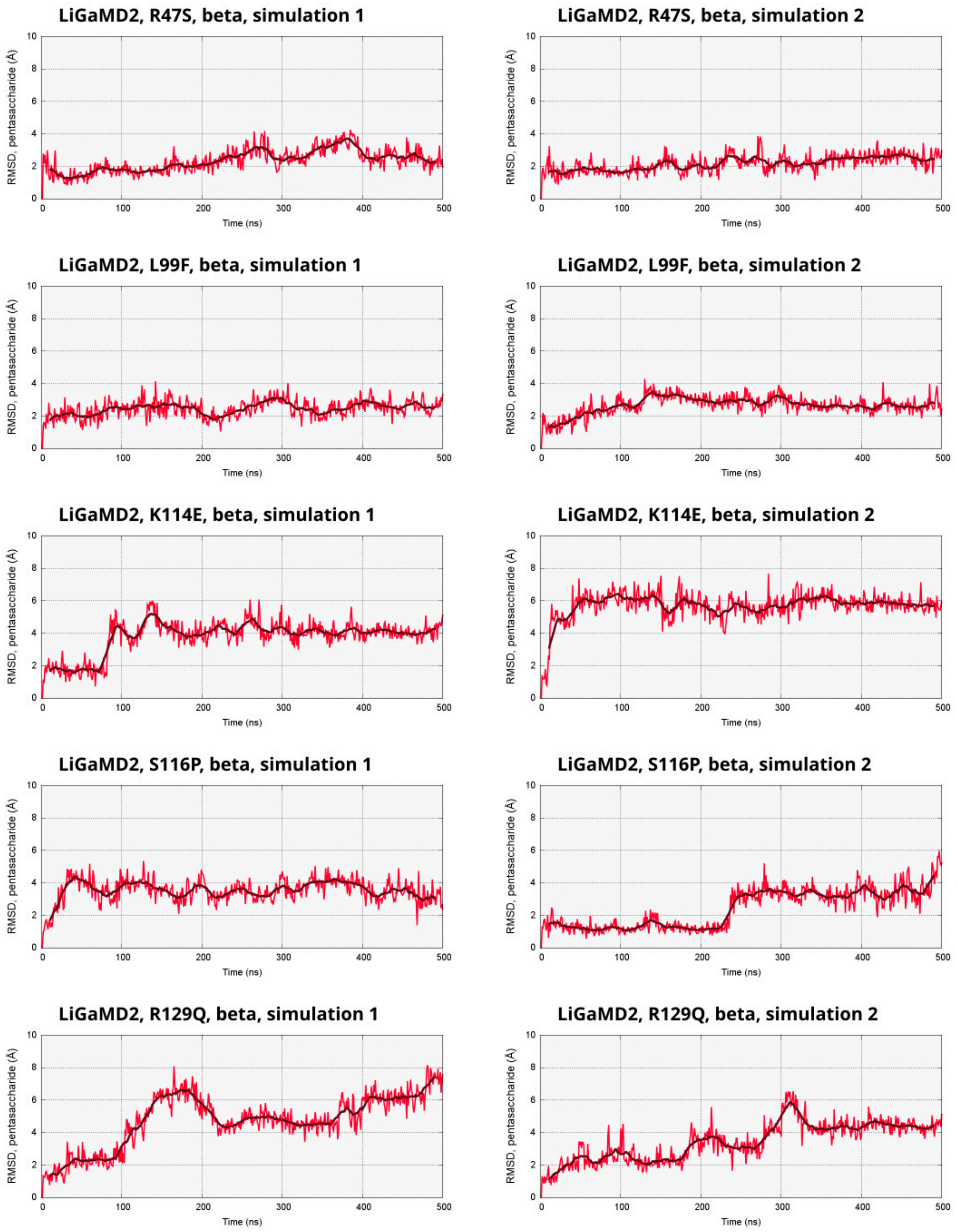

Supplementary Figure S12/B. The RMSD of the pentasaccharide ligand compared to its position in the energy minimized structure, as a function of time, in the LiGaMD2 simulations of beta AT. In this figure, results from simulations of the R47S, L99F, K114E, S116P and R129Q mutants are shown.

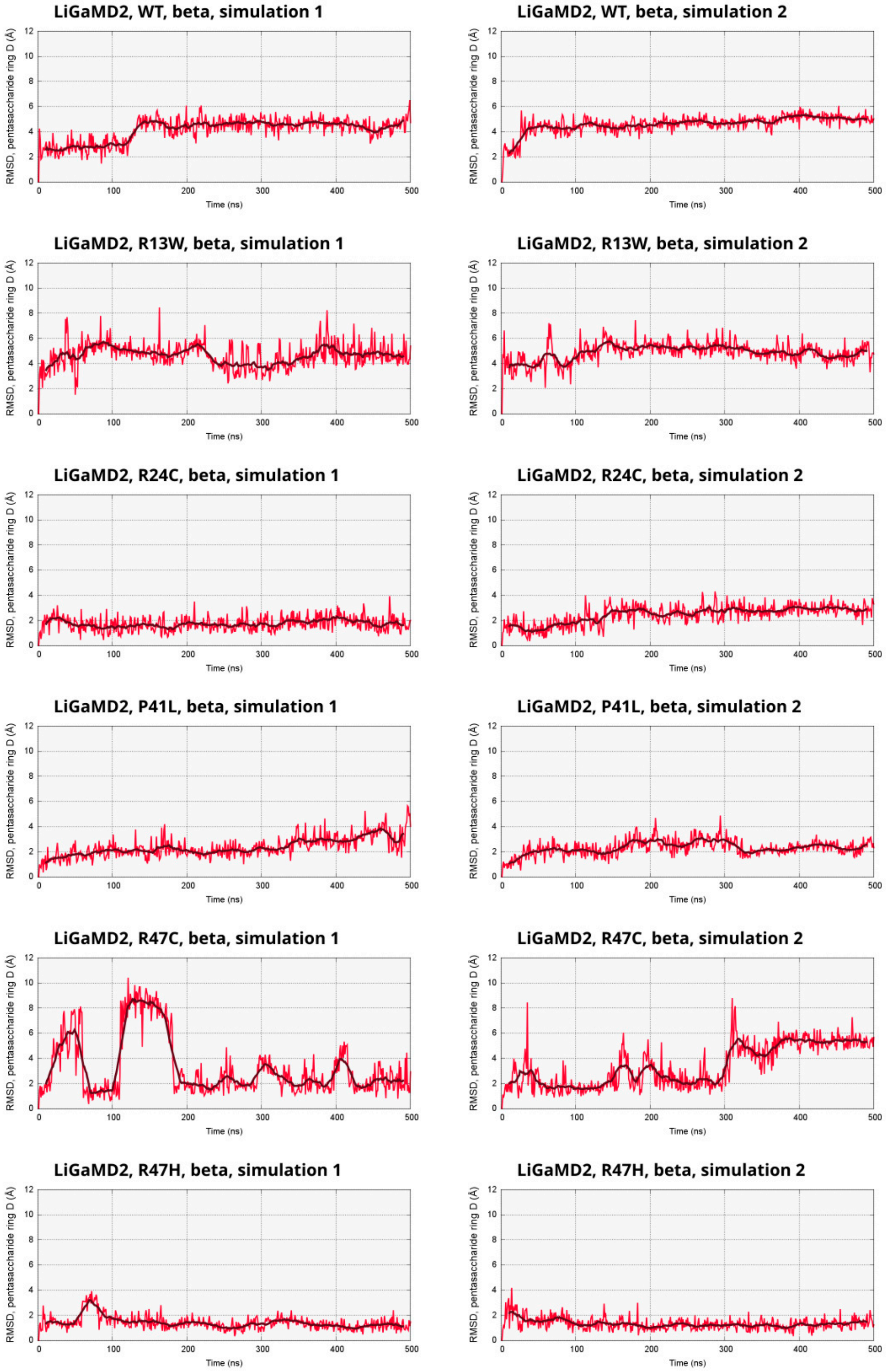

Supplementary Figure S13/A. The RMSD of ring D in the pentasaccharide ligand compared to its position in the energy minimized structure, as a function of time, in the LiGaMD2 simulations of beta AT. In this figure, results from simulations of the WT protein as well as the R13W, R24C, P41L, R47C and R47H mutants are shown.

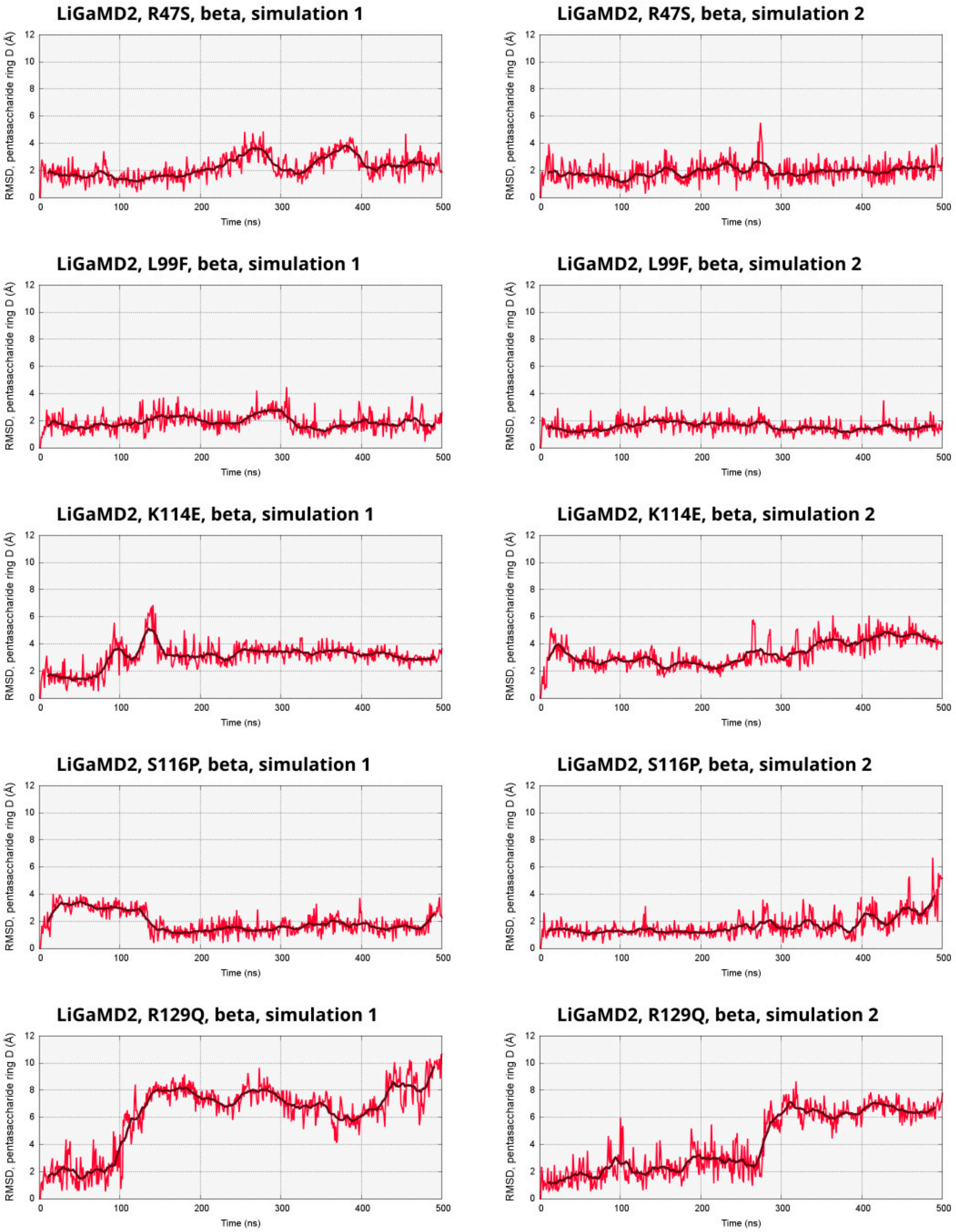

Supplementary Figure S13/B. The RMSD of ring D the pentasaccharide ligand compared to its position in the energy minimized structure, as a function of time, in the LiGaMD2 simulations of beta AT. In this figure, results from simulations of the R47S, L99F, K114E, S116P and R129Q mutants are shown.

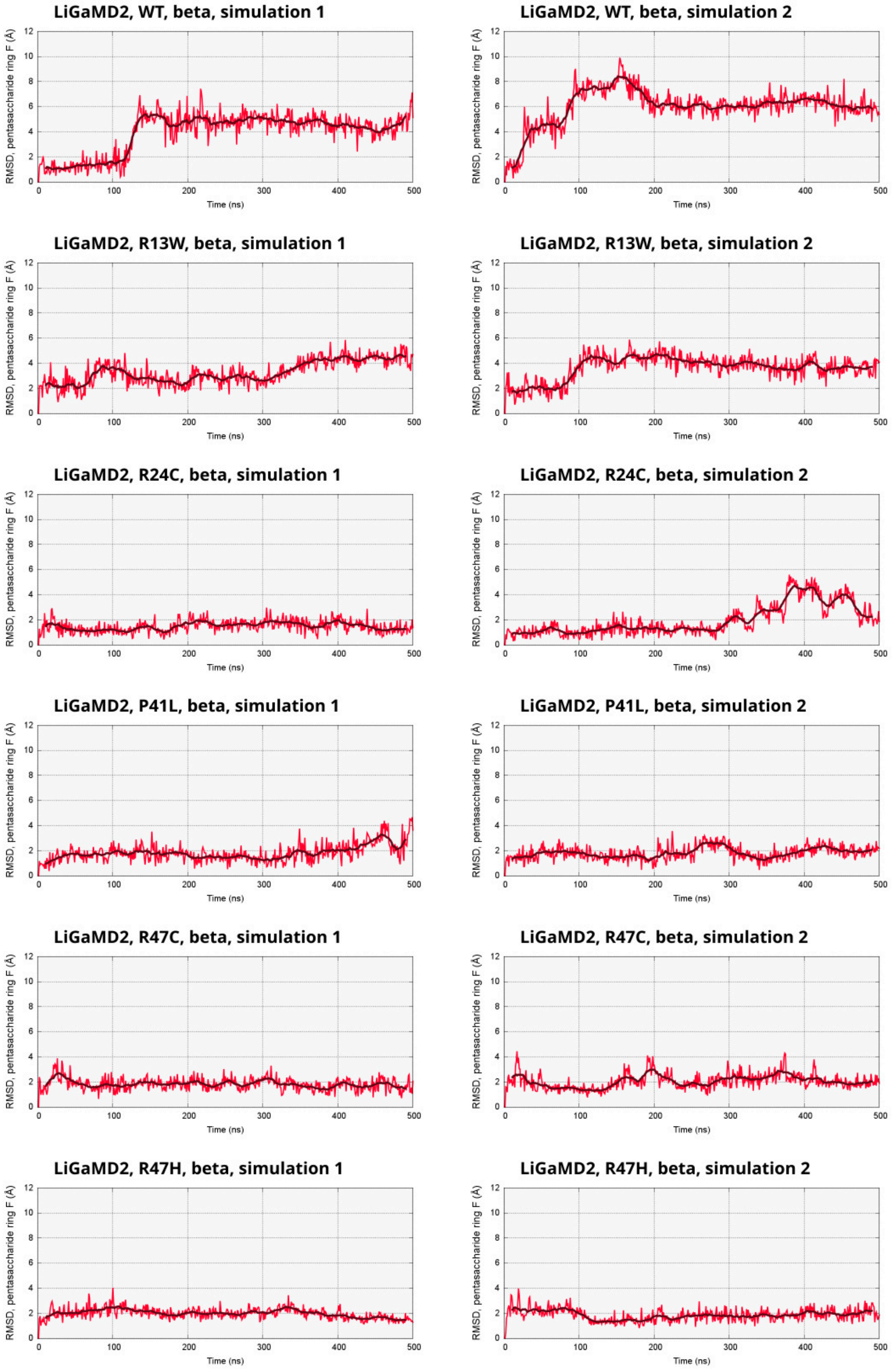

Supplementary Figure S14/A. The RMSD of ring F in the pentasaccharide ligand compared to its position in the energy minimized structure, as a function of time, in the LiGaMD2 simulations of beta AT. In this figure, results from simulations of the WT protein as well as the R13W, R24C, P41L, R47C and R47H mutants are shown.

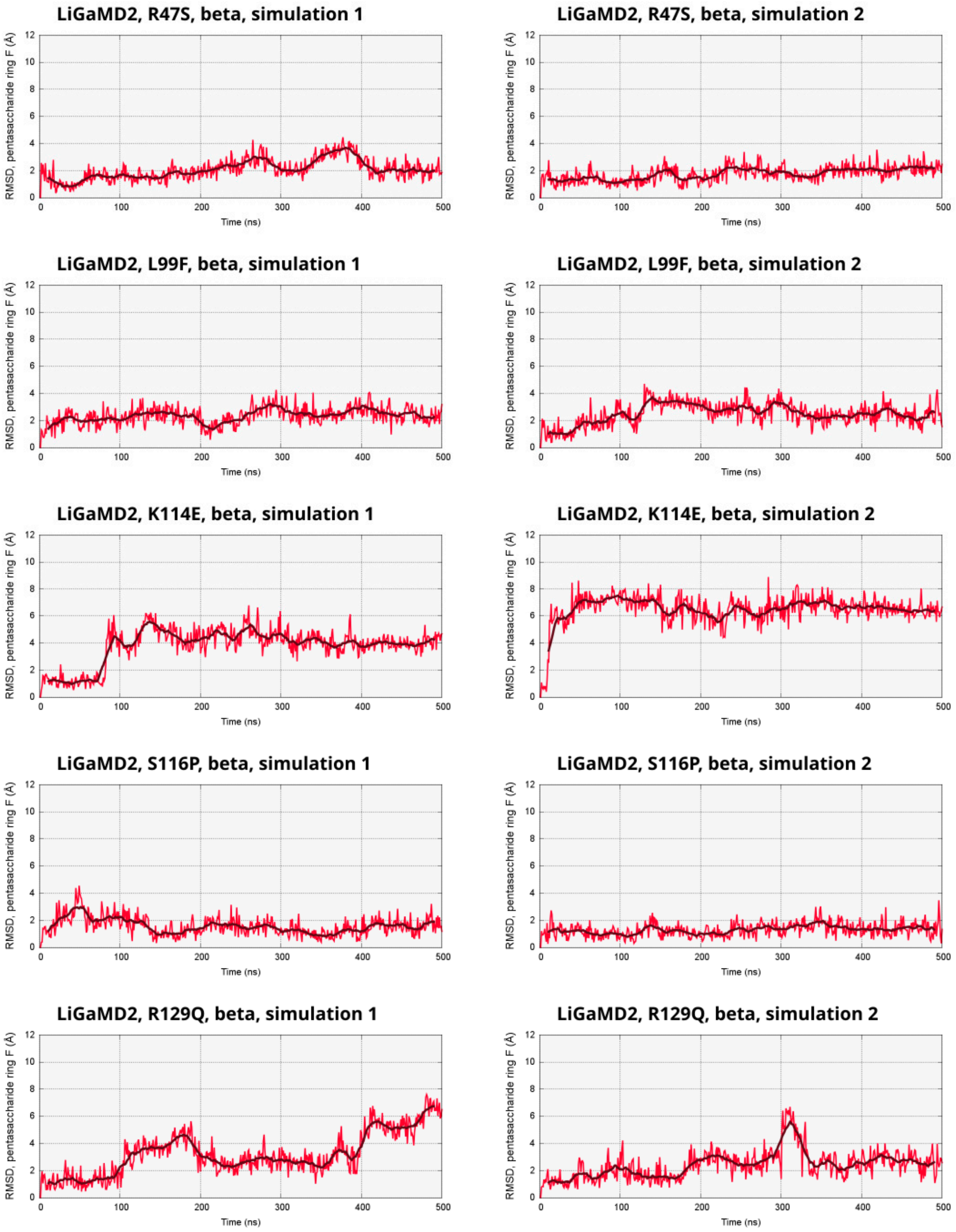

Supplementary Figure S14/B. The RMSD of ring F the pentasaccharide ligand compared to its position in the energy minimized structure, as a function of time, in the LiGaMD2 simulations of beta AT. In this figure, results from simulations of the R47S, L99F, K114E, S116P and R129Q mutants are shown.

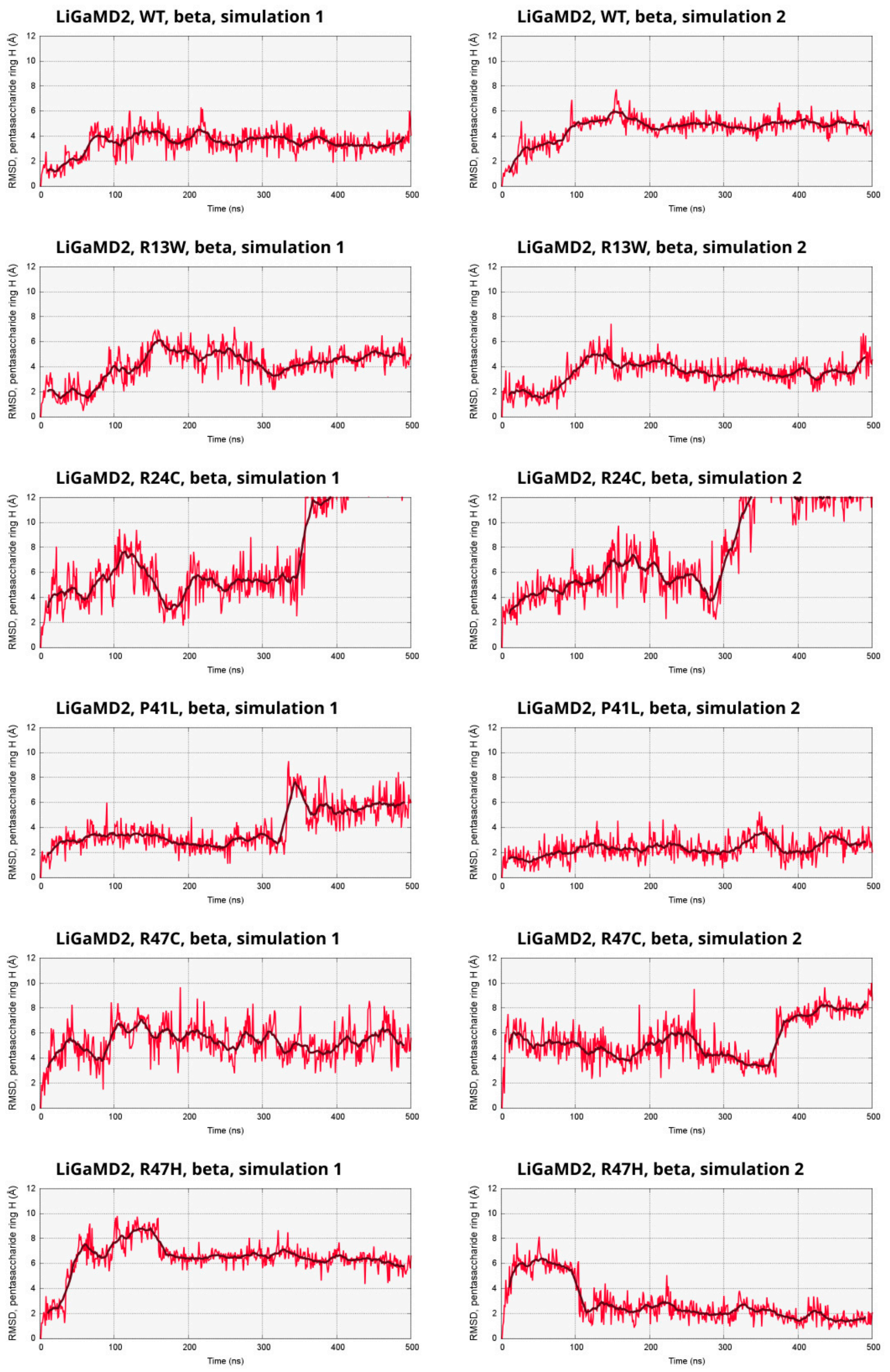

Supplementary Figure S15/A. The RMSD of ring H in the pentasaccharide ligand compared to its position in the energy minimized structure, as a function of time, in the LiGaMD2 simulations of beta AT. In this figure, results from simulations of the WT protein as well as the R13W, R24C, P41L, R47C and R47H mutants are shown.

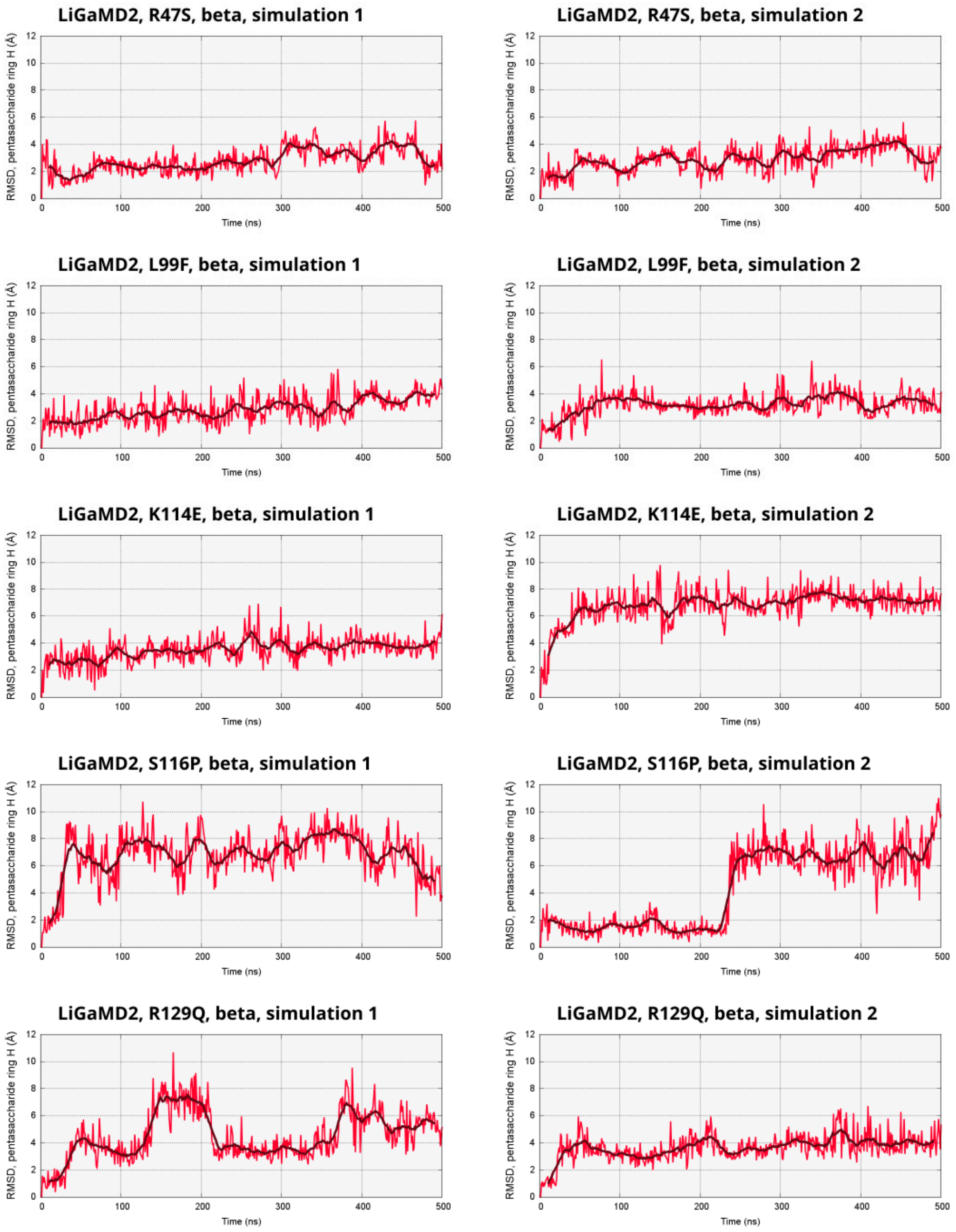

Supplementary Figure S15/B. The RMSD of ring H the pentasaccharide ligand compared to its position in the energy minimized structure, as a function of time, in the LiGaMD2 simulations of beta AT. In this figure, results from simulations of the R47S, L99F, K114E, S116P and R129Q mutants are shown.

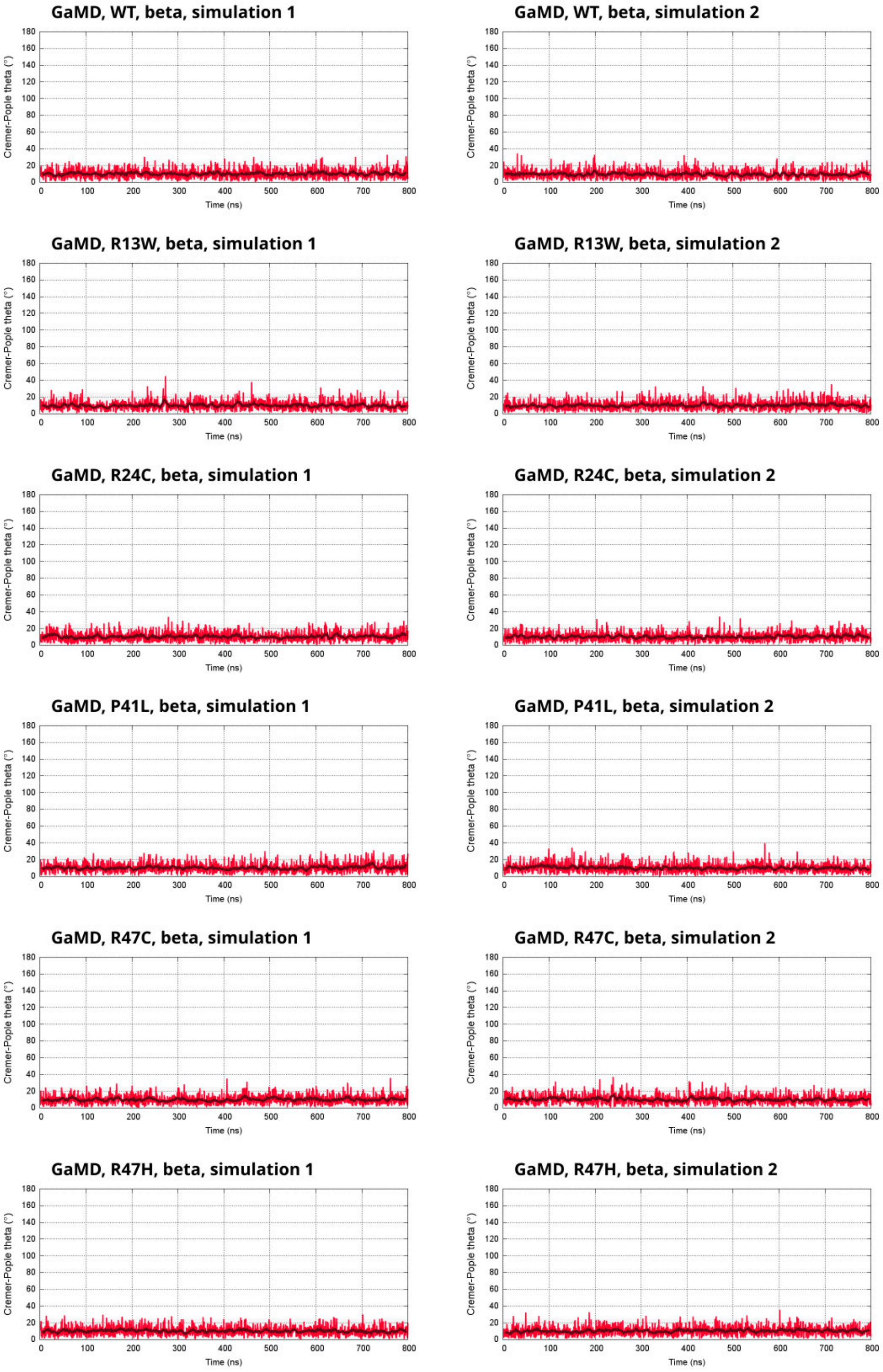

Supplementary Figure S16/A. The Cremer-Pople theta parameter for ring D in each GaMD simulation of beta AT, as a function of time. In this figure, results from simulations of the WT protein as well as the R13W, R24C, P41L, R47C and R47H mutants are shown.

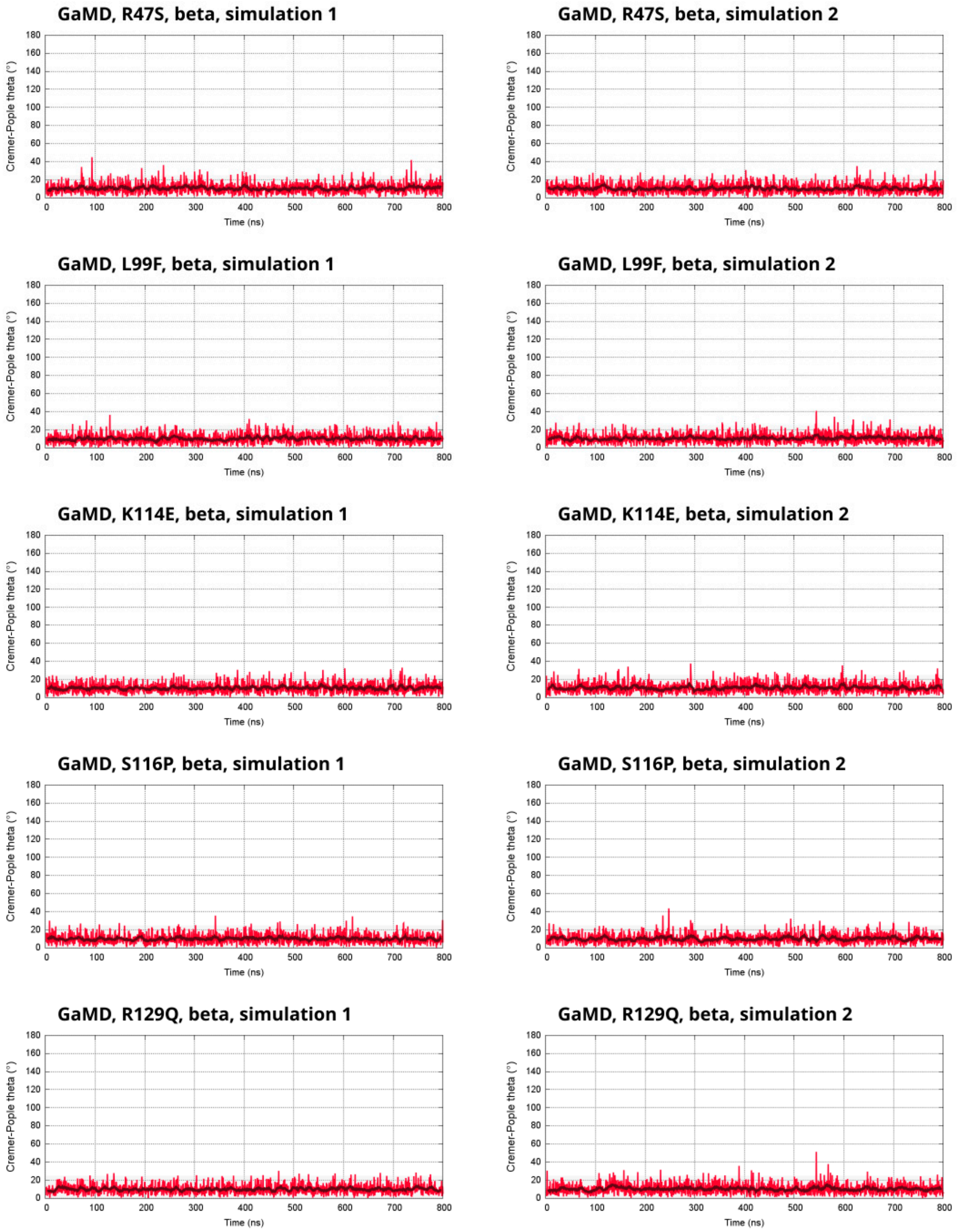

Supplementary Figure S16/B. The Cremer-Pople theta parameter for ring D in each GaMD simulation of beta AT, as a function of time. In this figure, results from simulations of the R47S, L99F, K114E, S116P and R129Q mutants are shown.

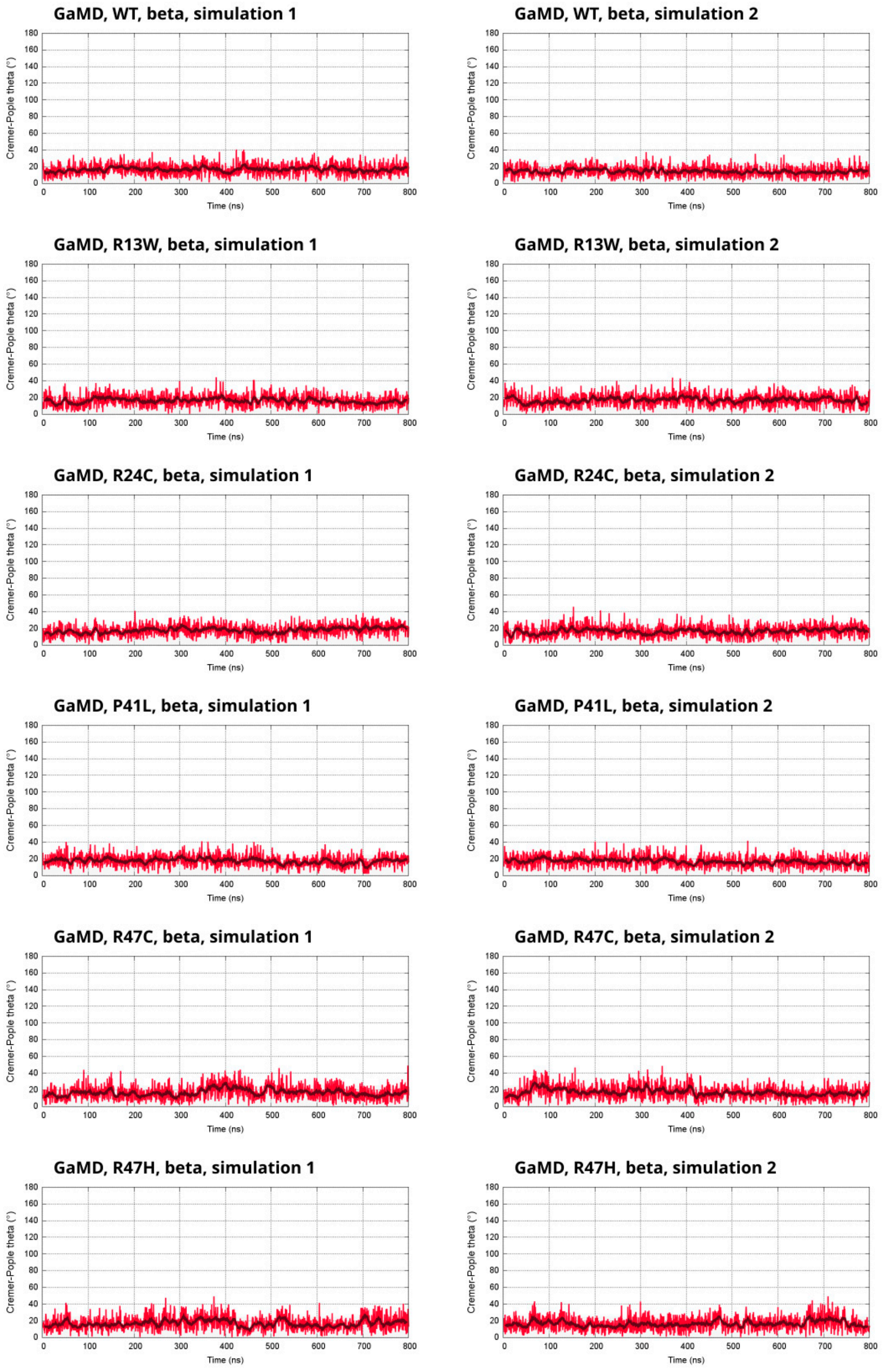

Supplementary Figure S17/A. The Cremer-Pople theta parameter for ring E in each GaMD simulation of beta AT, as a function of time. In this figure, results from simulations of the WT protein as well as the R13W, R24C, P41L, R47C and R47H mutants are shown.

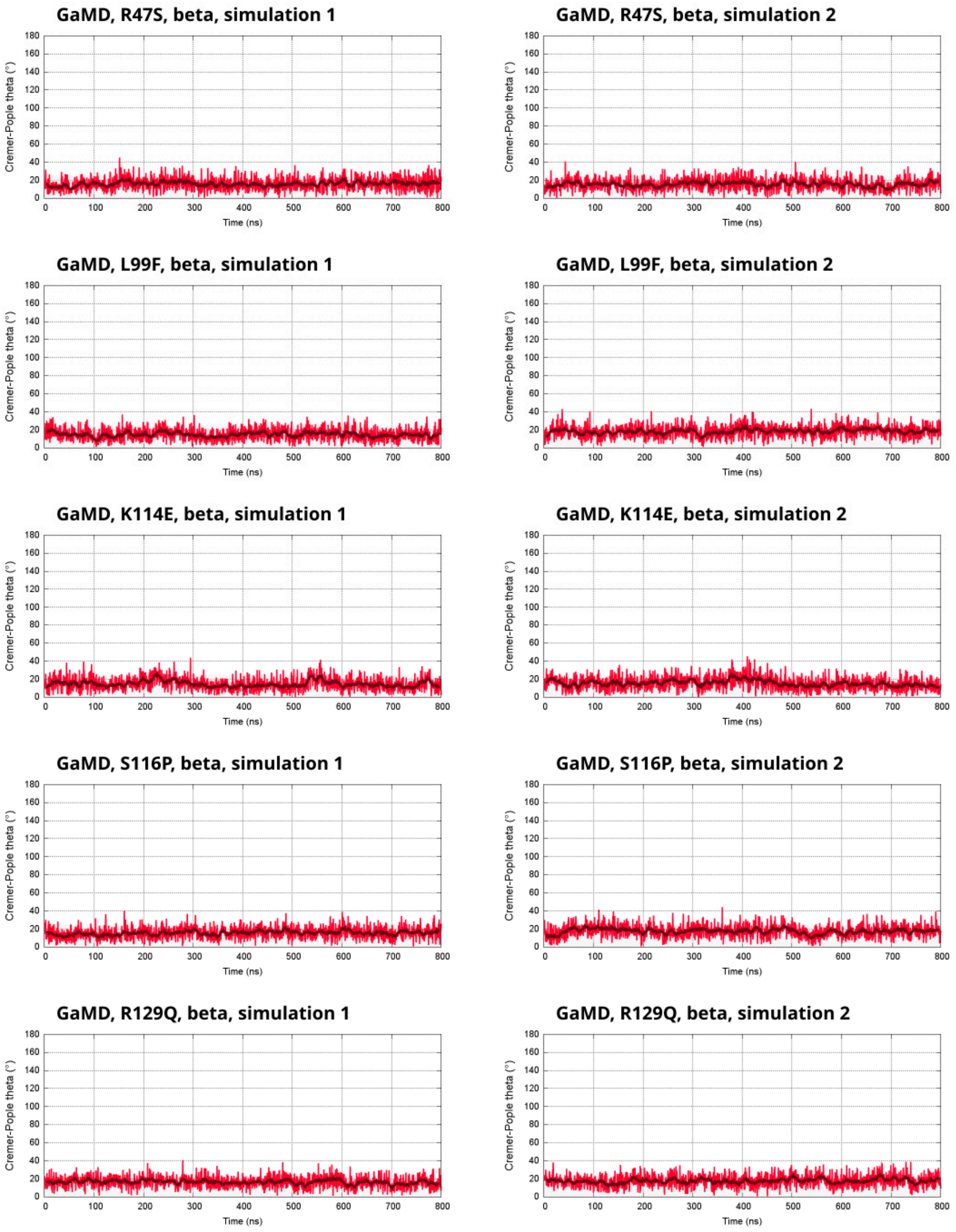

Supplementary Figure S17/B. The Cremer-Pople theta parameter for ring E in each GaMD simulation of beta AT, as a function of time. In this figure, results from simulations of the R47S, L99F, K114E, S116P and R129Q mutants are shown.

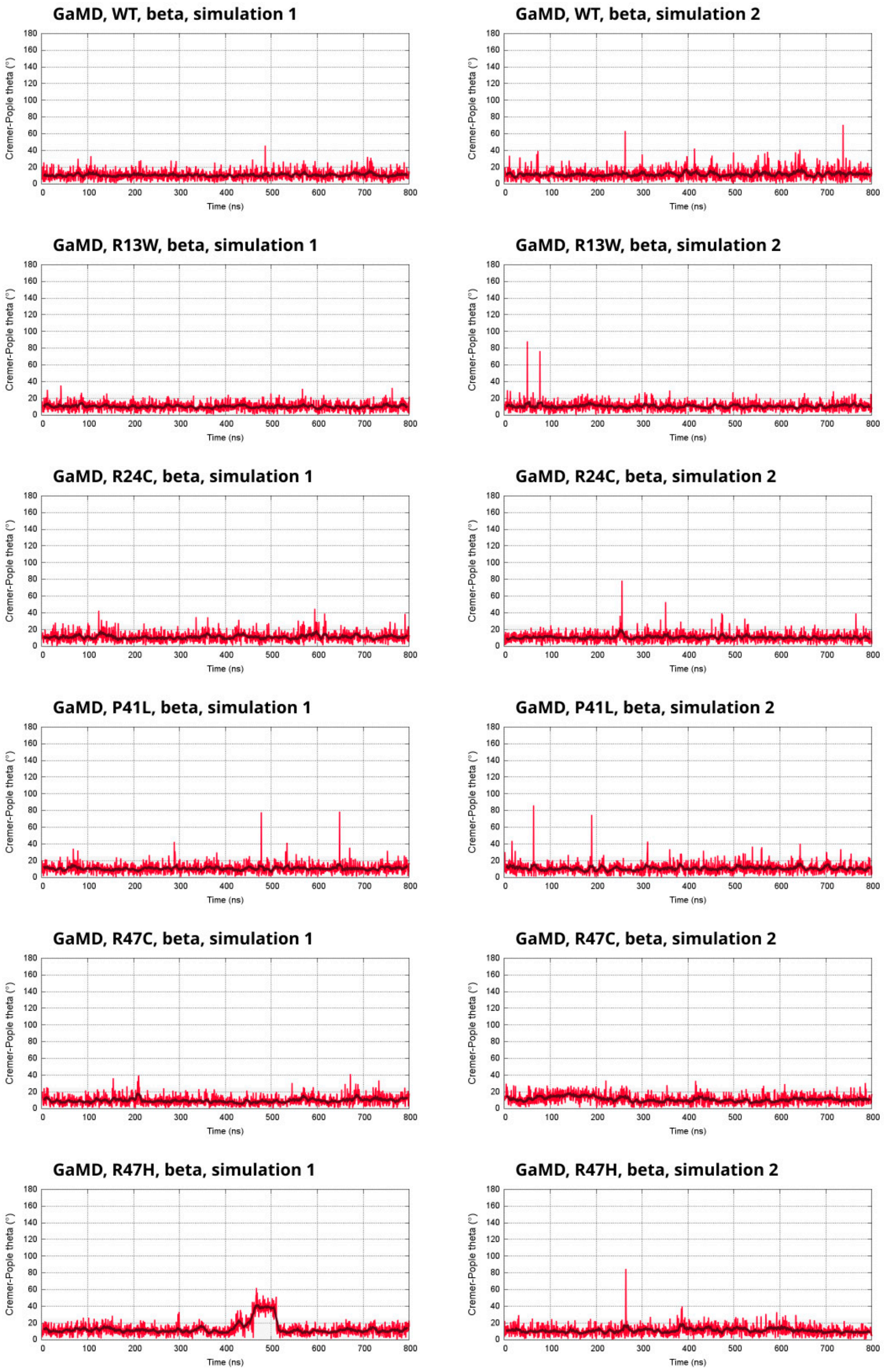

Supplementary Figure S18/A. The Cremer-Pople theta parameter for ring F in each GaMD simulation of beta AT, as a function of time. In this figure, results from simulations of the WT protein as well as the R13W, R24C, P41L, R47C and R47H mutants are shown.

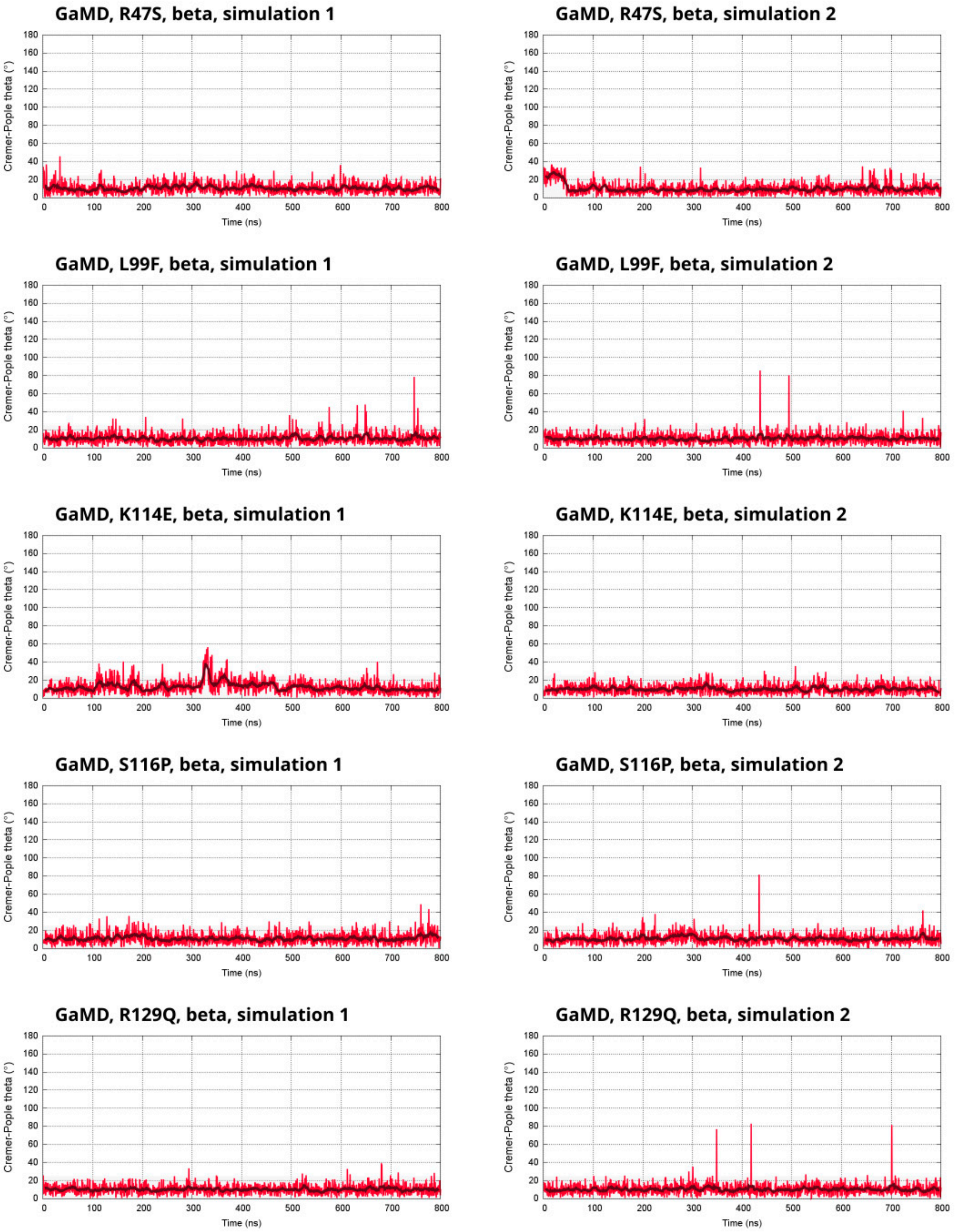

Supplementary Figure S18/B. The Cremer-Pople theta parameter for ring F in each GaMD simulation of beta AT, as a function of time. In this figure, results from simulations of the R47S, L99F, K114E, S116P and R129Q mutants are shown.

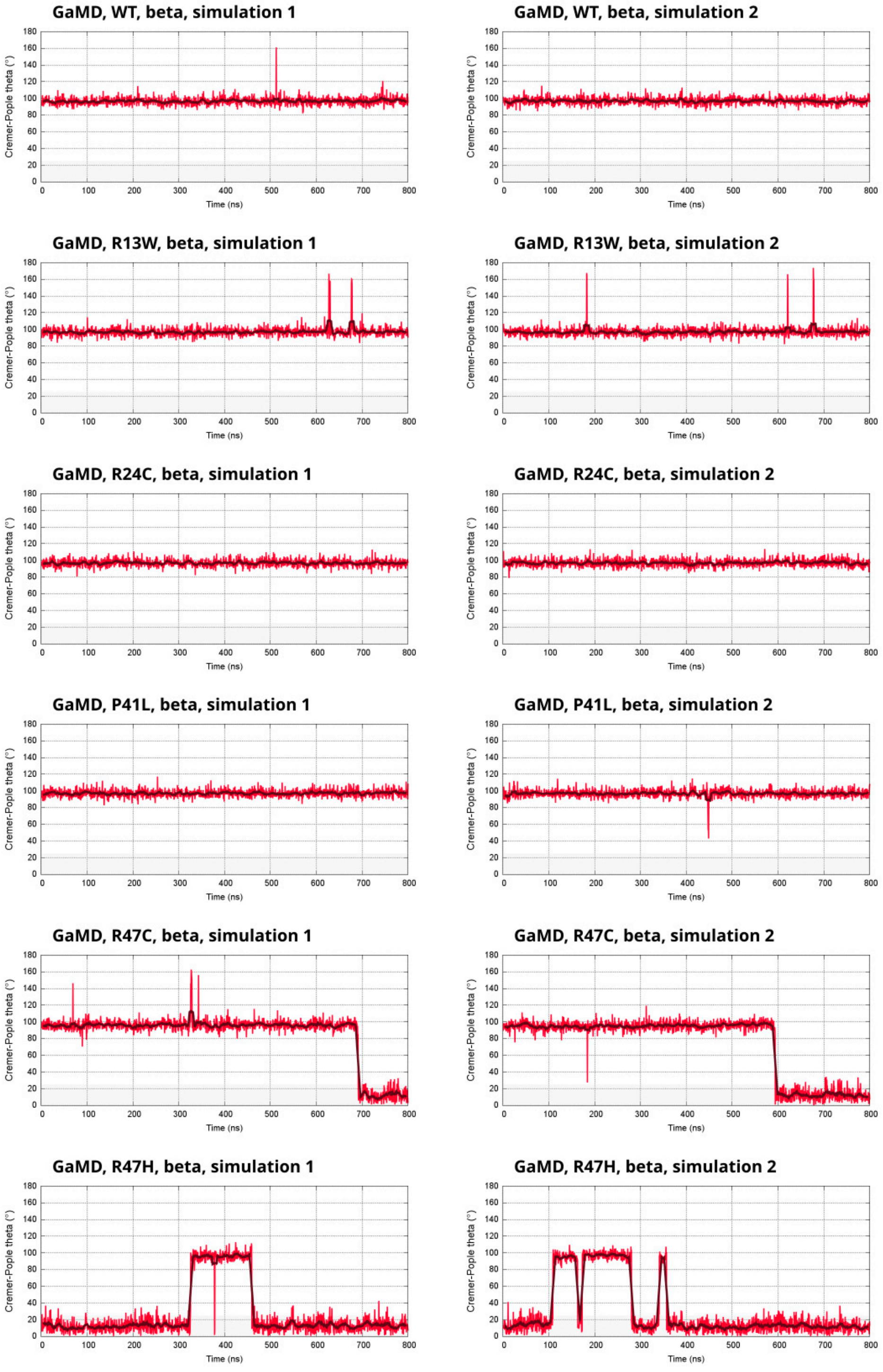

Supplementary Figure S19/A. The Cremer-Pople theta parameter for ring G in each GaMD simulation of beta AT, as a function of time. In this figure, results from simulations of the WT protein as well as the R13W, R24C, P41L, R47C and R47H mutants are shown.

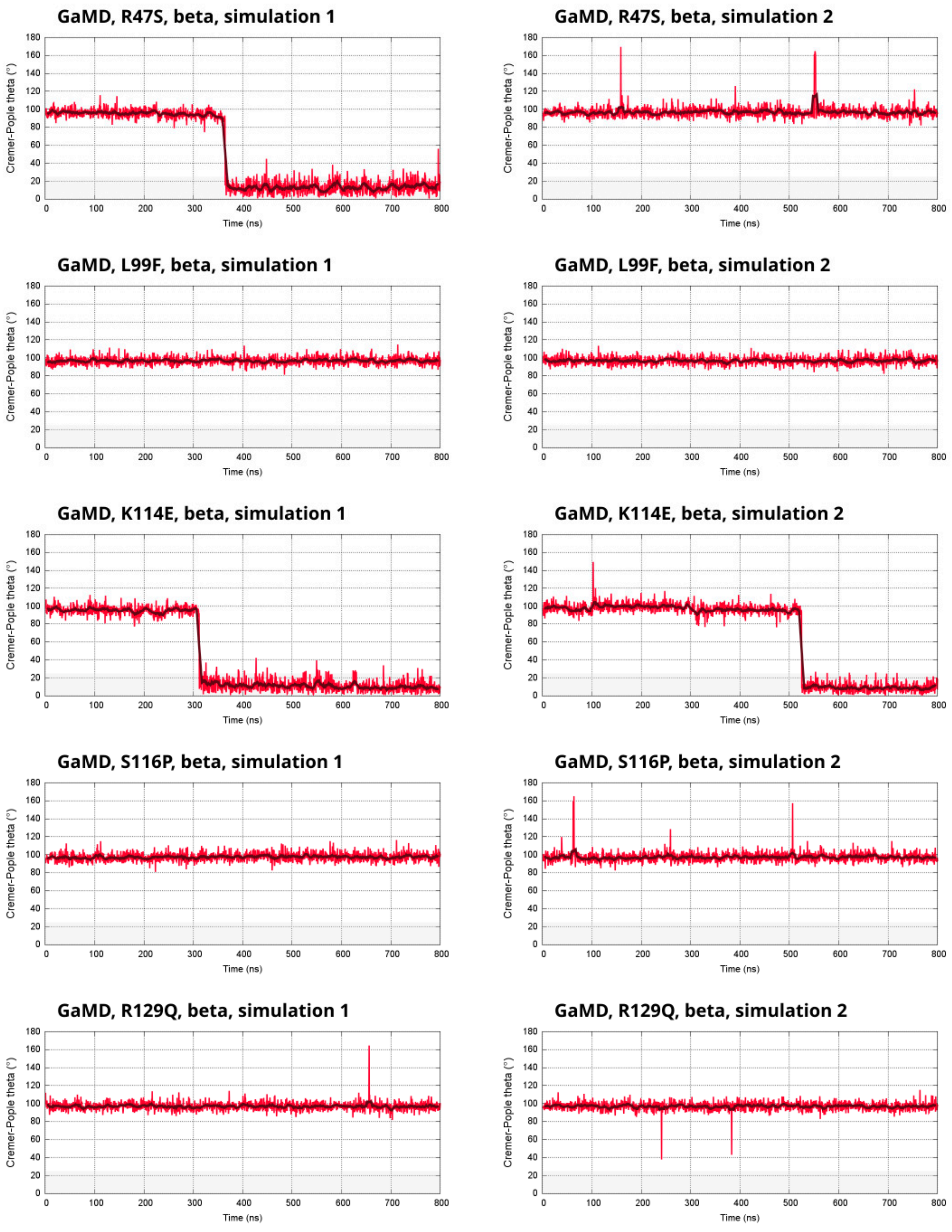

Supplementary Figure S19/B. The Cremer-Pople theta parameter for ring G in each GaMD simulation of beta AT, as a function of time. In this figure, results from simulations of the R47S, L99F, K114E, S116P and R129Q mutants are shown.

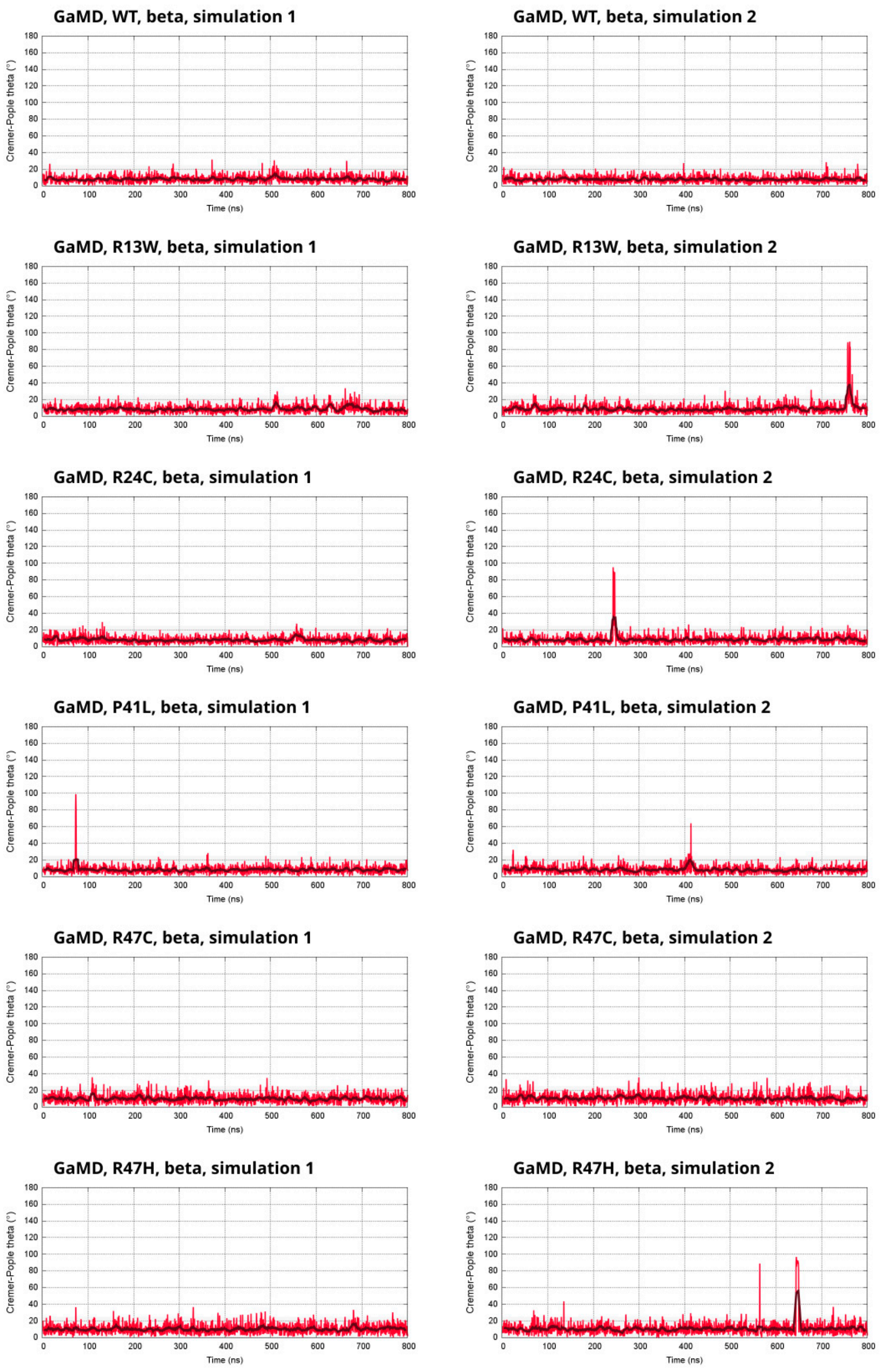

Supplementary Figure S20/A. The Cremer-Pople theta parameter for ring H in each GaMD simulation of beta AT, as a function of time. In this figure, results from simulations of the WT protein as well as the R13W, R24C, P41L, R47C and R47H mutants are shown.

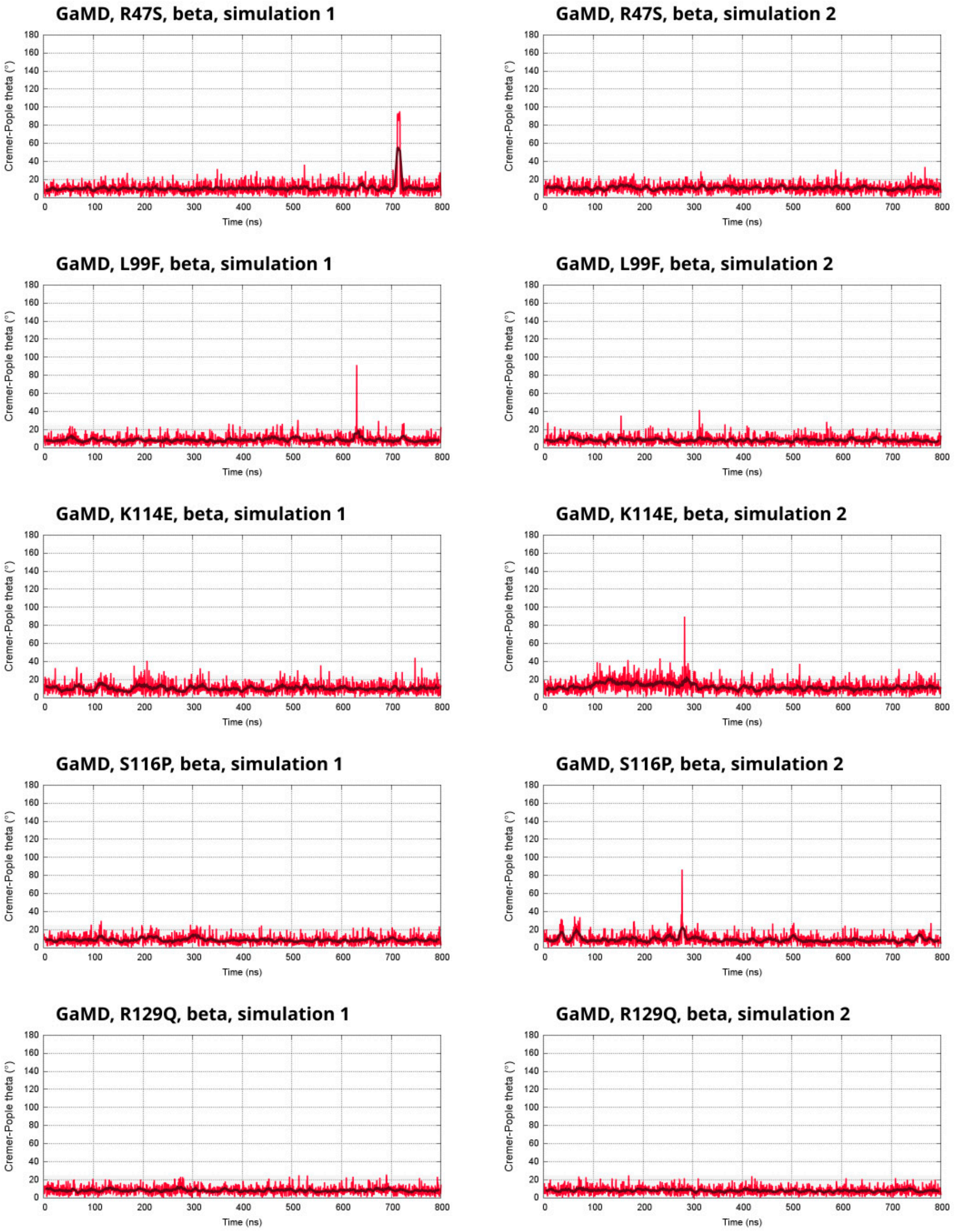

Supplementary Figure S20/B. The Cremer-Pople theta parameter for ring H in each GaMD simulation of beta AT, as a function of time. In this figure, results from simulations of the R47S, L99F, K114E, S116P and R129Q mutants are shown.

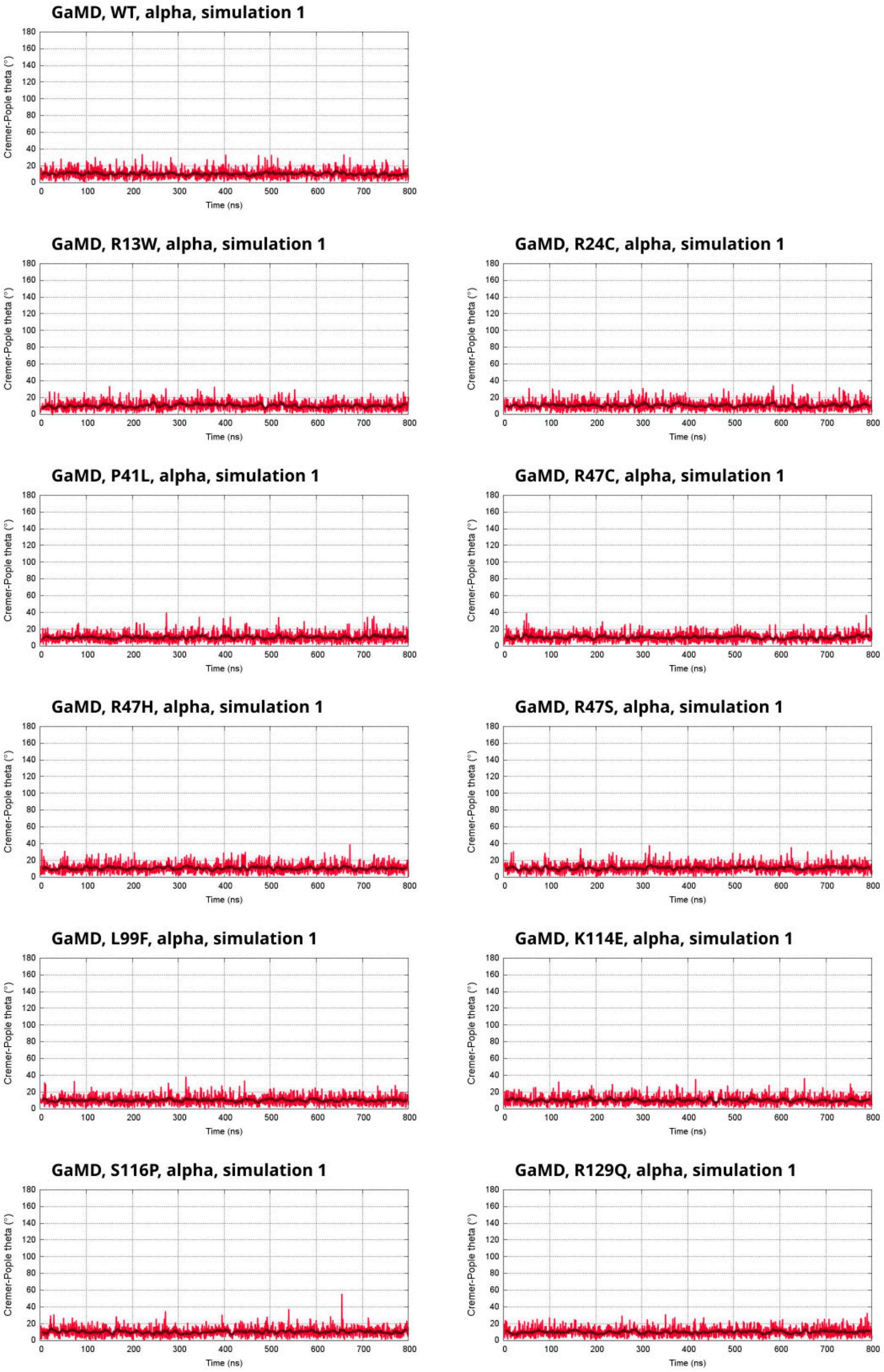

Supplementary Figure S21. The Cremer-Pople theta parameter for ring D in each GaMD simulation of alpha AT, as a function of time.

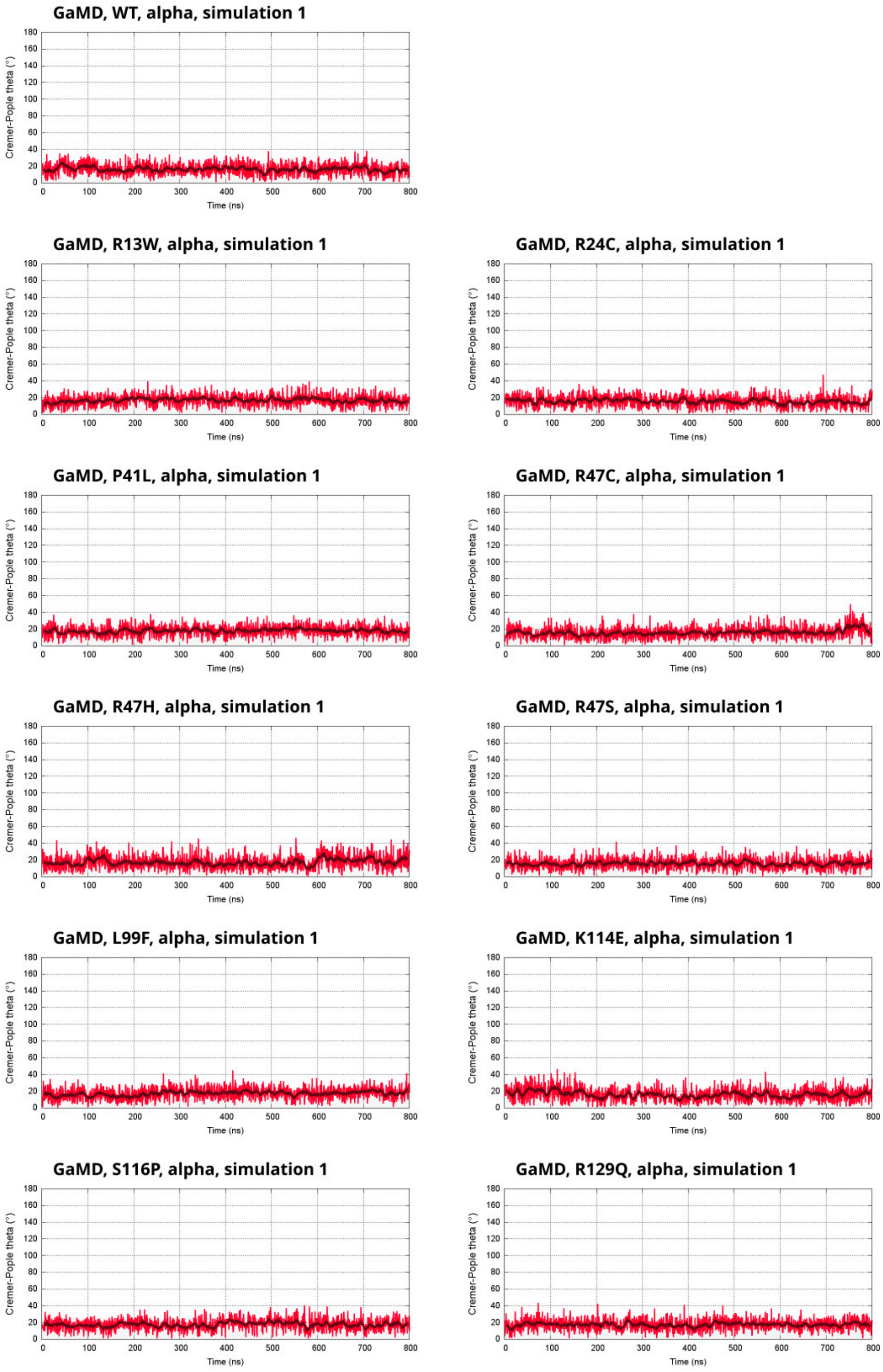

Supplementary Figure S22. The Cremer-Pople theta parameter for ring E in each GaMD simulation of alpha AT, as a function of time.

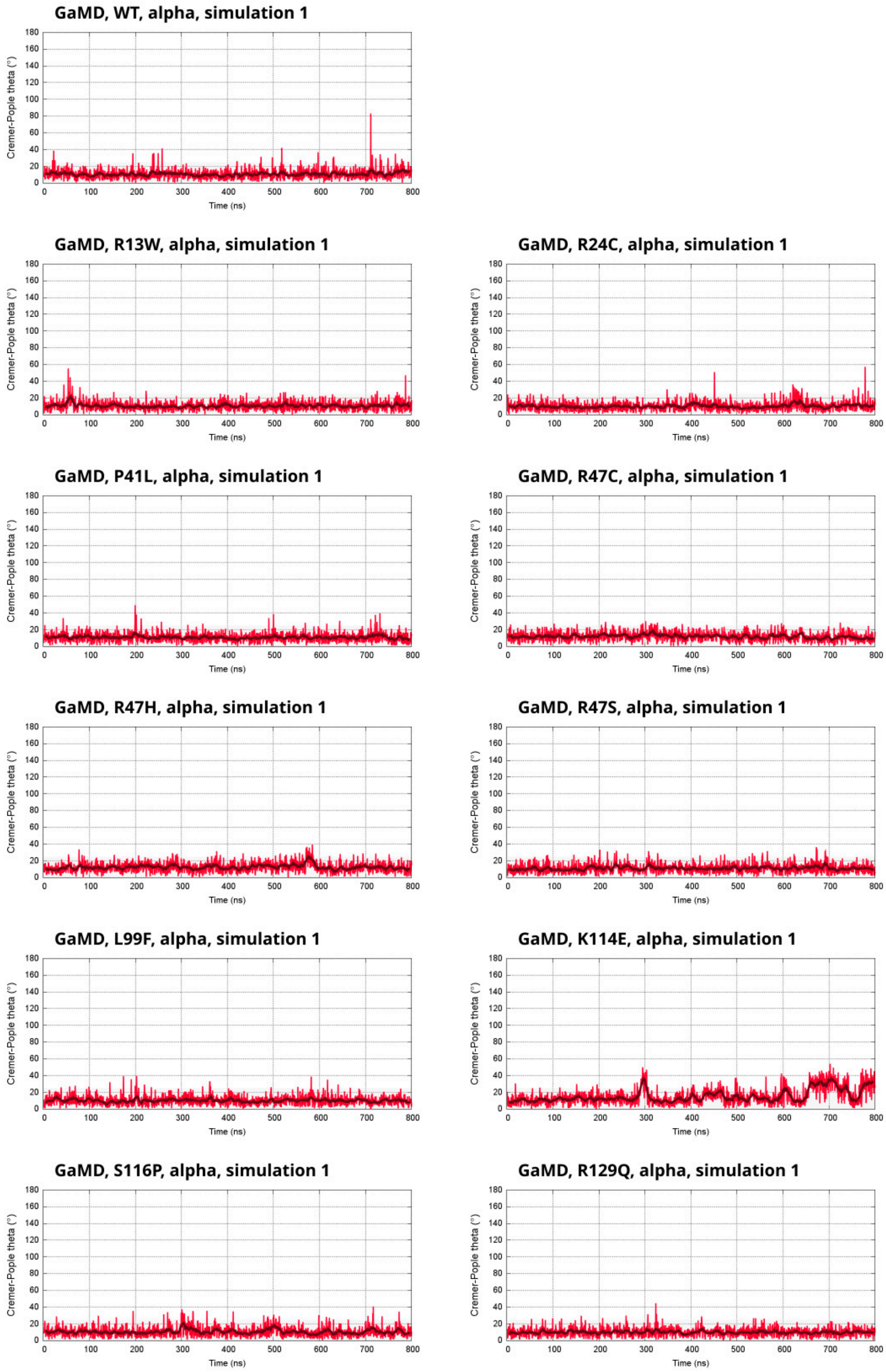

Supplementary Figure S23. The Cremer-Pople theta parameter for ring F in each GaMD simulation of alpha AT, as a function of time.

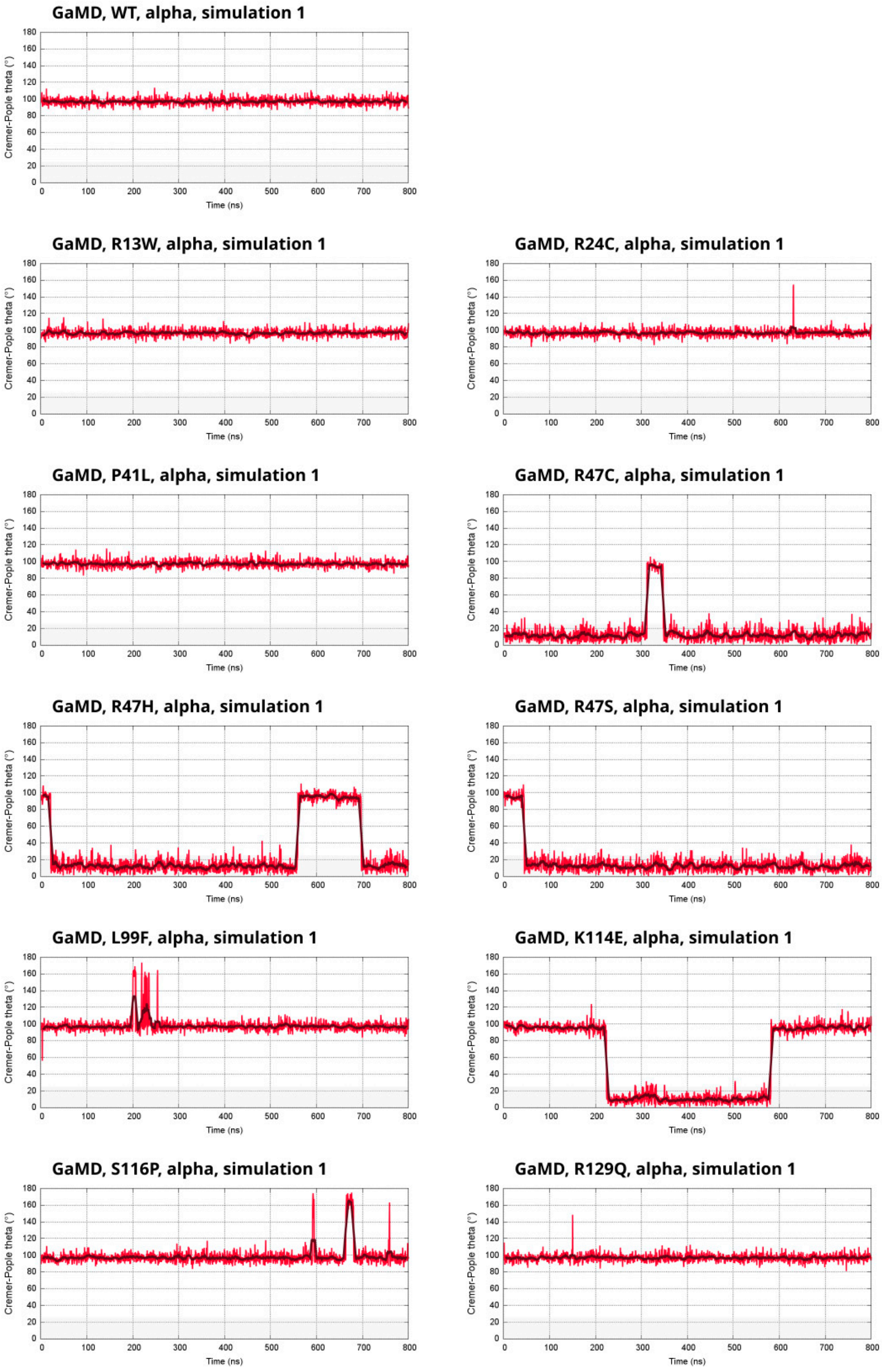

Supplementary Figure S24. The Cremer-Pople theta parameter for ring G in each GaMD simulation of alpha AT, as a function of time.

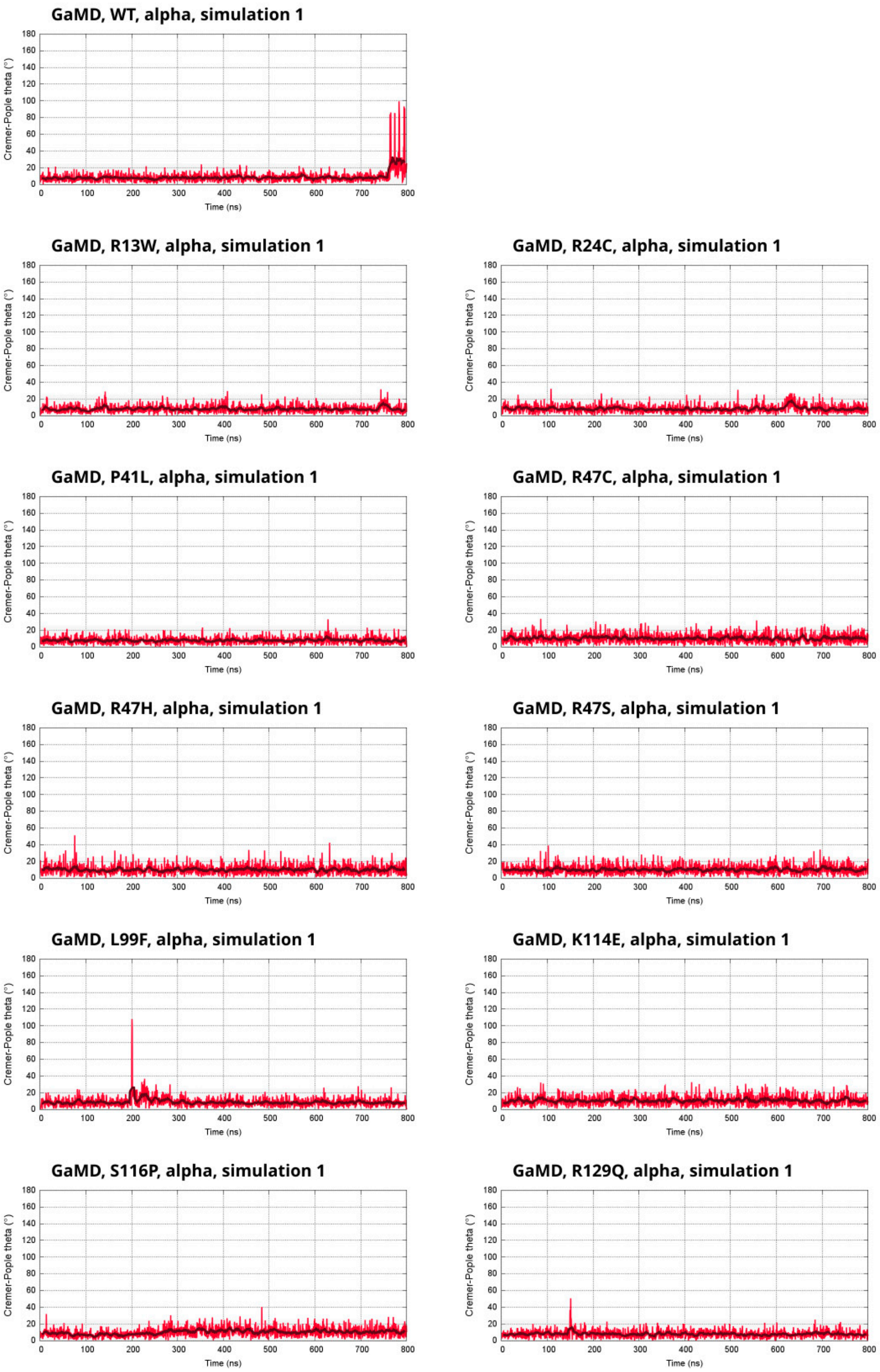

Supplementary Figure S25. The Cremer-Pople theta parameter for ring H in each GaMD simulation of alpha AT, as a function of time.

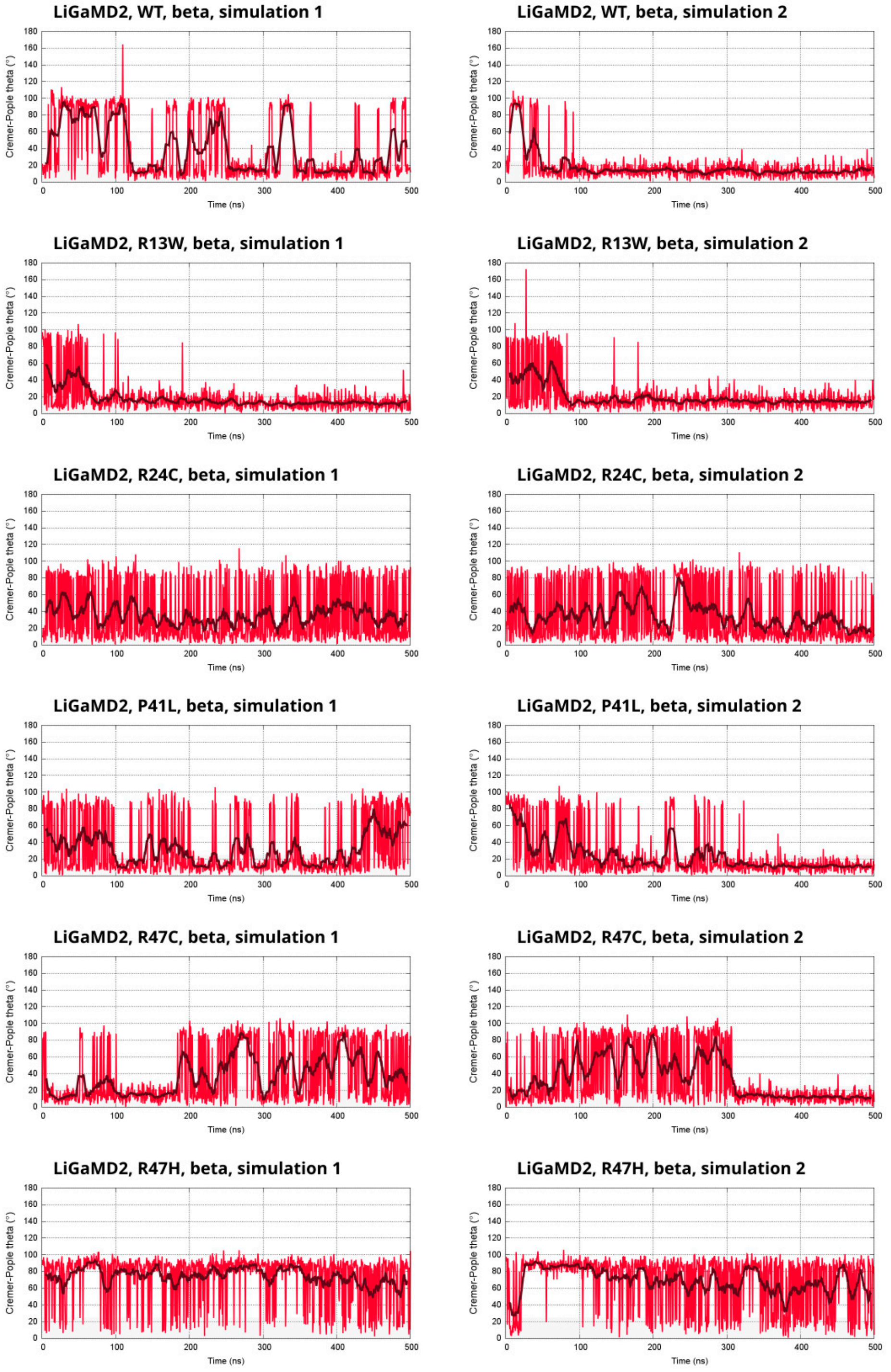

Supplementary Figure S26/A. The Cremer-Pople theta parameter for ring D in each LiGaMD2 simulation of beta AT, as a function of time. In this figure, results from simulations of the WT protein as well as the R13W, R24C, P41L, R47C and R47H mutants are shown.

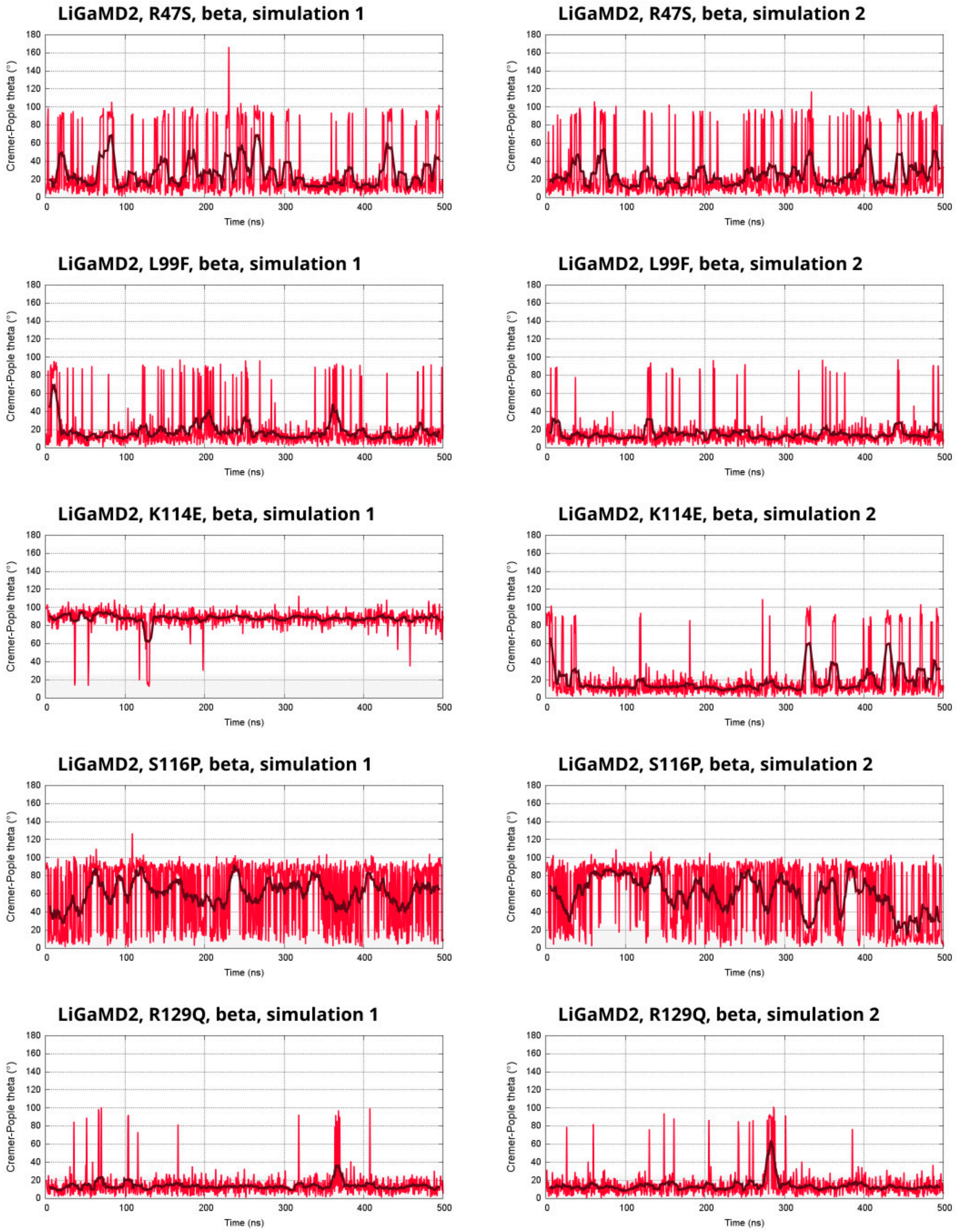

Supplementary Figure S26/B. The Cremer-Pople theta parameter for ring D in each LiGaMD2 simulation of beta AT, as a function of time. In this figure, results from simulations of the R47S, L99F, K114E, S116P and R129Q mutants are shown.

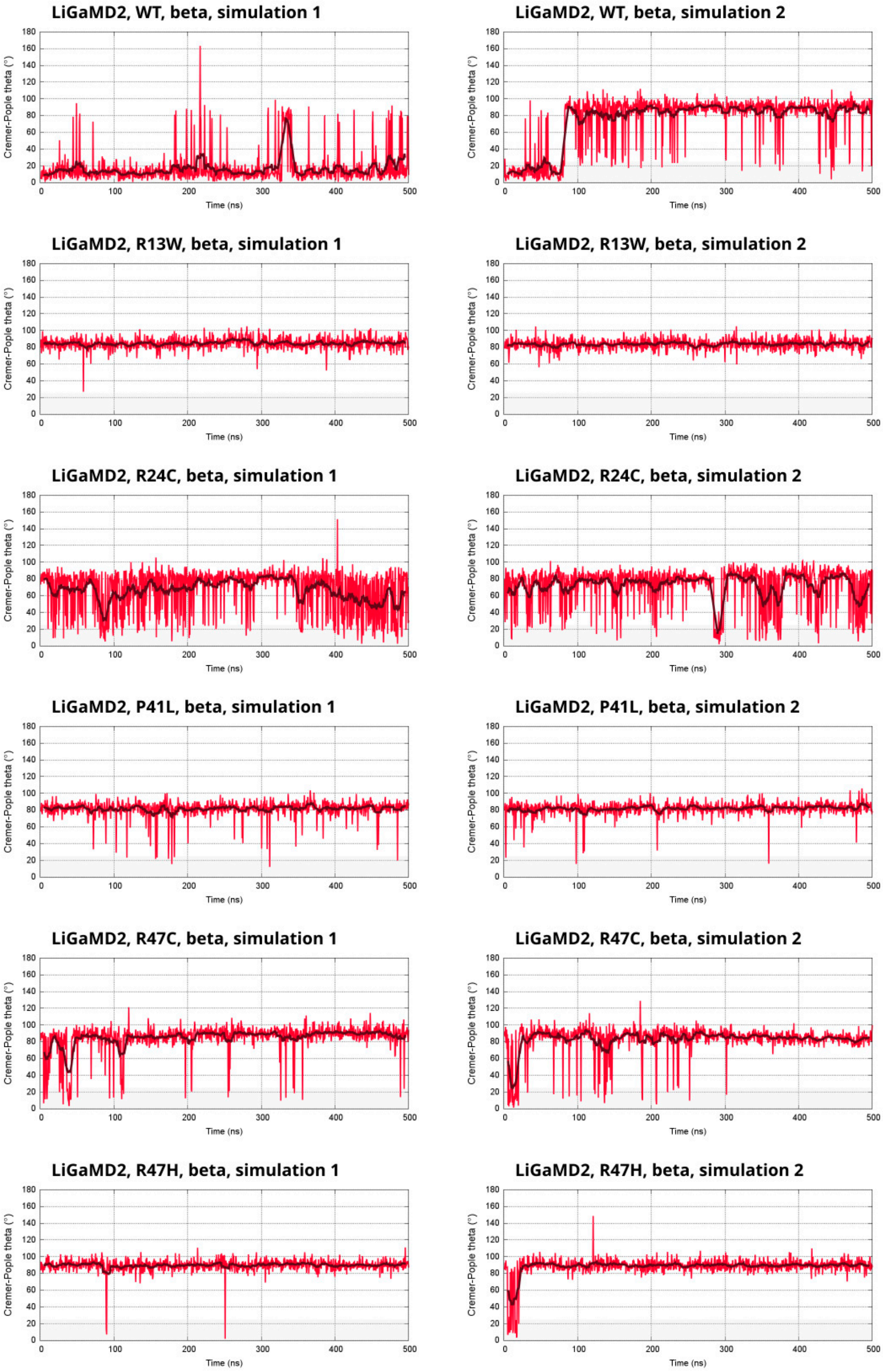

Supplementary Figure S27/A. The Cremer-Pople theta parameter for ring E in each LiGaMD2 simulation of beta AT, as a function of time. In this figure, results from simulations of the WT protein as well as the R13W, R24C, P41L, R47C and R47H mutants are shown.

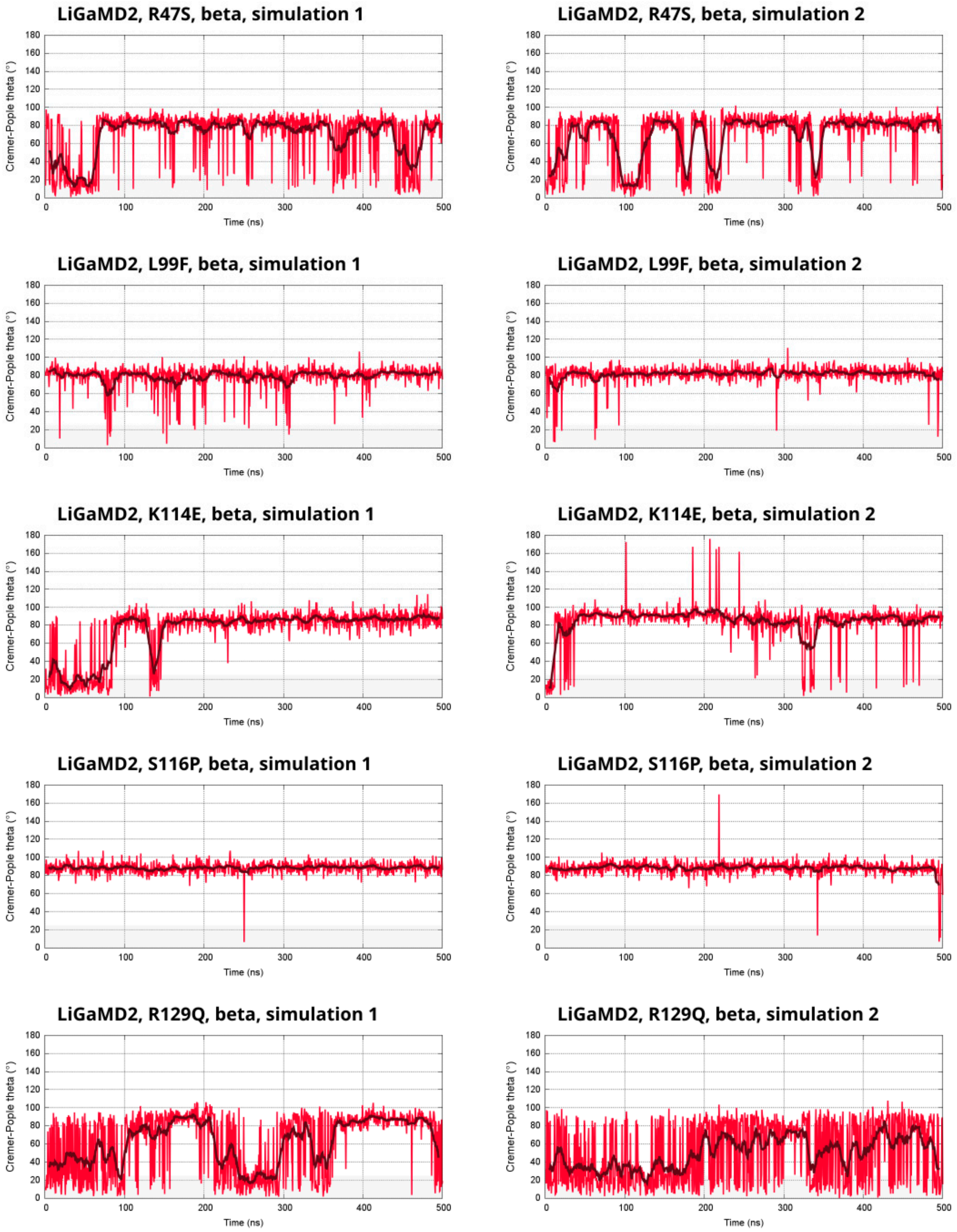

Supplementary Figure S27/B. The Cremer-Pople theta parameter for ring E in each LiGaMD2 simulation of beta AT, as a function of time. In this figure, results from simulations of the R47S, L99F, K114E, S116P and R129Q mutants are shown.

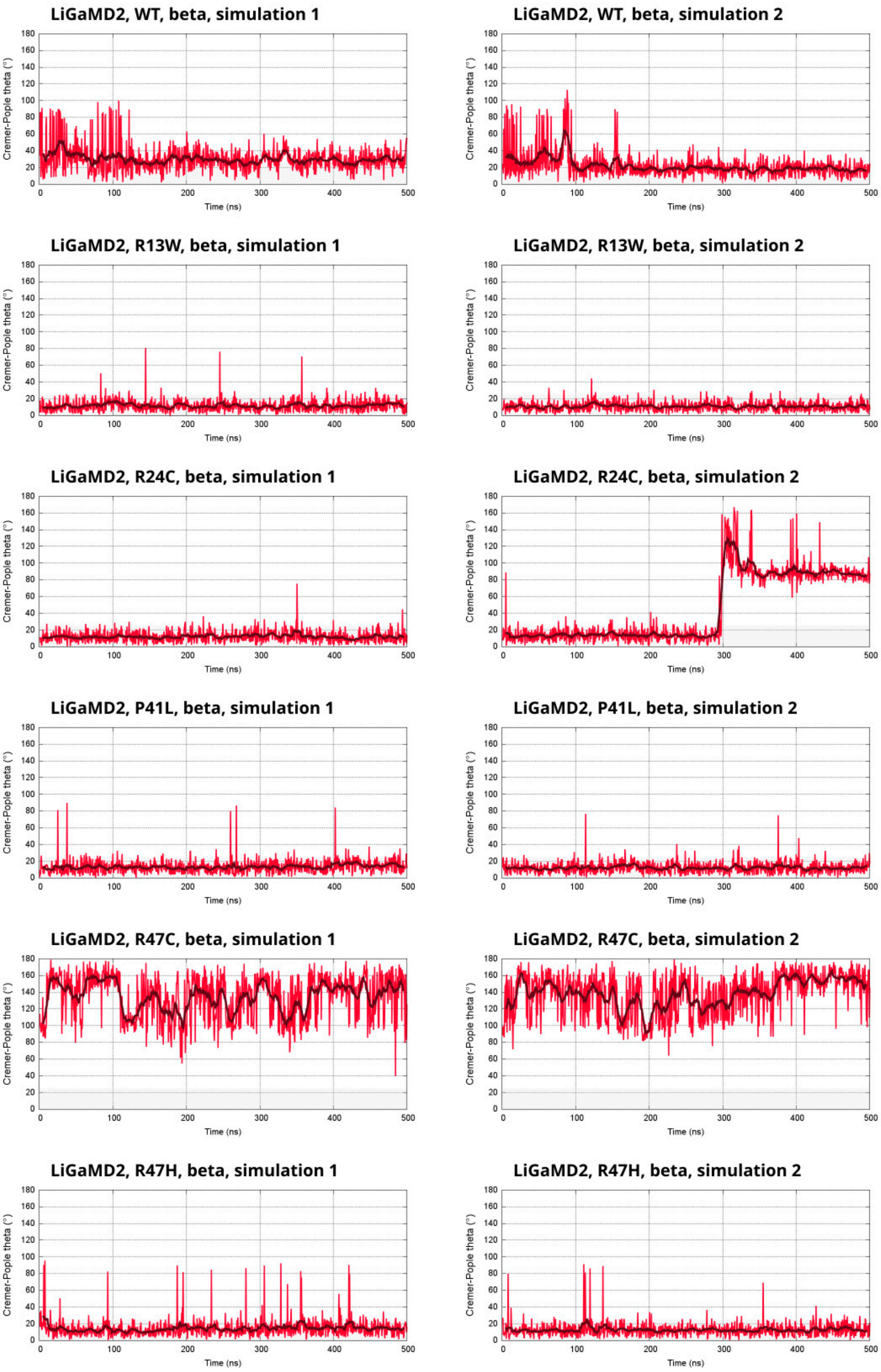

Supplementary Figure S28/A. The Cremer-Pople theta parameter for ring F in each LiGaMD2 simulation of beta AT, as a function of time. In this figure, results from simulations of the WT protein as well as the R13W, R24C, P41L, R47C and R47H mutants are shown.

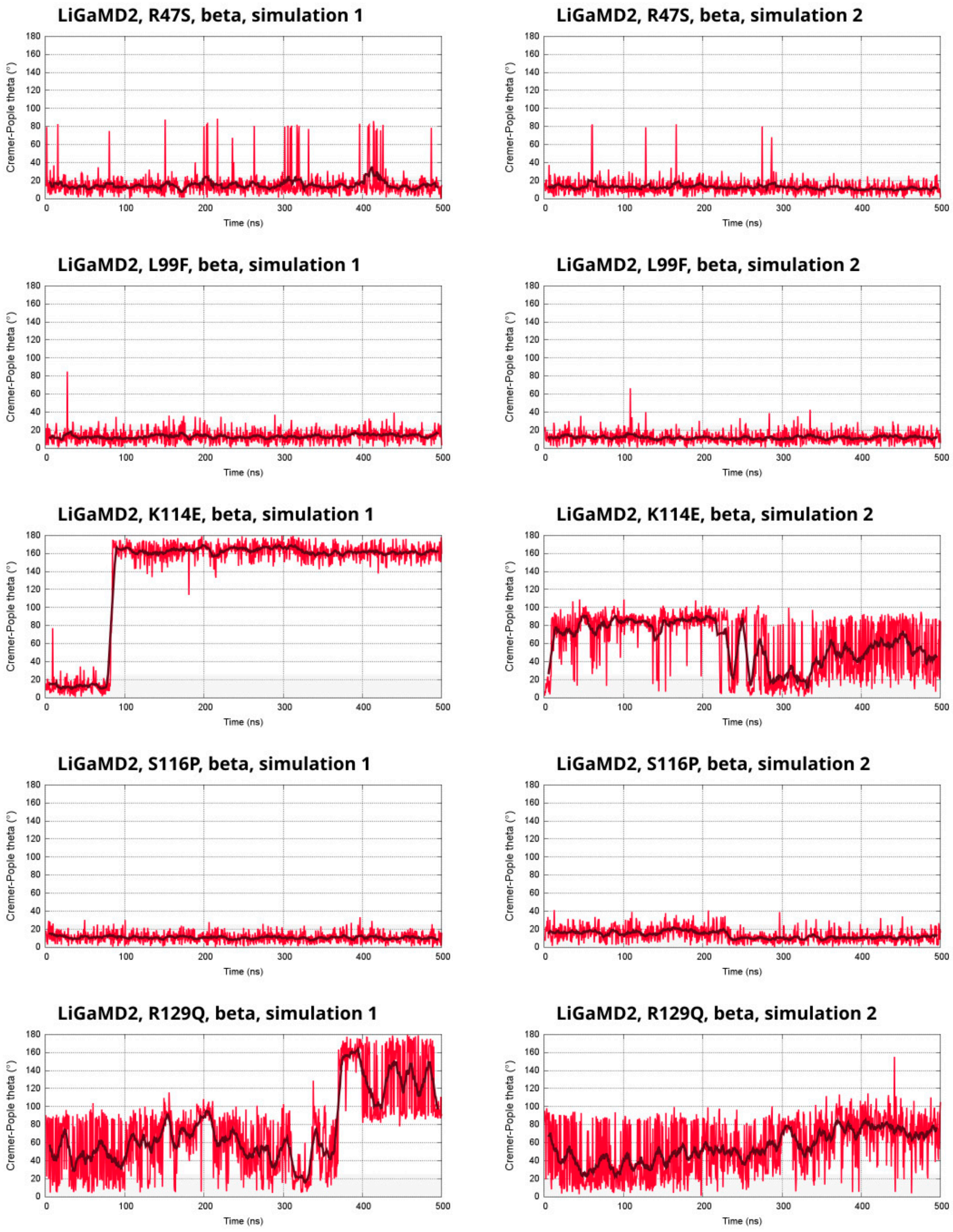

Supplementary Figure S28/B. The Cremer-Pople theta parameter for ring F in each LiGaMD2 simulation of beta AT, as a function of time. In this figure, results from simulations of the R47S, L99F, K114E, S116P and R129Q mutants are shown.

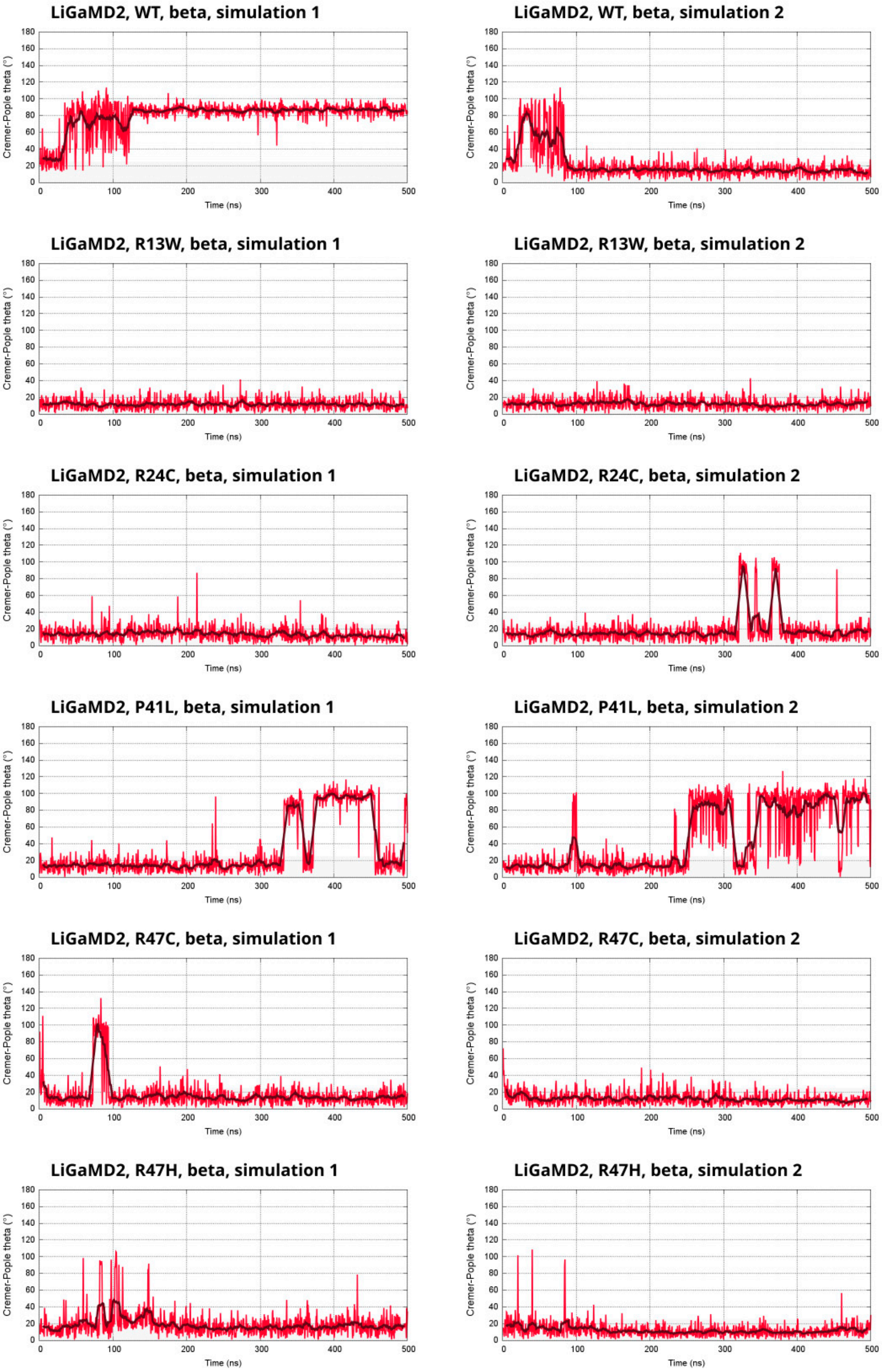

Supplementary Figure S29/A. The Cremer-Pople theta parameter for ring G in each LiGaMD2 simulation of beta AT, as a function of time. In this figure, results from simulations of the WT protein as well as the R13W, R24C, P41L, R47C and R47H mutants are shown.

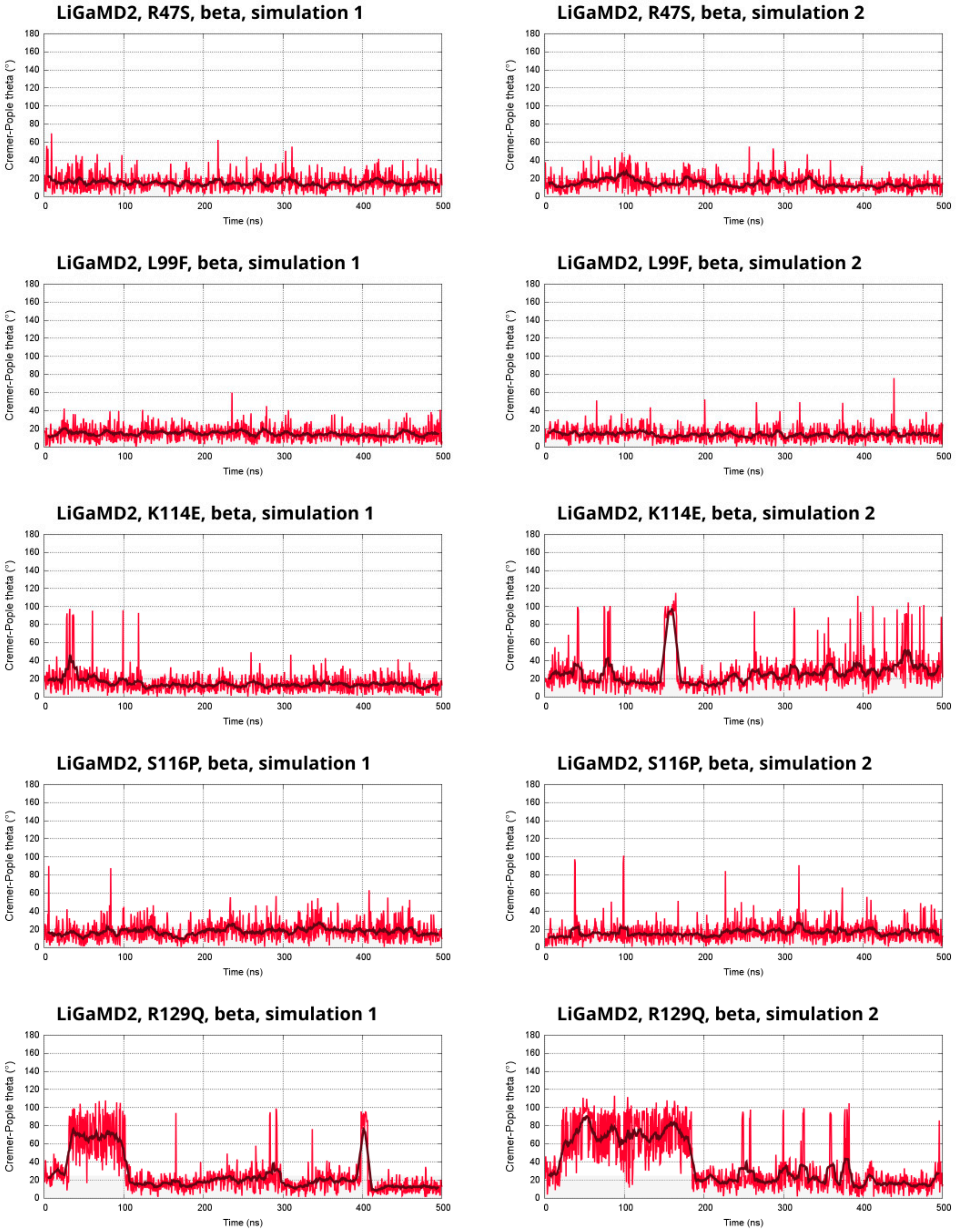

Supplementary Figure S29/B. The Cremer-Pople theta parameter for ring G in each LiGaMD2 simulation of beta AT, as a function of time. In this figure, results from simulations of the R47S, L99F, K114E, S116P and R129Q mutants are shown.

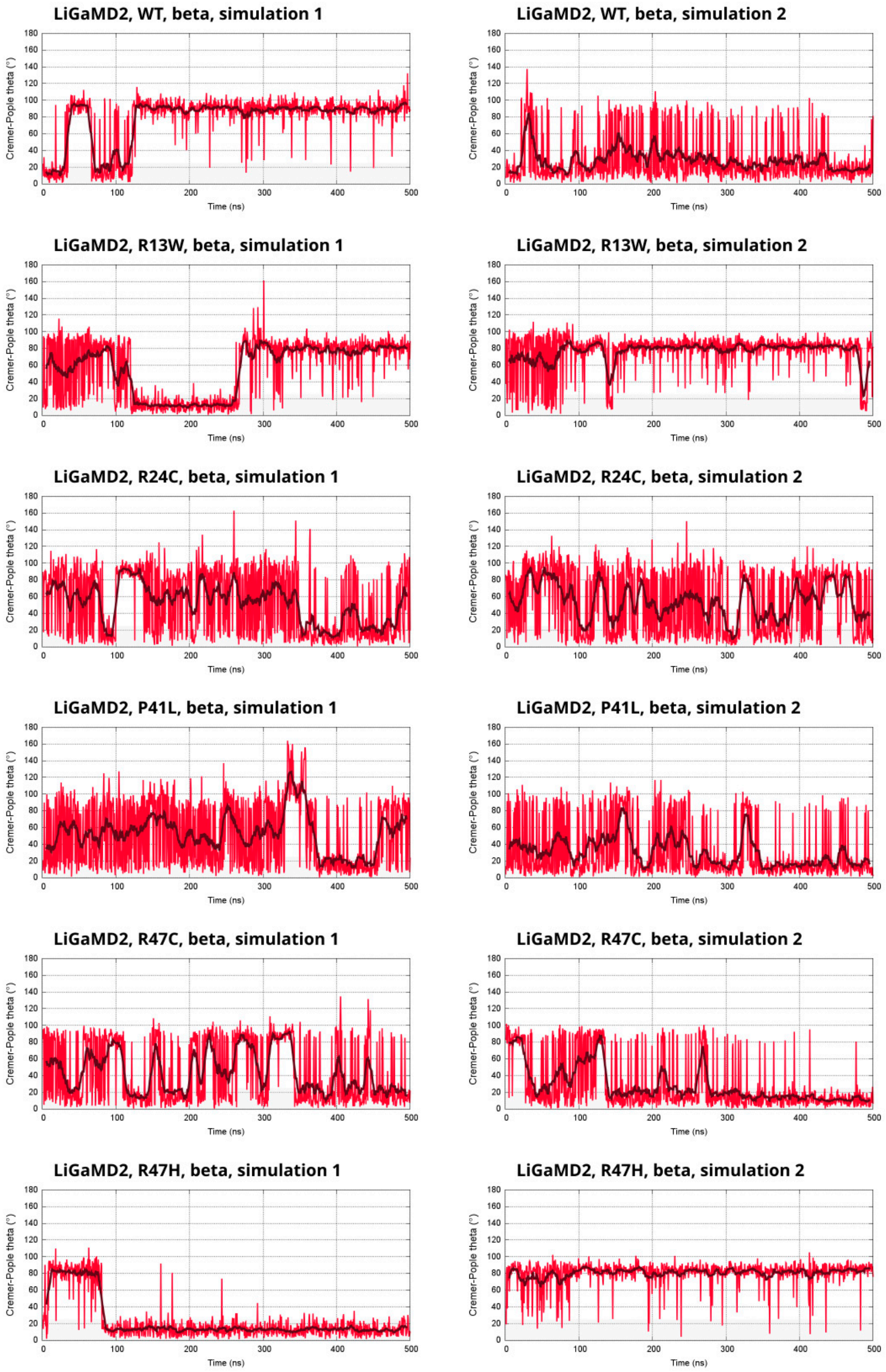

Supplementary Figure S30/A. The Cremer-Pople theta parameter for ring H in each LiGaMD2 simulation of beta AT, as a function of time. In this figure, results from simulations of the WT protein as well as the R13W, R24C, P41L, R47C and R47H mutants are shown.

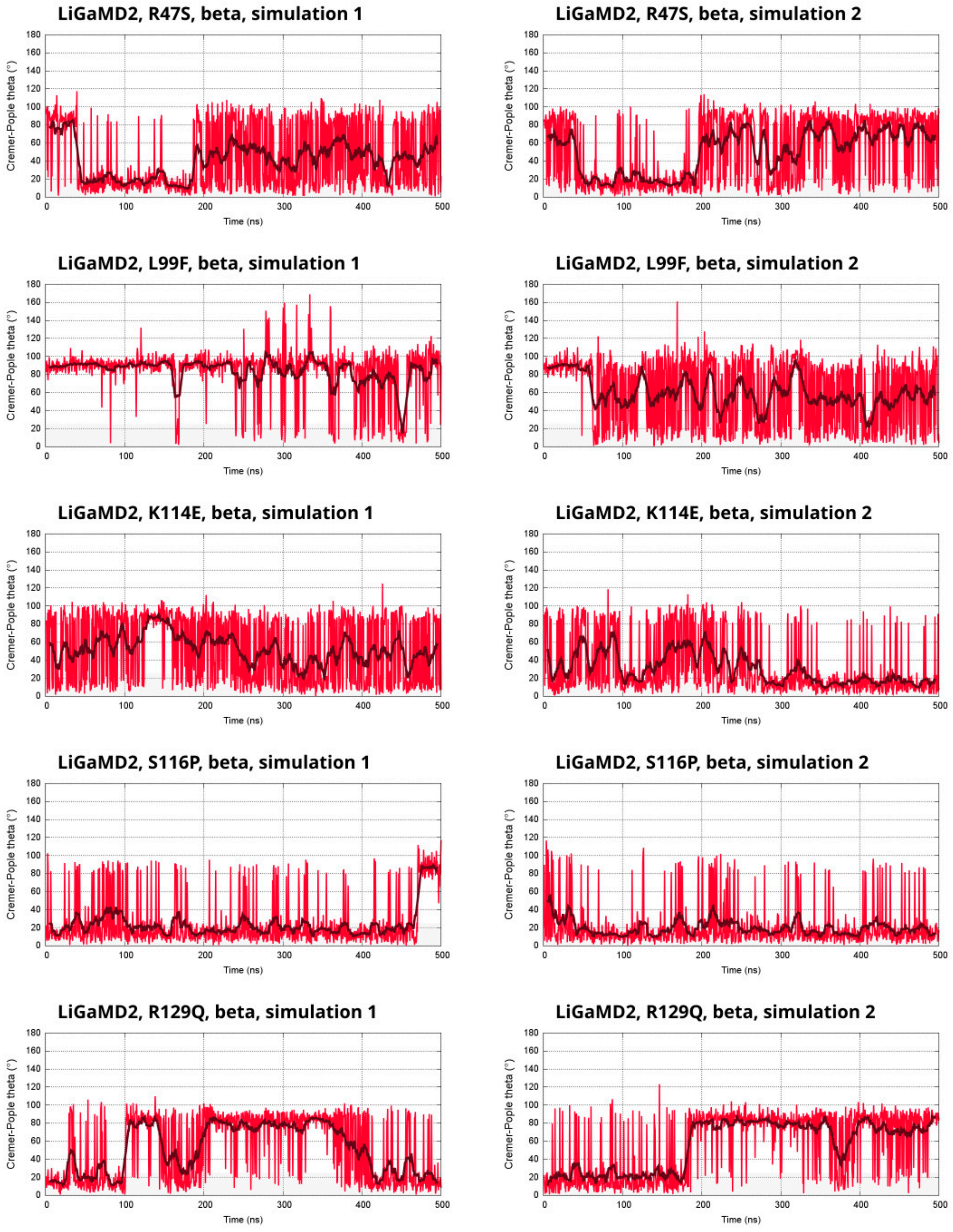

Supplementary Figure S30/B. The Cremer-Pople theta parameter for ring H in each LiGaMD2 simulation of beta AT, as a function of time. In this figure, results from simulations of the R47S, L99F, K114E, S116P and R129Q mutants are shown.

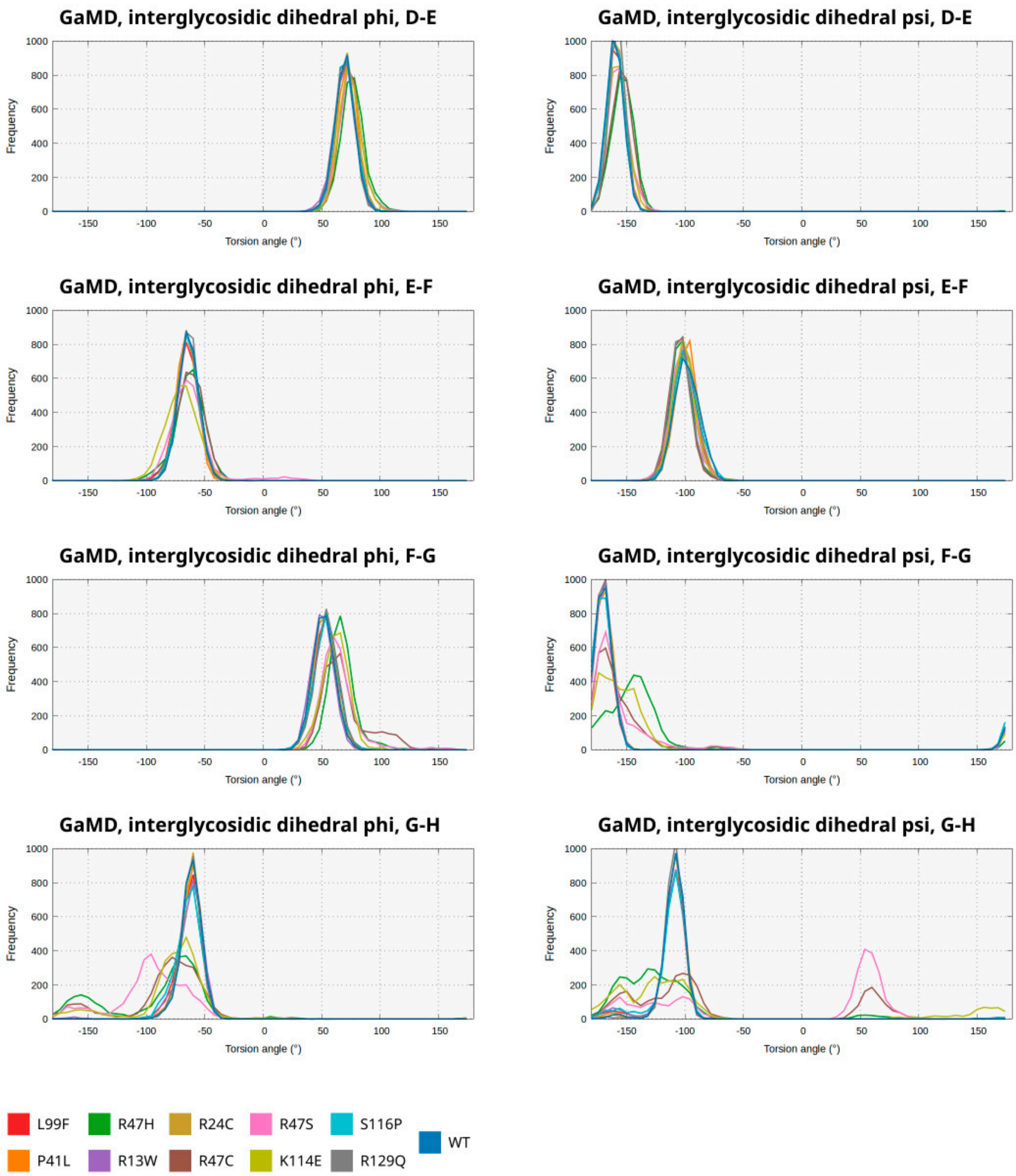

Supplementary Figure S31/A Interglycosidic dihedral angles in the pentasaccharide for all four glycosidic bonds from the GaMD simulations of beta AT.

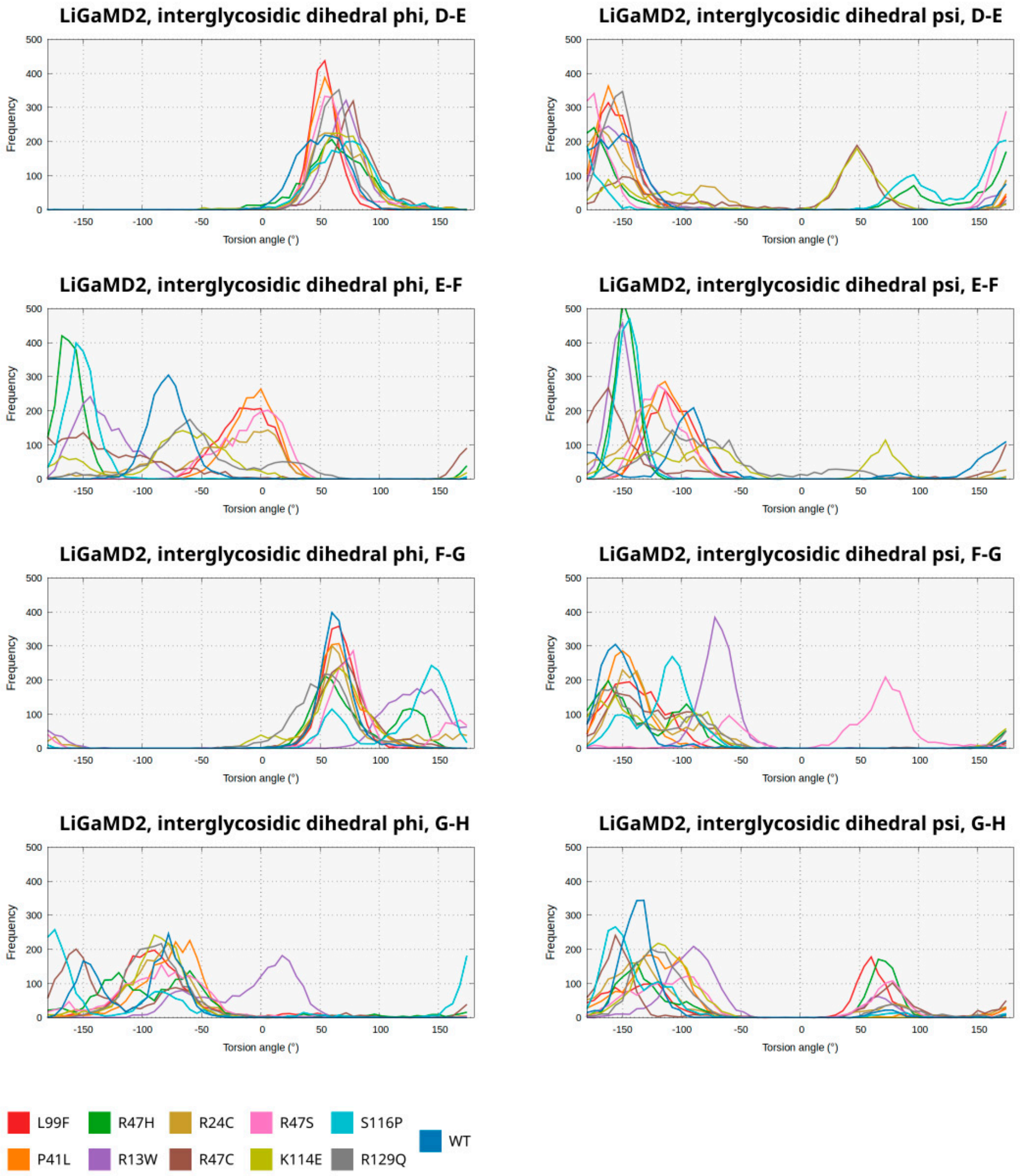

Supplementary Figure S31/B Interglycosidic dihedral angles in the pentasaccharide for all four glycosidic bonds from the LiGaMD2 simulations of beta AT.

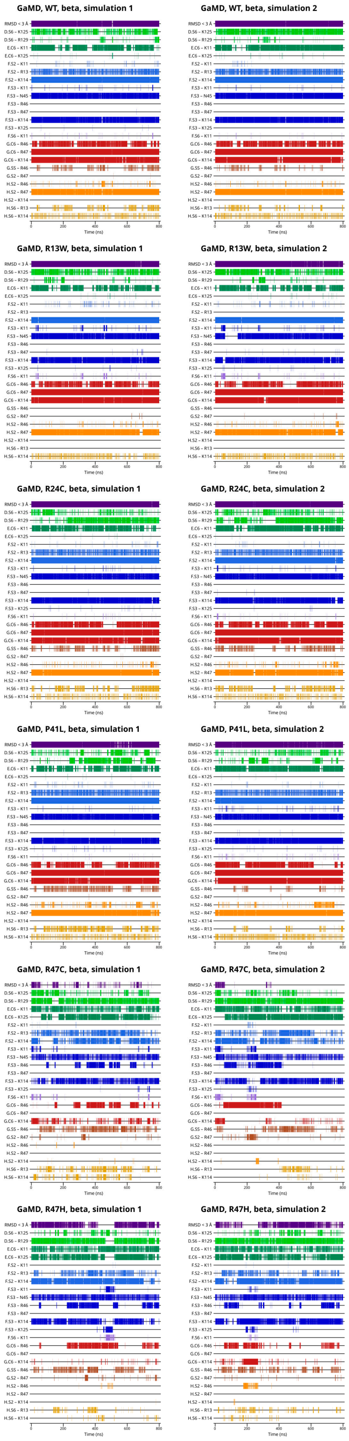

Supplementary Figure S32/A. The amino acids of AT interacting with negatively charged groups in the pentasaccharide (distances below 5 Å) as a function of time, in the GaMD simulations of beta-antithrombin. In this figure, results from simulations of the WT protein as well as the R13W, R24C, P41L, R47C and R47H mutants are shown.

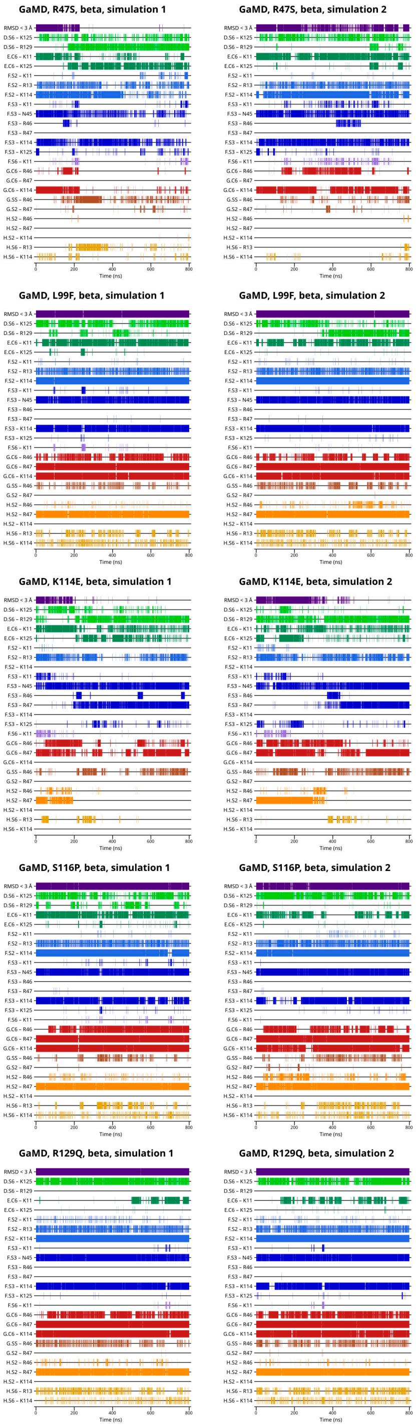

Supplementary Figure S32/B. The amino acids of AT interacting with negatively charged groups in the pentasaccharide (distances below 5 Å) as a function of time, in the GaMD simulations of beta-antithrombin. In this figure, results from simulations of the R47S, L99F, K114E, S116P and R129Q mutants are shown.

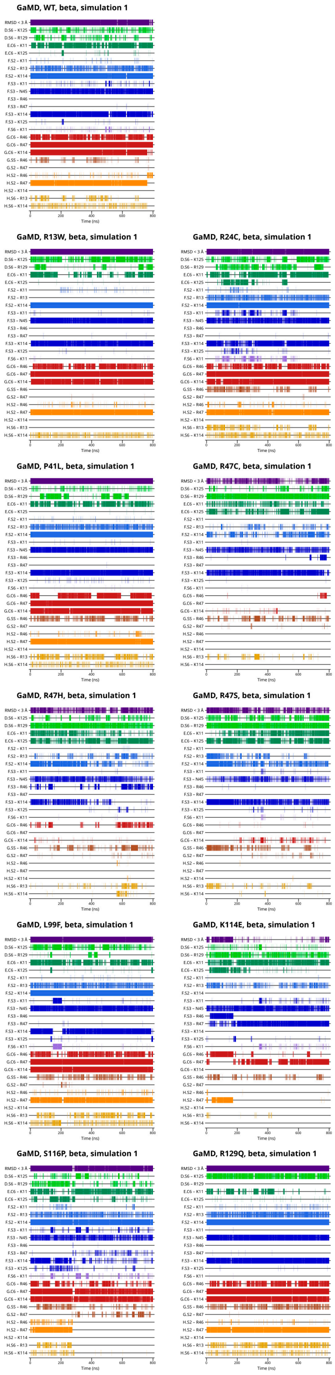

Supplementary Figure S33. The amino acids of AT interacting with negatively charged groups in the pentasaccharide (distances below 5 Å) as a function of time, in the GaMD simulations of alpha-antithrombin.

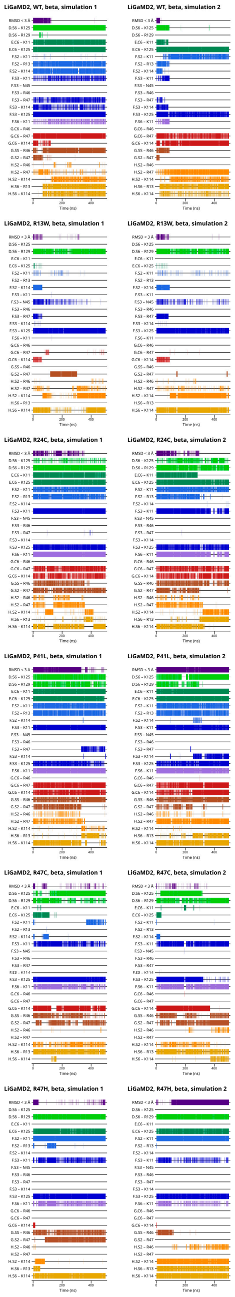

Supplementary Figure S34/A. The amino acids of AT interacting with negatively charged groups in the pentasaccharide (distances below 5 Å) as a function of time, in the LiGaMD2 simulations of beta-antithrombin. In this figure, results from simulations of the WT protein as well as the R13W, R24C, P41L, R47C and R47H mutants are shown.

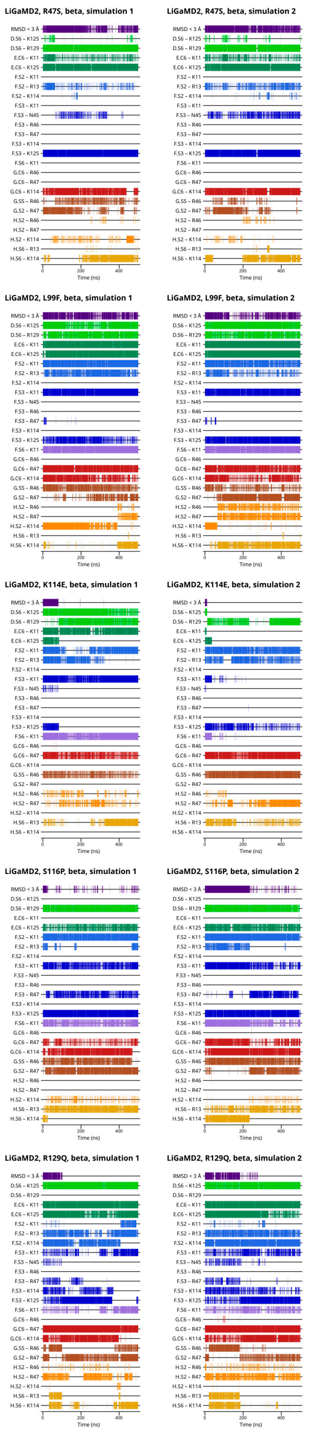

Supplementary Figure S34/B. The amino acids of AT interacting with negatively charged groups in the pentasaccharide (distances below 5 Å) as a function of time, in the LiGaMD2 simulations of beta-antithrombin. In this figure, results from simulations of the R47S, L99F, K114E, S116P and R129Q mutants are shown.

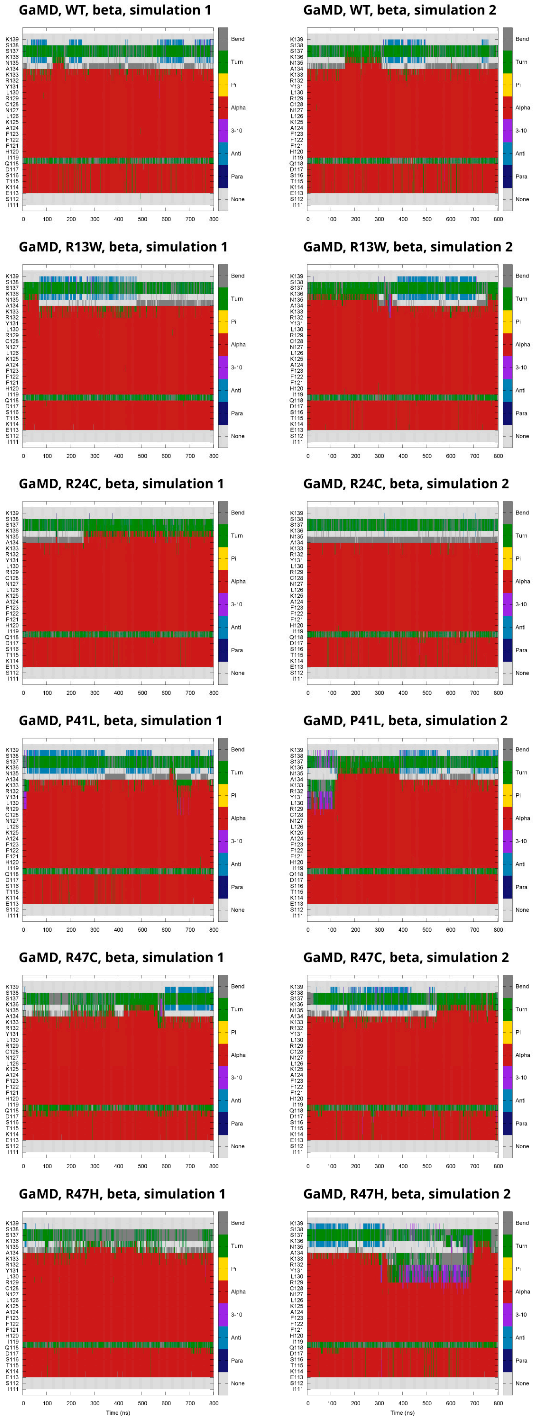

Supplementary Figure S35/A. Result of the DSSP analysis for residues 111-139, close to the heparin binding site, from the GaMD simulations of beta AT. In this figure, results from simulations of the WT protein as well as the R13W, R24C, P41L, R47C and R47H mutants are shown.

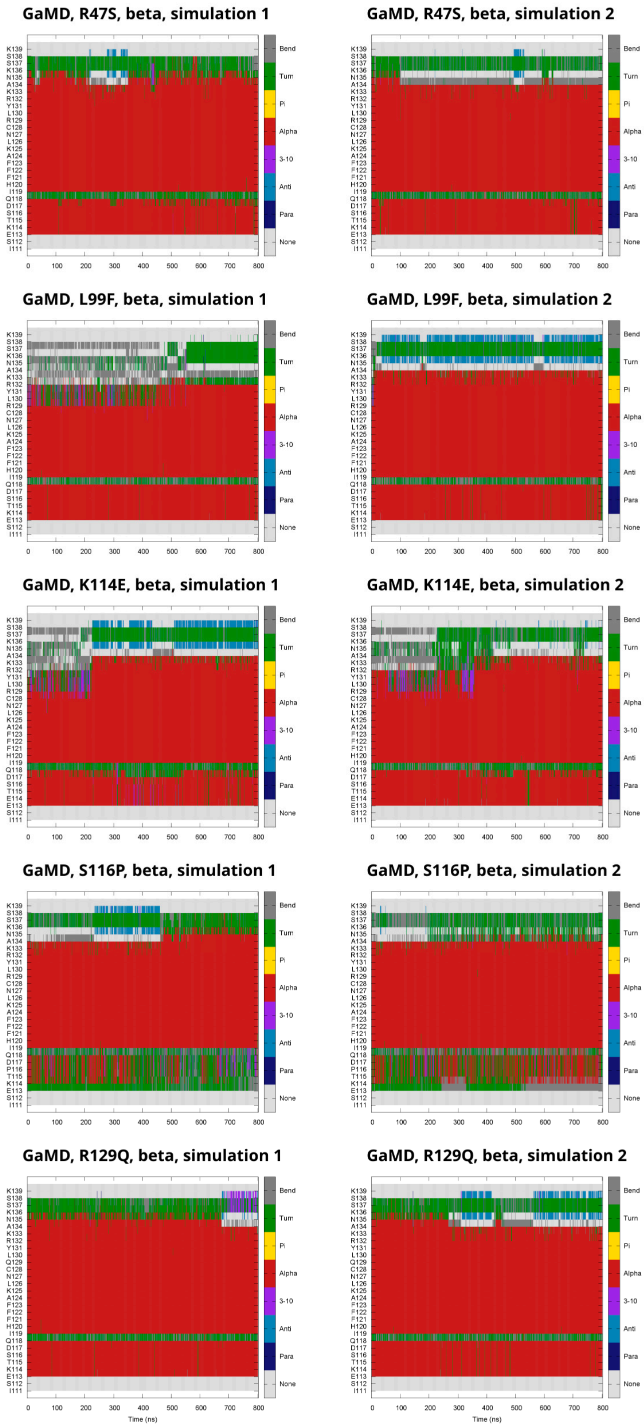

Supplementary Figure S35/B. Result of the DSSP analysis for residues 111-139, close to the heparin binding site, from the GaMD simulations of beta AT. In this figure, results from simulations of the R47S, L99F, K114E, S116P and R129Q mutants are shown.

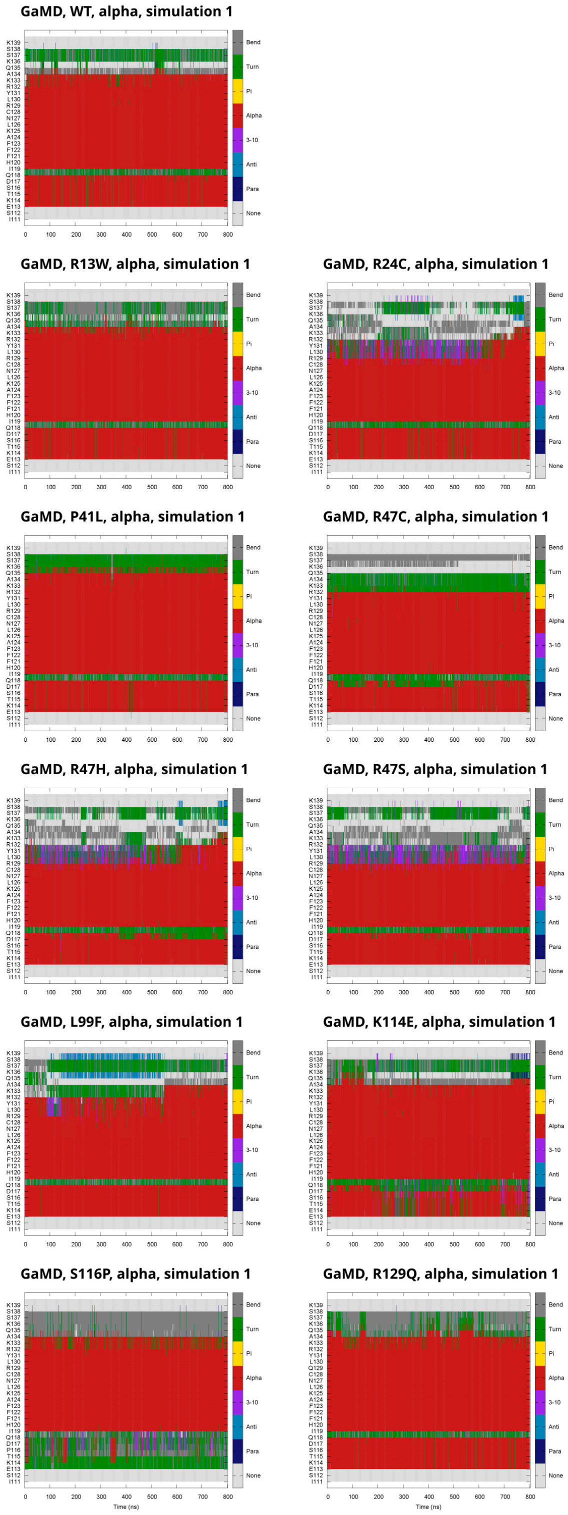

Supplementary Figure S36. Result of the DSSP analysis for residues 111-139, close to the heparin binding site, from the GaMD simulations of alpha AT.

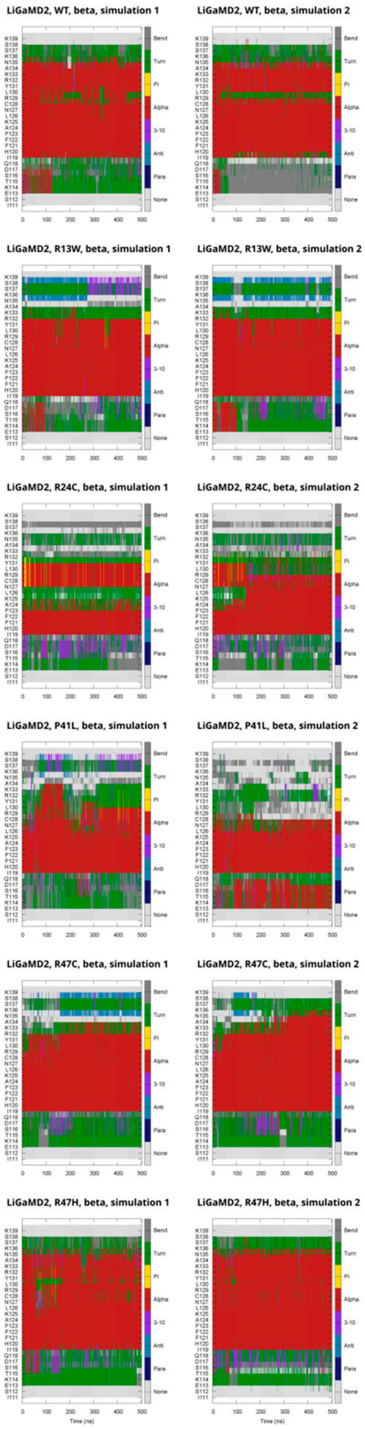

Supplementary Figure S37/A. Result of the DSSP analysis for residues 111-139, close to the heparin binding site, from the LiGaMD2 simulations of beta AT. In this figure, results from simulations of the WT protein as well as the R13W, R24C, P41L, R47C and R47H mutants are shown.

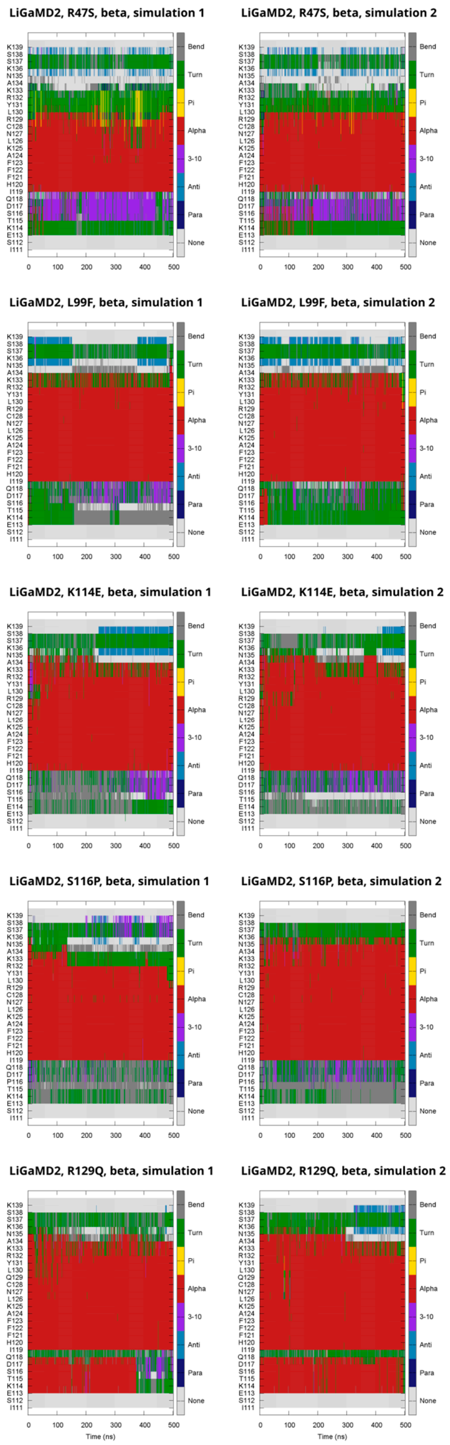

Supplementary Figure S37/B. Result of the DSSP analysis for residues 111-139, close to the heparin binding site, from the LiGaMD2 simulations of beta AT. In this figure, results from simulations of the R47S, L99F, K114E, S116P and R129Q mutants are shown.

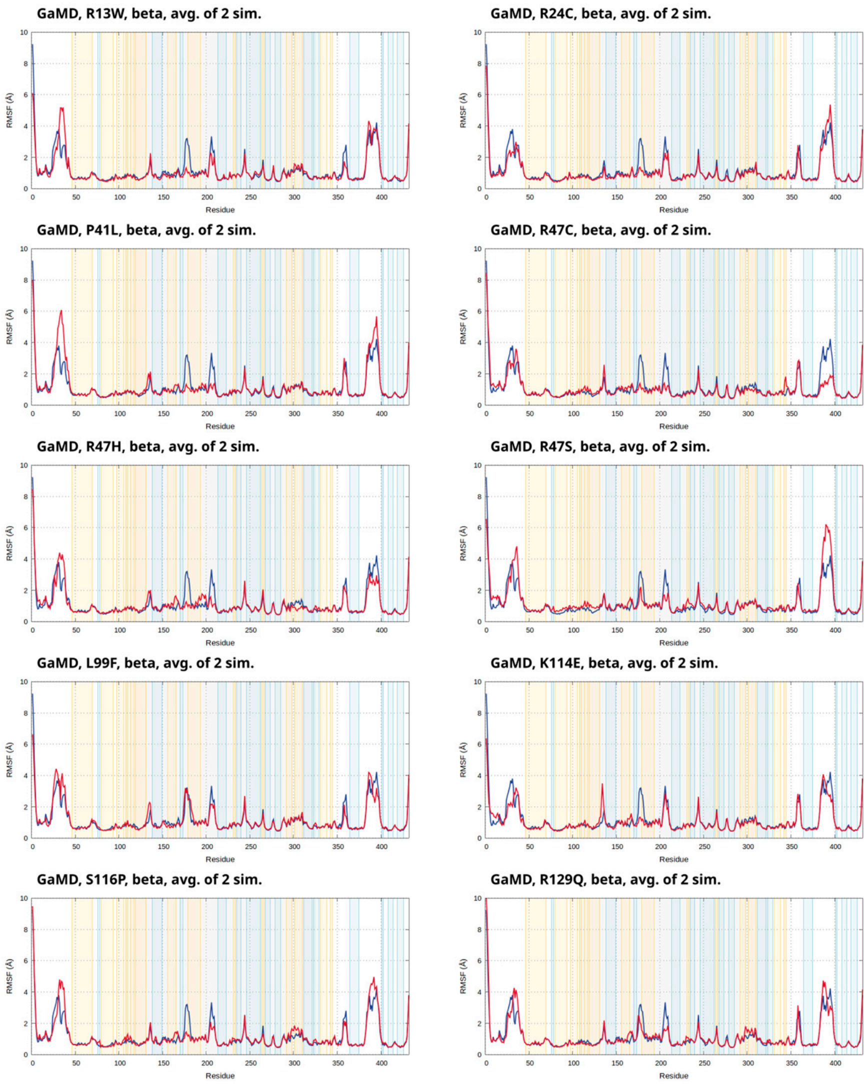

Supplementary Figure S38/A. Root mean fluctuations of the alpha-carbon atoms calculated from the GaMD simulation of each mutant, compared to the simulations of the WT system. The RMSF values for each CA atom was averaged from the two simulations for each mutant as well as the WT. The data for the mutants and the WT system are shown in red and blue, respectively.

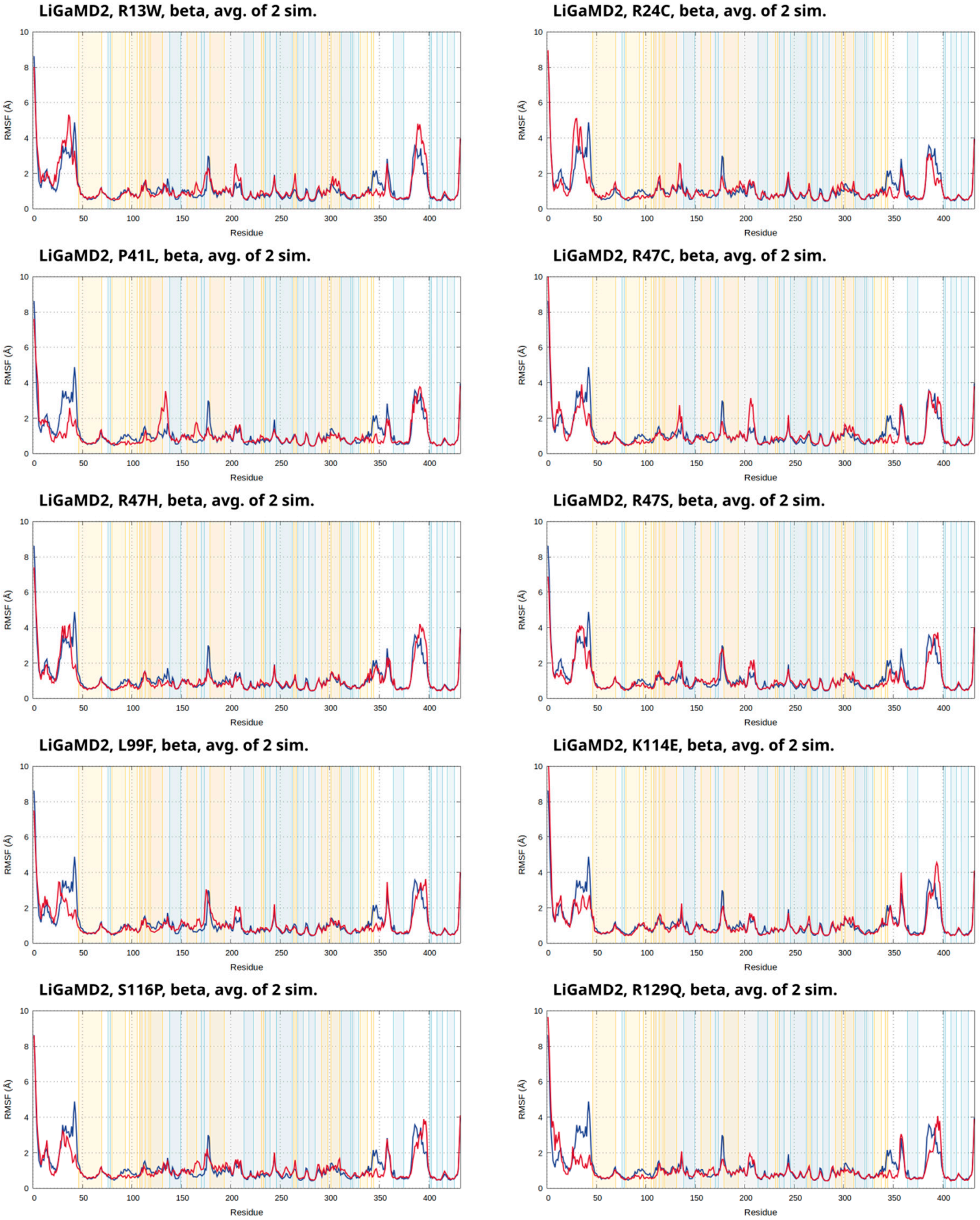

Supplementary Figure S38/B. Root mean fluctuations of the alpha-carbon atoms calculated from the LiGaMD2 simulation of each mutant, compared to the simulations of the WT system. The RMSF values for each CA atom was averaged from the two simulations for each mutant as well as the WT. The data for the mutants and the WT system are shown in red and blue, respectively.

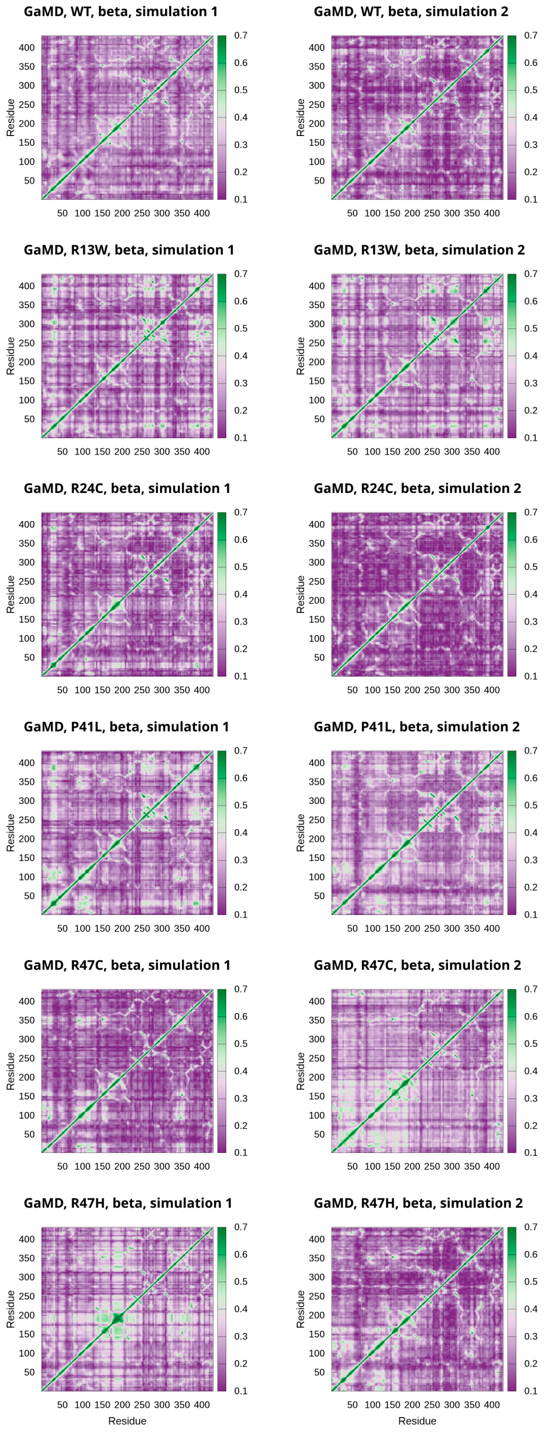

Supplementary Figure S39/A. Correlated motions of the alpha-carbon atoms in the GaMD simulations of the WT AT protein and the mutants. A method proposed by Lange and Grubmüller was used for the analysis. In this figure, results from simulations of the WT protein as well as the R13W, R24C, P41L, R47C and R47H mutants are shown.

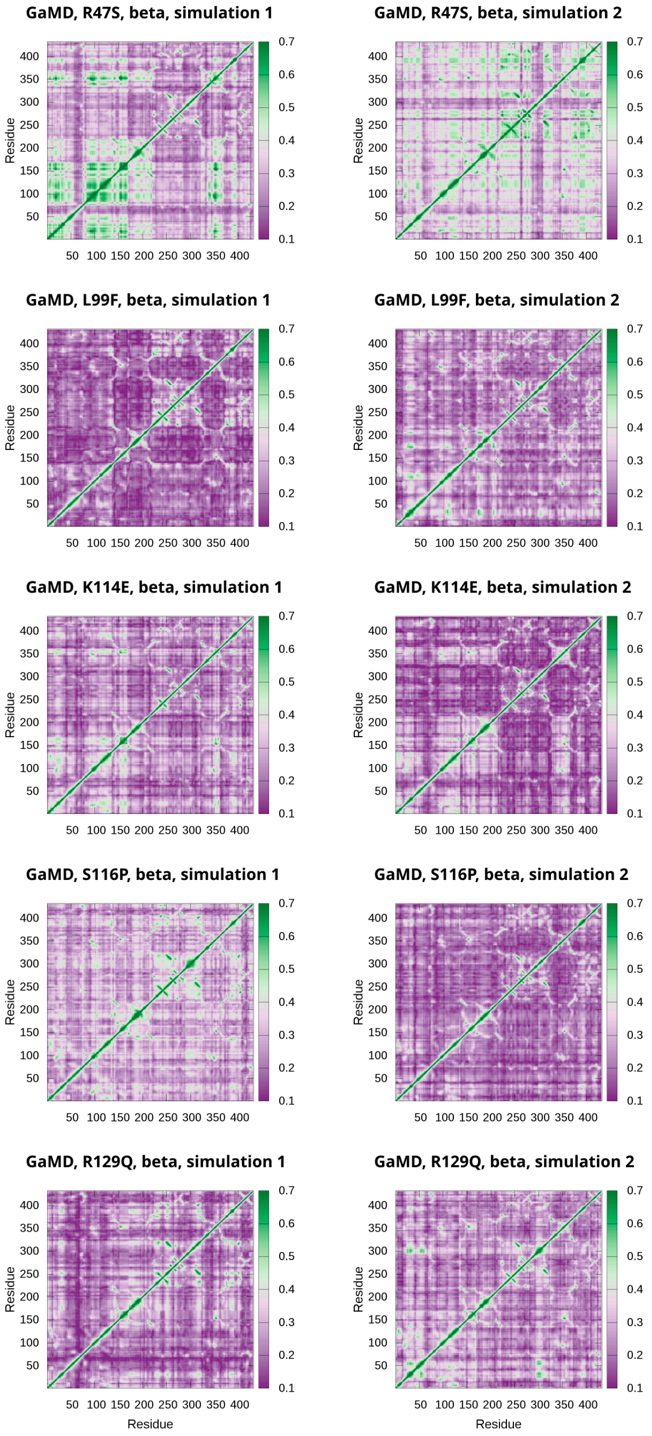

Supplementary Figure S39/B. Correlated motions of the alpha-carbon atoms in the GaMD simulations of the WT AT protein and the mutants. A method proposed by Lange and Grubmüller was used for the analysis. In this figure, results from simulations of the R47S, L99F, K114E, S116P and R129Q mutants are shown.

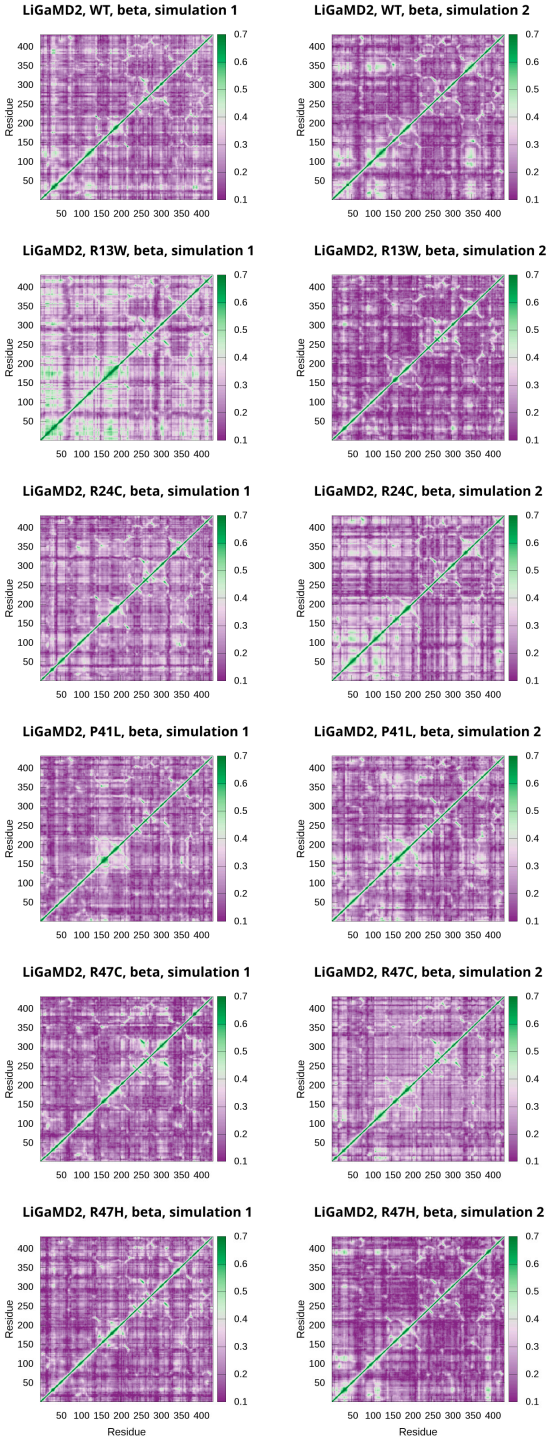

Supplementary Figure S40/A. Correlated motions of the alpha-carbon atoms in the LiGaMD2 simulations of the WT AT protein and the mutants. A method proposed by Lange and Grubmüller was used for the analysis. In this figure, results from simulations of the WT protein as well as the R13W, R24C, P41L, R47C and R47H mutants are shown.

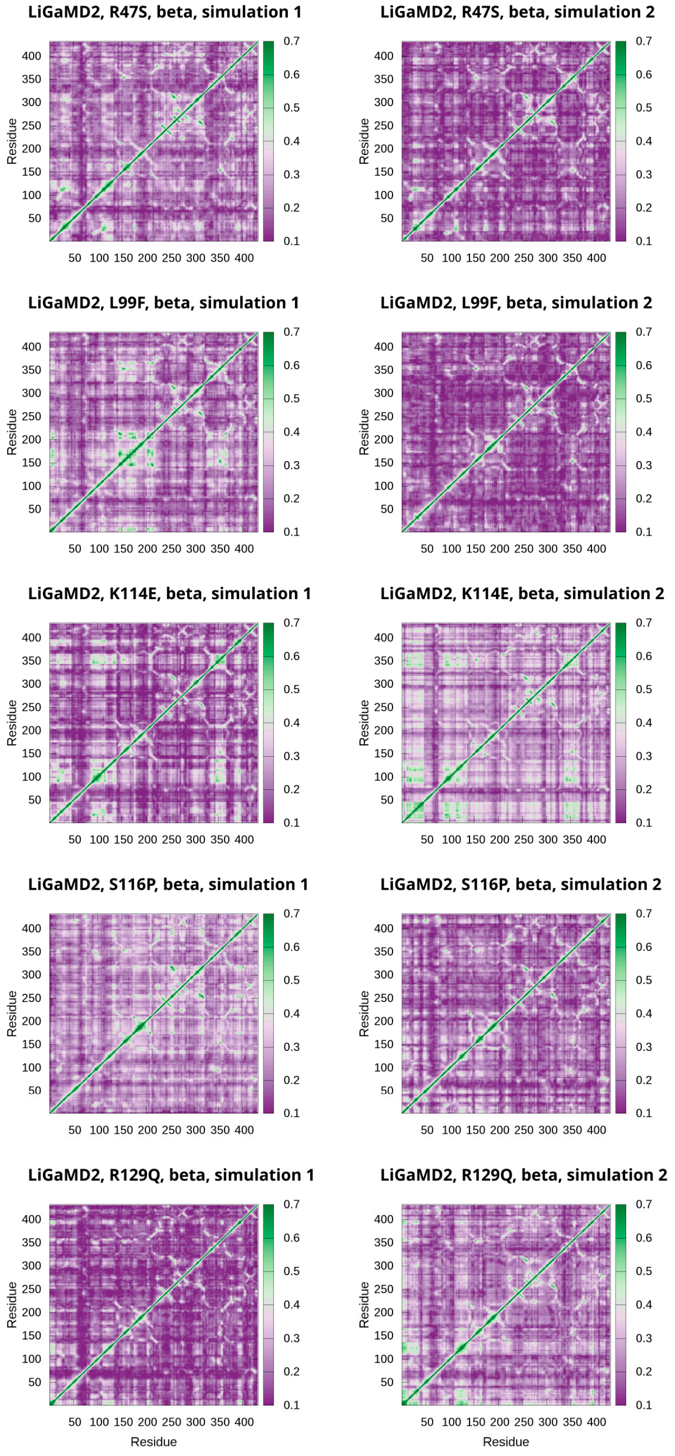

Supplementary Figure S40/B. Correlated motions of the alpha-carbon atoms in the LiGaMD2 simulations of the WT AT protein and the mutants. A method proposed by Lange and Grubmüller was used for the analysis. In this figure, results from simulations of the R47S, L99F, K114E, S116P and R129Q mutants are shown.

	RMSD	RMSD, D	RMSD, F	RMSD, H
	Beta-antithrombin, GaMD, 1600 frames analyzed			
WT	99.6%	98.2%	100.0%	92.9%
R13W	99.1%	99.8%	98.8%	90.6%
R24C	99.9%	99.7%	99.8%	96.7%
P41L	98.1%	96.3%	98.4%	93.9%
R47C	15.6%	23.4%	61.7%	3.8%
R47H	70.5%	89.9%	97.0%	33.8%
R47S	54.9%	88.7%	75.2%	32.3%
L99F	99.6%	96.8%	99.9%	96.4%
K114E	34.5%	97.4%	95.9%	20.6%
S116P	98.7%	98.8%	99.7%	77.6%
R129Q	99.9%	99.7%	99.9%	98.1%
	Alpha-antithrombin, GaMD, 800 frames analyzed			
WT	98.4%	98.3%	99.5%	93.9%
R13W	100.0%	99.9%	100.0%	97.3%
R24C	99.3%	93.3%	99.3%	96.9%
P41L	100.0%	93.1%	100.0%	99.9%
R47C	63.6%	100.0%	98.8%	15.5%
R47H	72.1%	99.8%	99.8%	13.4%
R47S	69.8%	99.9%	94.6%	27.9%
L99F	99.5%	98.6%	100.0%	90.6%
K114E	30.3%	66.1%	85.0%	2.8%
S116P	97.6%	98.3%	99.5%	53.6%
R129Q	100.0%	100.0%	100.0%	98.6%
	Beta-antithrombin, LiGaMD, 1000 frames analyzed			
WT	14.5%	10.5%	15.3%	18.2%
R13W	7.3%	4.4%	24.3%	13.8%
R24C	10.4%	3.2%	32.6%	23.7%
P41L	26.6%	50.2%	61.0%	17.5%
R47C	38.9%	87.1%	90.3%	6.2%
R47H	54.3%	78.1%	87.2%	20.8%
R47S	85.5%	83.5%	95.6%	57.2%
L99F	58.0%	75.7%	97.7%	41.3%
K114E	15.5%	57.0%	95.0%	4.6%
S116P	16.4%	73.0%	95.4%	5.4%
R129Q	28.7%	30.5%	62.7%	18.1%

Supplementary Table S1. RMSD of the pentasaccharide position in the energy minimized structure in the GaMD and LiGaMD2 simulations. The percentage of frames where the RMSD value was above 3 Å are shown.

	D.S6 – K125	E.C6 – K11	F.S2 – K114	F.S3 – K114	G.C6 – R47	G.C6 – K114	H.S2 – R47	H.S6 – K114
	Beta-antithrombin, LiGaMD, 3200 frames analyzed							
WT	75.6%	82.5%	99.9%	97.8%	100.0%	97.5%	98.8%	37.9%
R13W	72.4%	69.5%	99.7%	95.7%	99.9%	98.6%	96.1%	39.0%
R24C	37.4%	80.8%	99.8%	96.8%	99.8%	97.3%	97.6%	33.0%
P41L	36.6%	81.1%	99.9%	98.4%	100.0%	98.4%	98.4%	42.2%
R47C	31.3%	65.8%	65.7%	74.0%	0.0%	27.6%	0.0%	13.0%
R47H	23.4%	66.8%	87.9%	78.0%	0.0%	13.7%	0.0%	6.1%
R47S	52.2%	62.5%	79.1%	79.4%	0.0%	59.8%	0.0%	10.6%
L99F	56.1%	79.3%	99.9%	97.8%	99.7%	98.5%	98.8%	37.2%
K114E	20.3%	57.5%	0.0%	0.0%	72.0%	0.0%	31.7%	0.0%
S116P	70.3%	70.8%	98.5%	84.6%	99.5%	96.3%	98.2%	32.2%
R129Q	86.4%	35.6%	100.0%	95.6%	99.9%	97.9%	98.7%	30.2%
	Alpha-antithrombin, GaMD, 1600 frames analyzed							
WT	44.4%	84.9%	99.2%	96.0%	99.9%	95.6%	94.2%	35.6%
R13W	71.2%	55.4%	100.0%	97.1%	99.9%	96.8%	99.6%	40.8%
R24C	79.3%	99.8%	88.9%	99.9%	96.9%	98.1%	44.5%	0.0%
P41L	22.6%	71.3%	100.0%	98.7%	99.8%	98.6%	99.4%	32.7%
R47C	22.8%	46.1%	51.3%	89.1%	0.0%	4.4%	0.0%	0.0%
R47H	28.4%	50.6%	60.5%	43.6%	0.0%	3.1%	0.0%	6.4%
R47S	53.9%	64.7%	70.9%	83.6%	0.0%	18.9%	0.0%	1.1%
L99F	59.4%	77.0%	100.0%	89.3%	99.9%	99.4%	96.6%	39.9%
K114E	16.6%	82.8%	0.0%	0.0%	64.5%	0.0%	17.9%	0.0%
S116P	27.2%	74.4%	98.9%	46.5%	95.0%	95.8%	33.1%	12.1%
R129Q	87.8%	32.6%	100.0%	98.6%	99.9%	98.4%	98.6%	31.4%
	Beta-antithrombin, LiGaMD, 3200 frames analyzed							
WT	75.6%	82.5%	99.9%	97.8%	100.0%	97.5%	98.8%	37.9%
R13W	72.4%	69.5%	99.7%	95.7%	99.9%	98.6%	96.1%	39.0%
R24C	37.4%	80.8%	99.8%	96.8%	99.8%	97.3%	97.6%	33.0%
P41L	36.6%	81.1%	99.9%	98.4%	100.0%	98.4%	98.4%	42.2%
R47C	31.3%	65.8%	65.7%	74.0%	0.0%	27.6%	0.0%	13.0%
R47H	23.4%	66.8%	87.9%	78.0%	0.0%	13.7%	0.0%	6.1%
R47S	52.2%	62.5%	79.1%	79.4%	0.0%	59.8%	0.0%	10.6%
L99F	56.1%	79.3%	99.9%	97.8%	99.7%	98.5%	98.8%	37.2%
K114E	20.3%	57.5%	0.0%	0.0%	72.0%	0.0%	31.7%	0.0%
S116P	70.3%	70.8%	98.5%	84.6%	99.5%	96.3%	98.2%	32.2%
R129Q	86.4%	35.6%	100.0%	95.6%	99.9%	97.9%	98.7%	30.2%

SupplementaryTable S2. Selected interactions between amino acids of AT and negatively charged groups in the pentasaccharide (distance cutoff was 5 Å). The pairs where the mutants show the largest differences from the WT are listed in the table.
